# Associations between parental perceptions of neighbourhood environments and active travel to school: IPEN Adolescent study

**DOI:** 10.1186/s12966-025-01738-3

**Published:** 2025-05-15

**Authors:** Anna Timperio, Scott Duncan, Muhammad Akram, Javier Molina-García, Delfien Van Dyck, Anthony Barnett, Ferdinand Salonna, Anjana RM, James F. Sallis, Michal Vorlíček, Erica Hinckson, Kelli L. Cain, Terry L. Conway, Wan Abdul Manan Wan Muda, Mika Moran, Adewale L. Oyeyemi, Andreia Pizarro, Rodrigo S. Reis, Sheikh Muhammad Rezwan, Jasper Schipperijn, Ester Cerin

**Affiliations:** 1 Institute for Physical Activity and Nutrition, School of Exercise and Nutrition Sciences, Deakin University, Geelong, Australia; 2 School of Sport and Recreation, Faculty of Health and Environmental Sciences, Auckland University of Technology, Auckland, New Zealand; 3 Hudson Institute of Medical Research, Monash University, Clayton, Australia; 4 Department of Teaching of Physical Education, Arts and Music, AFIPS Research Group, University of Valencia, Valencia, Spain; 5 Epidemiology and Environmental Health Joint Research Unit, FISABIO-UJI-UV, Valencia, Spain; 6 Department of Movement and Sports Sciences, Faculty of Medicine and Health Sciences, Ghent University, Ghent, Belgium; 7 Mary Mackillop Institute for Health Research, Australian Catholic University, Melbourne, Australia; 8 Institute of Physical Education and Sport at P. J. Šafarik, Košice, Slovakia; 9 Institute of Active Lifestyle, Faculty of Physical Culture, Palacký University, Olomouc, Czech Republic; 10 Madras Diabetes Research Foundation, Chennai, India; 11 Herbert Wertheim School of Public Health, University of California San Diego, San Diego, USA; 12 Graduate School of Public Health, Alma Ata University, Yogyakarta, Indonesia; 13 School of Public Health, Faculty of Social Welfare and Health Sciences, University of Haifa, Haifa, Israel; 14 College of Health Solutions, Arizona State University, Phoenix, USA; 15 Research Centre in Physical Activity, Health and Leisure, Faculty of Sport, University of Porto, Porto, Portugal; 16 Laboratory for Integrative and Translational Research in Population Healthporto, Porto, Portugal; 17 People Health and Place Unit, Prevention Research Center, School of Public Health, Washington University in St. Louis, St. Louis, USA; 18 Department of Architecture, Bangladesh University of Engineering and Technology, Dhaka, Bangladesh; 19 Department of Sports Science and Clinical Biomechanics, University of Southern Denmark, Odense, Denmark; 20 School of Public Health, the University of Hong Kong, Hong Kong, Hong Kong SAR, China

**Keywords:** Active transport, School travel, Correlates, Perceived environment, Walking, Cycling, Walkability, Youth, Physical activity, International

## Abstract

**Background:**

Studies of correlates of active transport to and from school (ATS) focus mainly on children, have a limited conceptualisation of ATS trips, lack heterogeneity in built environments, and rarely consider effect modifiers. This study aimed to estimate associations of parent-perceived neighbourhood environment characteristics with self-reported ATS among adolescents from 14 countries, and whether associations differ by sex, city/region, and distance to school.

**Methods:**

Observational cross-sectional design. Data were from the International Physical activity and Environment Network (IPEN) Adolescent study and included 6302 adolescents (mean age 14.5 ± 1.7 years, 54% girls) and a caretaker from 16 diverse sites. Adolescents self-reported usual travel to and from school by walking and bicycling (days/week) and time it would take to walk. Parents completed the Neighbourhood Environment Walkability Scale for Youth (13 scores computed). Generalised additive mixed models estimated associations of parent neighbourhood perceptions with 1) any active transport to/from school, 2) regular walking (5–10 times/week), 3) regular cycling to/from school, and 4) profiles of ATS generated using latent profile analyses. Interactions were also explored.

**Results:**

Overall, 58.7% reported any ATS, 39.9% regularly walked, 7.7% regularly cycled, and four profiles of ATS were identified: walk to and from school; walk from school; cycle to and from school; no ATS. Distance to school was negatively associated with all outcomes, though evidence was weak for regular cycling to/from school. Land use mix – diversity was positively related to all ATS outcomes except those related to cycling. Accessibility and walking facilities were associated with higher odds of any ATS, regular walking to/from school, and the profile walking to and from school. Residential density was negatively related to regular cycling to/from school. Positive associations were observed between traffic safety and any ATS, and between safety from crime, aesthetics, and odds of regular cycling to/from school. Distance to school, adolescent sex, and city moderated several associations.

**Conclusions:**

Parent perceptions of compact, mixed-use development, walking facilities, and both traffic and crime-related safety were important supportive correlates of a range of ATS outcomes among adolescents in high- and low-middle-income countries. Policies that achieve these attributes should be prioritised to support more widespread ATS.

**Supplementary Information:**

The online version contains supplementary material available at 10.1186/s12966-025-01738-3.

## Background

Regular physical activity is associated with positive health outcomes in young people, including mental health [1], motor skills [2], cardiovascular fitness [3], cardiometabolic biomarkers [4], and lower risk of obesity [5]. Nonetheless, the prevalence of insufficient physical activity among adolescents (11–17 years) in 146 countries was 81% in 2016 [6]. Active transport to/from school (ATS), primarily walking and bicycling, provides an opportunity for regular physical activity and is widely promoted as a strategy to redress declining physical activity in this age group. Active travel can make a meaningful contribution to overall physical activity in young people [7–9], with a recent meta-analysis finding ATS could contribute almost half the physical activity required to meet current recommendations on weekdays [10]. ATS also has the potential to contribute to environmental sustainability targets by reducing motorised vehicle traffic before and after school [11].

Rates of ATS vary widely across the world. High-income countries (HICs) have lower levels of ATS than low-to-middle-income countries (LMICs), likely due to variation in car ownership and/or public transport service [12, 13]. There is also significant variation among countries within the same regions. Across 31 countries in Asia, for example, rates of ATS ranged from 18% to 84% [14], while a comparison of four European countries found between-country differences in rates of walking (30–55%) and cycling (1–22%) to/from school [15]. Despite this wide variation, associations between ATS and physical activity appear relatively consistent across countries [12].

Characteristics of the neighbourhood built environment are likely to be important facilitators or barriers to ATS. A review of 54 studies of children and adolescents found shorter distances to destinations, lower-traffic/higher-safety neighbourhoods, pedestrian infrastructure for walking and cycling, and ‘walkability’ (an index of residential density, street connectivity, and land use mix) were associated with greater active travel generally [16]. Despite greater autonomy to travel independently and potential for ATS to contribute to overall physical activity [9, 17], few studies in that review focused on school travel among adolescents specifically, and the studies that did were conducted in HICs [16]. Compared to children, findings among adolescents were generally less consistent. An overview of reviews of studies conducted only in HICs identified mostly mixed associations between many neighbourhood environment attributes (traffic safety, street connectivity, land-use mix, population/residential density, proximity/access to destinations) and transport-related physical activity specifically among adolescents [18]. Consistent negative associations were found for distance to school and (perhaps counterintuitively) aesthetics, with consistent positive associations found for play streets or streets that provided space for physical activity [18]. The mixed findings may in part be due to methodological limitations, including low variation in single-city/country studies and inconsistencies in the conceptualisation, operationalization and measurement of neighbourhood attributes. It is likely the strength of associations differs depending on how far adolescents live from school [19]. Multi-country studies that use consistent methods, examine the moderating role of distance to school, and capture heterogenous neighbourhood environments across low-, middle-, and high-income countries are required to enable a better understanding of how environments and ATS are related.

A further shortcoming relates to conceptualisation of ATS, where distinct ATS modes (e.g., walking and cycling) and/or trip directions (i.e., to school and from school) are often combined, despite evidence that trips to and from school via these modes vary in both prevalence [20, 21] and potential physical activity gains [10, 21]. Transport cycling can be undertaken for longer distances than walking, often uses different infrastructure, may be perceived as higher risk and, accordingly, neighbourhood factors associated with the behaviours may also differ [22]. Neighbourhood exposures may also differ between trips to versus from school, given that trips home may more often involve other activities or stops [10]. As such, important behaviour-specific neighbourhood correlates may be missed by combining modes and trip directions. While travel mode and trip direction can be examined separately, novel ways of conceptualising ATS that combine these aspects of active travel without losing detail, such as data-driven clustering or latent profiling techniques that capture underlying patterns of behaviour based on multiple inputs may be insightful. These techniques have been used to identify groups of adolescents with distinct patterns of physical activity and sedentary behaviour [23], but few investigators applied these methods to understand patterns of travel behaviour specifically [20, 24]. Based on all travel modes, Barnett et al [20] identified seven distinct profiles of active travel among adolescents from Hong Kong based on travel modes to and from school, respectively, ranging from a more active, healthy and sustainable profile to a less active/healthy and sustainable profile. Similarly, based on all travel modes, Loa et al [24] identified six modality profiles that changed descriptively between pre-COVID-19 and the pandemic period among adults in Toronto. The profiles represented a variety of combinations of behaviours, recognising that many do not travel in the same way every day. It is possible the neighbourhood environment supports or inhibits specific patterns of active travel behaviour more so than others.

The present study aimed to estimate associations of parent-perceived neighbourhood environment characteristics with ATS among adolescents, and whether these associations differ by sex, study site (city/region), and distance to school. Using a heterogenous multi-country dataset, the International Physical Activity and Environment – Adolescent study (IPEN Adolescent), we generated distinct ATS profiles to explore how ATS modes and trips cluster across countries. Our intention is to provide novel information about environmental correlates of ATS in adolescents that can be used to inform residential development and health promotion initiatives in diverse settings and populations.

## Methods

IPEN Adolescent is an observational cross-sectional study of 6,950 adolescents aged 11–19 years from 18 cities/regions (study sites), representing 15 diverse countries. The full IPEN Adolescent methods have been described elsewhere [25]. Briefly, within each site, participants and a parent/guardian were recruited from neighbourhoods or schools selected from small administrative units within four neighbourhood types: high walkability/high SES; high walkability/low SES; low walkability/high SES; low walkability/low SES. The intent of the design was to maximise heterogeneity of built form and socioeconomic circumstance within each country. Surveys were administered to adolescents and parents by paper–pencil, online, or interview between 2009 and 2016. The present study includes data from participants in Australia, Bangladesh, Belgium, Brazil, Czech Republic (two sites), Denmark, Hong Kong SAR (China), India, Israel, Malaysia, Nigeria, Portugal (combination of five sites), Spain, and the USA (two sites). Specific cities are shown in Table 1. Details of recruitment strategies within each country are available elsewhere [25]. Data collected in New Zealand were excluded as parent surveys were not administered.

**Table 1:** Overall and site-specific socio-demographic characteristics

		**High-income countries**											**Low-middle-income countries**				
	**All sites**	**Australia Melb**	**Belgium Ghent**	**Czech Rep HK**	**Czech Rep Olomouc**	**Denmark Odense**	**China Hong Kong**	**Israel Haifa**	**Portugal Various cities**	**Spain Valencia**	**USA Baltimore**	**USA Seattle**	**Bangladesh Dhaka**	**Brazil Curitiba**	**India Chennai**	**Malaysia KL**	**Nigeria Gombe**
**N**	6302	438	291	155	183	210	1295	232	184	465	485	443	92	493	316	752	268
**Child’s age (year)**																	
Mean	14.46	14.91	13.36	14.32	13.73	13.00	14.31	15.28	15.94	16.56	14.15	14.04	13.89	14.08	13.75	14.40	15.26
(SD)	(1.69)	(1.59)	(1.36)	(1.73)	(1.61)	(1.19)	(1.70)	(1.45)	(1.17)	(0.78)	(1.40)	(1.40)	(1.76)	(1.63)	(1.53)	(1.32)	(1.64)
% missing	0	0	0	0	0	0	0	0	0	0	0	0	0	0	0	0	0
**Child’s sex**																	
% male	46.46	40.18	41.92	50.32	44.81	40.95	42.78	39.22	37.50	44.95	46.60	52.82	53.26	48.88	52.53	53.06	54.48
%missing	0	0	0	0	0	0	0	0	0	0	0	0	0	0	0	0	0
**Highest education in the household**																	
% ≥College degree	46.67	35.39	73.54	32.26	15.85	66.67	35.29	60.78	35.87	55.70	73.81	76.07	58.70	40.77	47.15	24.87	53.73
% missing	11.22	45.66	1.72	36.13	54.10	3.33	0	1.29	18.48	0	1.03	0.23	0	0	0.32	38.16	3.36
**Area level SES**																	
% High	47.11	45.89	51.89	49.68	44.26	53.81	46.49	50.43	52.72	53.55	50.93	49.21	44.57	43.20	48.10	39.89	41.04
% missing	0	0	0	0	0	0	0	0	0	0	0	0	0	0	0	0	0
**Parental marital status**																	
% married/LWP	75.47	43.84	84.88	48.39	36.61	83.81	89.65	78.45	65.22	78.71	79.59	87.58	94.57	72.01	95.57	57.18	82.84
% missing	10.73	43.38	0.69	36.77	51.37	0.48	0	0.43	18.48	0	1.03	0.23	1.09	0	0	37.23	3.73
**Household size**																	
Mean	4.67	4.28	4.31	3.87	3.90	4.23	4.16	4.34	3.78	3.77	4.30	4.22	5.93	4.26	4.56	5.66	12.11
(SD)	(2.62)	(1.09)	(1.07)	(1.21)	(1.08)	(1.29)	(1.16)	(1.15)	(1.15)	(0.86)	(1.42)	(1.22)	(4.04)	(1.31)	(1.72)	(1.97)	(7.08)
% missing	9.25	45.43	4.81	1.94	0	3.81	0	2.16	20.11	0.22	0.82	0	3.26	0	2.53	38.16	5.22
**Children in the household**																	
Mean	2.08	2.03	2.25	1.69	1.78	2.22	1.67	1.96	1.50	1.45	2.08	1.97	2.37	1.85	1.81	2.48	5.98
(SD)	(1.56)	(0.91)	(1.25)	(0.74)	(0.71)	(1.12)	(0.75)	(0.97)	(0.95)	(0.58)	(1.21)	(0.96)	(1.66)	(0.98)	(0.69)	(1.35)	(4.08)
% missing	10.04	45.89	3.78	4.52	0.55	2.86	0	1.72	28.80	3.87	0.82	0	0	0.41	6.01	38.96	5.22
**Motor vehicle in the household**																	
% 0	20.63	0.91	4.81	5.16	4.92	7.62	69.19	11.21	5.98	7.31	2.06	0.90	68.48	18.86	23.42	1.33	10.45
% 1	27.21	15.07	38.49	31.61	23.50	48.10	22.86	41.81	26.09	45.59	15.88	9.26	25.00	41.99	46.84	14.23	32.84
% 2	25.83	26.71	42.61	19.35	11.48	35.24	6.18	37.93	38.59	33.12	45.77	42.89	1.09	28.40	18.35	24.47	27.61
% 3+	14.74	12.33	12.37	4.52	2.73	5.71	1.78	7.76	8.70	13.98	34.85	46.73	5.43	10.75	6.96	22.47	25.37
% missing	11.58	44.98	1.72	39.35	57.38	3.33	0	1.29	20.65	0	1.44	0.23	0	0	4.43	37.50	3.73
**Licensed driver in the household**																	
% 0	13.71	0.23	1.72	1.94	1.64	3.33	39.46	6.47	2.72	3.23	1.24	0.23	66.30	16.43	22.15	0.93	27.24
% 1	23.48	10.50	18.90	12.90	13.66	21.43	32.28	25.43	15.22	26.02	11.75	7.00	26.09	30.22	55.70	14.63	43.28
% 2	38.08	32.88	73.20	35.48	23.50	62.86	26.25	42.67	49.46	51.18	57.11	60.50	1.09	38.95	17.72	27.79	15.67
% 3+	13.42	11.42	4.81	10.32	5.46	9.05	2.01	25.00	13.59	19.57	28.45	32.05	5.43	14.40	3.16	19.28	9.70
% missing	11.30	44.98	1.37	39.35	55.74	3.33	0	0.43	19.02	0	1.44	0.23	1.09	0	1.27	37.37	4.10

*KL* Kuala Lumpur, *Melb* Melbourne, *Rep* Republic, *HK* Hradec Kralove, *SES* Socio-economic-status, *SD* Standard deviation, *LWP* living with partner

### Measures

#### Active travel to/from school

Adolescents reported the number of days in a usual week they travelled to school and from school, respectively, by walking and by bicycling [26, 27] (possible range: 0–5 days for each of the four variables). Trips to and from school were summed for each mode and dichotomised (0–4 times/week vs 5–10 times/week) to indicate regular use. Any active travel to/from school was also computed.

#### Parent-perceived neighbourhood environment

Parents completed the Neighbourhood Environment Walkability Scale for Youth (NEWS-Y) [28], with wording adapted within each country as needed. Subscales were derived based on a scoring protocol and measurement model developed specifically for IPEN Adolescent that incorporated items common to all countries and maximised between-country comparability, with consideration of relevance to adolescents (described below) [29]. Development of the measurement model, including confirmatory factor analyses and construct validity of the subscales, have been published elsewhere [29].

As a measure of *Residential density*, parents reported how common six types of homes were in their neighbourhood [response options for scoring: none (0), a few (1), some (2), most (3), all (4)]. The six items were weighted and summed (weightings shown in parentheses): detached single-family residences (1); multi-family houses of 1–3 stories (11); multi-family houses of 1–3 stories (25); multi-family houses of 7–12 stories (50); multi-family houses of 13–20 (75); and multi-family houses of over 20 stories (100). Due to a lack of houses over 20 stories, the last item was not included in Denmark.

Parents reported how long it would take them to walk to 13 types of destinations from their home (nearest of each), as well as to nine recreation facilities. Response options (scoring in parentheses) were: 1–5 min (5); 6–10 min (4); 11–20 min (3); 21–30 min (2); 31+ minutes (1); don’t know (1). ‘Don’t know’ is commonly coded as equivalent to 31+ minutes as it is likely the destination is further than a 30-min walk if the respondent is not aware of it being within walking distance. Perceived time to walk to the child’s own school and to a bus, subway or train stop were examined as single items as proxies for *distance to school* and *transit stop proximity*, respectively. A* Land Use Mix (LUM) diversity score* (excluding transit stops) was computed by averaging 12 of the 13 destinations: convenience store or equivalent; supermarket; laundry/dry cleaner; library; post office; bank/credit union; pharmacy/drug store; any school; the child’s school; fast food restaurant; coffee place; non-fast food restaurant. A *Recreational facilities* score was computed by averaging seven recreation facilities: indoor recreation/exercise facility; beach/lake/river/creek; bike/hiking/walking trails/paths; basketball court; other playing fields (e.g., soccer, skate park); swimming pool; school with recreation facilities open to the public. A *Park proximity* score was computed by averaging responses for small public park and large public park.

The following scales were constructed by averaging relevant items: *Accessibility and walking facilities* (5 items: hilly streets, fewer cul-de-sacs, many different routes, sidewalks available, separated sidewalks); *pedestrian infrastructure and safety* (3 items: lighting, visibility of walkers/bikers from homes, crosswalks/signals); *traffic safety* (3 items: difficult/unpleasant to walk due to traffic, traffic speed, drivers exceed speed limit); *aesthetics* (3 items: interesting things, beautiful/natural things, building/home nice to look at); and *safety from crime* (4 items regarding fear of child being hurt by a stranger in different situations). The following items from the NEWS-Y were treated as single items: presence of *trees* along streets; *buffer between streets and sidewalks* (grass/dirt); *difficult parking* in shopping areas. Responses were provided on a 4-point scale: strongly disagree (1) to strongly agree (4). Responses to corresponding items were averaged to compute each subscale (reverse scored as needed). Higher scores indicated higher walkability and safety.

#### Socio-demographics

Parents reported their child’s date of birth, sex, highest level of education attained by the most educated adult in the household (dichotomised: attained a college degree or higher vs. less than a college degree), marital status (dichotomised: married/with partner vs not married/no partner), and number of driveable motor vehicles, number of licensed drivers, people and children (<18 years), respectively, in the household. Design variables were also included: site and area-level SES (low vs high income).

### Data analytic plan

Descriptive statistics were computed for the full sample and each study site (*n* = 6,302). Latent profile analyses (LPA) [30] were conducted to identify groups of adolescents with different profiles of ATS (hereafter, latent profiles of ATS) based on reported frequency/week of walking to school, walking from school, cycling to school, and cycling from school. The optimal number of profiles was determined using the Bayesian Information Criterion (BIC) [31], Akaike Information Criterion (AIC) [32], and sample-size adjusted BIC (SABIC); smaller values indicated a better fitting model. The entropy measure of classification uncertainty was also examined, where values approaching 1 denote a high degree of separation, and values > 0.70 were considered acceptable [33]. We explored models with two to five profiles. Combined best fit and separation criteria were used to determine the optimal model (see Supplementary Material for details).

To estimate associations between parent-perceived neighbourhood attributes and adolescents’ ATS outcomes, we used generalised additive mixed models (GAMMs; package ‘mgcv’ version 1.8.34 in R [35]) with random intercepts at the administrative unit and school levels reflecting the two-stage sampling strategies. Outcomes were binary or multinomial variables and included (1) any ATS; (2) regular walking to/from school; (3) regular cycling to/from school; and (4) latent profiles of ATS. GAMMs with binomial variance and logit link functions were used to estimate the effects of environmental attributes on the odds of a specific outcome (odds ratios, ORs). Smooth terms (thin plate splines) were employed to model curvilinear associations, and evidence of curvilinearity was based on the comparison of AIC values from models with smooth vs. linear terms (10-unit difference in AIC) [35]. The moderating effects of adolescent sex, study site, and distance to school on the environment-outcome associations were estimated by adding two-way interaction terms to the corresponding main effect GAMMs. Statistically significant interaction effects were probed by estimating sex-specific, site-specific, and distance-to-school-specific associations. For city/site, AIC values of models with and without interaction terms were compared to determine if there was moderation.

To build GAMMs of total and direct effects of environmental attributes on ATS outcomes, directed acyclic graphs (DAGs) (Figure S1) identifying minimal sufficient sets of confounders (Table S1) were employed. In this study, the term ‘effect’ ought to be interpreted with caution due to the cross-sectional observational nature of the data and the likelihood of unmeasured confounders. Total and direct effects refer, respectively, to associations between an exposure and an outcome unadjusted and adjusted for measured mediators.

A quarter of cases had missing data on one or more variables. These data were not completely missing at random because missingness was related to study site (*p* < 0.001), and adolescents with missing data were older (*p* = 0.014), more likely to reside in lower SES areas (*p* = 0.007), not engage in ATS (*p* < 0.001), be from households with lower education (*p* < 0.001), and live with single parents (*p*= 0.005). Consequently, regression analyses were conducted on 20 imputed datasets developed using multiple imputations by chained equations (package ‘mice’) in R [36], following van Burren’s model-building recommendations [37]. For evaluation purposes, analyses were also conducted on cases with complete [37] data (*N* = 4,725), which we report in the supplementary material (Tables S21-S50).

## Results

### Sample description

Descriptive statistics for the overall sample and per site are presented in Table 1. While the distributions of adolescents’ sex and area-level SES were relatively balanced across all sites, substantial between-site differences were observed for highest level of parent education, number of children/household members, and number of motor vehicles and licenced drivers in the household (Table 1). For example, the average number of children in the household was 1.5 or below in the Portuguese and Spanish samples and nearly 6.0 in Gombe (Nigeria) (Table 1).

Three in five (59%) of adolescents reported engaging in any ATS (Table 2). However, the prevalence varied markedly by study site, ranging from over 90% in Odense, Denmark (91%) and Valencia, Spain (92%), to 40% or lower at the US sites (Baltimore 32%; Seattle 40%). Regular walking to/from school was particularly prevalent in Valencia (Spain) and Dhaka (Bangladesh), while regular cycling to/from school was most common in Ghent (Belgium) and Odense (Denmark). Adolescents tended to report a higher frequency of walking from school than walking to school, while differences in frequency between cycling from school and cycling to school were much smaller.

**Table 2:** Overall and site-specific outcome variables: active transport to/from school

		**High-income countries**											**Low-middle-income countries**				
	**All sites**	**Australia Melb**	**Belgium Ghent**	**Czech Rep HK**	**Czech Rep Olomouc**	**Denmark Odense**	**China Hong Kong**	**Israel Haifa**	**Portugal Various cities**	**Spain Valencia**	**USA Baltimore**	**USA Seattle**	**Bangladesh Dhaka**	**Brazil Curitiba**	**India Chennai**	**Malaysia KL**	**Nigeria Gombe**
**N**	6302	438	291	155	183	210	1295	232	184	465	485	443	92	493	316	752	268
**Walk to school (times/week)**																	
Mean	1.75	0.42	1.06	3.15	3.37	0.85	2.16	1.58	2.31	4.00	0.56	0.78	2.95	2.39	1.23	1.02	2.16
(SD)	(2.28)	(1.20)	(1.93)	(2.31)	(2.26)	(1.63)	(2.39)	(2.11	(2.34)	(1.87)	(1.43)	(1.66)	(2.24)	(2.44)	(2.12)	(1.88)	(2.38)
% missing	4.27	10.73	12.37	29.03	22.95	4.76	0	0	5.43	0	3.92	5.19	5.43	0.41	0	3.99	0
**Cycle to school (times/week)**																	
Mean	0.39	0.07	2.67	0.27	0.08	3.63	0.09	0	0.02	0.23	0.04	0.12	0.26	0.08	1.03	0.14	0.21
(SD)	(1.25)	(0.50)	(2.34)	(0.89)	(0.42)	(1.90)	(0.57)	(0)	(0.13)	(0.93)	(0.36)	(0.63)	(0.99)	(0.55)	(2.01)	(0.72)	(0.91)
% missing	4.25	10.73	11.34	29.03	22.95	4.76	0	0	7.07	0	3.92	5.19	5.43	0.41	0	3.86	0
**Walk home from school (times/week)**																	
Mean	2.17	2.07	1.12	3.59	3.53	0.95	2.30	2.28	2.66	4.15	1.02	1.26	3.14	2.73	1.44	1.74	2.47
(SD)	(2.31)	(2.17)	(1.94)	(2.02)	(2.11)	(1.67)	(2.34)	(2.27)	(2.32)	(1.71)	(1.79)	(1.93)	(2.17)	(2.41)	(2.23)	(2.17)	(2.40)
% missing	3.95	7.53	12.71	29.03	22.95	4.76	0	0	5.43	0	3.92	5.19	4.35	0.20	0	3.32	0
**Cycle home from school (times/week)**																	
Mean	0.41	0.46	2.65	0.30	0.08	3.57	0.08	0	0.02	0.32	0.05	0.14	0.16	0.08	1.04	0.11	0.19
(SD)	(1.29)	(1.26)	(2.33)	(0.97)	(0.40)	(1.92)	(0.57)	(0)	(0.24)	(1.06)	(0.36)	(0.74)	(0.70)	(0.55)	(2.01)	(0.66)	(0.89)
% missing	4.49	14.38	10.65	29.03	22.95	4.76	0	0	8.15	0	3.92	5.19	5.43	0.20	0	3.86	0
**Any walking trips to or from school**																	
% 1 or more	52.55	59.59	27.49	59.35	61.75	30.95	57.07	60.78	61.96	89.03	31.55	36.79	71.74	60.45	31.96	47.07	58.96
% missing	3.74	5.48	12.37	29.03	22.95	4.76	0	0	4.35	0	3.92	5.19	3.26	0.20	0	3.32	0
**Any cycling trips to or from school**																	
% 1 or more	10.81	13.70	53.95	9.03	3.83	81.90	3.24	0	2.17	10.32	2.68	4.97	7.61	2.64	21.52	5.05	5.97
% missing	4.11	9.36	10.65	29.03	22.95	4.76	0	0	7.07	0	3.92	5.19	5.43	0.20	0	3.86	0
**Any cycling or walking trips to or from school**																	
% 1 or more	58.73	60.05	71.82	61.29	62.30	90.95	57.99	60.78	62.50	91.83	32.37	39.50	72.83	61.66	50.63	48.54	62.31
% missing	3.60	5.48	9.62	29.03	22.95	4.76	0	0	3.80	0	3.92	5.19	3.26	0.20	0	3.32	0
**Regular walking to/from school**																	
% Yes	39.99	32.42	19.59	48.39	53.55	15.24	46.10	42.24	48.91	83.87	16.08	20.54	60.87	54.36	29.43	28.72	51.87
% missing	3.74	5.48	12.37	29.03	22.95	4.76	0	0	4.35	0	3.92	5.19	3.26	0.20	0	3.32	0
**Regular cycling to/from school**																	
% Yes	7.65	4.34	48.80	3.87	1.09	71.90	1.62	0	0	5.16	0.82	2.48	3.26	1.42	20.57	2.26	3.73
% missing	4.11	9.36	10.65	29.03	22.95	4.76	0	0	7.07	0	3.92	5.19	5.43	0.20	0	3.86	0

Regular walking or cycling to/from school defined as 5–10 times per week

*KL* Kuala Lumpur, *Melb* Melbourne, *Rep* Republic, *HK* Hradec Kralove, *SES* Socio-economic-status, *SD* Standard deviation

Parent-perceived neighbourhood environment characteristics varied across study sites with the greatest differences observed for average residential density, access to recreational facilities, park proximity, presence of trees, presence of buffers between roads and footpaths, and parking being difficult (Table 3). For example, Chennai (India) had an average score on the recreation facilities scale of 1.8, indicating a distance equivalent to a ~ 30-min walk from home, while the average score in Odense (Denmark) was 3.7, corresponding to a ~10-min walk. There were no parks within a 30-min walk from home in Gombe (Nigeria), while parks were reported to be within a 6–10-min walk from home in Valencia (Spain).

**Table 3:** Overall and site-specific perceived neighbourhood environment attributes

		**High-income countries**											**Low-middle-income countries**				
	**All sites**	**Australia Melb**	**Belgium Ghent**	**Czech Rep HK**	**Czech Rep Olomouc**	**Denmark Odense**	**China Hong Kong**	**Israel Haifa**	**Portugal Various cities**	**Spain Valencia**	**USA Baltimore**	**USA Seattle**	**Bangladesh Dhaka**	**Brazil Curitiba**	**India Chennai**	**Malaysia KL**	**Nigeria Gombe**
**N**	6302	438	291	155	183	210	1295	232	184	465	485	443	92	493	316	752	268
**Walking distance to school**																	
Mean	3.52	3.89	4.17	3.11	2.85	2.83	3.58	3.48	3.64	2.50	4.13	4.13	2.96	3.35	3.91	2.83	3.80
(SD)	(1.36)	(1.28)	(1.23)	(1.33)	(1.40)	(1.23)	(1.35)	(1.21)	(1.30)	(1.14)	(1.17)	(1.15)	(1.13)	(1.37)	(1.32)	(1.29)	(1.06)
% 1–5 min	8.47	6.85	5.15	9.68	14.21	13.81	7.95	7.33	5.98	21.29	4.12	3.61	10.87	11.16	4.75	8.78	2.61
% 6–10 min	14.30	8.22	5.84	14.84	23.50	25.71	16.68	11.21	14.67	30.75	6.19	6.77	21.74	18.26	14.56	11.04	6.34
% 11–20 min	22.01	18.04	11.00	19.35	14.75	30.95	23.09	34.48	26.09	32.04	17.11	15.58	38.04	25.56	19.30	15.69	32.09
% 21–30 min	13.09	17.58	12.03	12.26	8.74	10.95	14.21	19.40	12.50	8.60	14.43	16.93	17.39	14.40	8.23	5.05	25.00
% 31+ min	32.59	44.75	52.92	14.84	15.85	13.33	37.92	27.16	38.59	7.31	54.23	51.92	10.87	30.43	53.16	7.58	32.46
% missing	9.54	4.57	13.06	29.03	22.95	5.24	0.15	0.43	2.17	0	3.92	5.19	1.09	0.20	0	51.86	1.49
**Residential density**																	
Mean	213.5	48.86	71.59	155.80	111.80	106.10	468.40	216.80	121.55	251.10	38.67	23.83	177.30	96.30	65.64	292.0	271.50
(SD)	(221)	(103.77)	(109.0)	(114.6)	(98.39)	(109.96)	(203.2)	(148.5)	(93.97)	(134.67)	(57.90)	(33.73)	(82.87)	(126.68)	(77.79)	(230.36)	(159.36)
% missing	14.77	42.69	0.69	34.19	49.73	0	0	0.43	27.17	0	15.05	8.80	0	0	0	56.52	3.73
**LUM diversity (excluding transit stops)**																	
Mean	3.22	3.03	3.39	3.28	3.10	2.99	3.43	3.01	3.49	4.19	2.72	2.73	3.40	2.97	3.38	2.73	3.37
(SD)	(0.89)	(0.84)	(0.83)	(0.86)	(0.87)	(0.94)	(0.81)	(0.86)	(0.78)	(0.53)	(0.90)	(0.88)	(0.65)	(0.69)	(0.69)	(0.78)	(0.79)
% missing	12.55	42.69	1.03	34.19	49.73	0	0	0	27.17	0	0.41	0	1.09	0	0	52.53	3.36
**Recreation facilities (excluding parks)**																	
Mean	2.70	2.79	2.69	3.00	2.86	3.69	2.82	2.43	2.60	2.92	2.87	2.91	2.00	2.38	1.76	2.35	2.77
(SD)	(0.90)	(0.85)	(0.89)	(0.85)	(0.90)	(0.72)	(0.86)	(0.81)	(0.92)	(0.78)	(0.91)	(0.84)	(0.73)	(0.75)	(0.57)	(0.94)	(0.52)
% missing	12.57	42.69	1.72	34.19	49.73	0	0	0	27.17	0	0.41	0	1.09	0	0	52.39	3.36
**Accessibility and walking facilities**																	
Mean	2.99	3.25	2.98	3.24	3.09	3.08	2.99	3.10	2.97	3.58	2.97	2.82	2.83	2.88	2.59	2.77	2.74
(SD)	(0.58)	(0.48)	(0.58)	(0.45)	(0.51)	(0.46)	(0.50)	(0.51)	(0.38)	(0.42)	(0.57)	(0.65)	(0.57)	(0.64)	(0.59)	(0.44)	(0.65)
% missing	12.57	43.84	1.37	34.84	50.27	0	0	0.43	26.63	0	0.21	0	0	0	0	51.86	3.36
**Aesthetics**																	
Mean	2.52	2.96	2.27	2.27	2.22	2.66	2.47	2.55	2.38	2.25	3.04	3.12	1.81	2.38	1.52	2.53	2.91
(SD)	(0.83)	(0.76)	(0.69)	(0.59)	(0.66)	(0.76)	(0.68)	(0.82)	(0.54)	(0.74)	(0.71)	(0.66)	(0.79)	(0.87)	(0.82)	(0.64)	(0.85)
% missing	12.65	43.84	2.06	34.84	50.27	0	0	0.43	27.17	0	0.21	0	0	0	0	52.13	3.36
**Traffic safety**																	
Mean	2.61	2.89	2.51	2.89	2.80	2.94	2.82	2.36	2.78	2.61	2.51	2.66	2.37	2.17	2.27	2.37	2.95
(SD)	(0.67)	(0.61)	(0.59)	(0.55)	(0.58)	(0.71)	(0.50)	(0.70)	(0.46)	(0.72)	(0.59)	(0.57)	(0.60)	(0.77)	(0.68)	(0.53)	(0.88)
% missing	12.55	43.84	2.75	34.19	49.73	0	0	0	24.46	0	0.21	0	0	0	0	51.99	3.73
**Pedestrian infrastructure & safety**																	
Mean	2.85	2.81	2.68	3.03	2.93	2.90	2.95	2.90	2.89	3.03	2.80	2.87	2.46	2.55	2.92	2.70	2.96
(SD)	(0.66)	(0.55)	(0.58)	(0.61)	(0.50)	(0.70)	(0.56)	(0.74)	(0.50)	(0.62)	(0.66)	(0.64)	(0.59)	(0.79)	(0.80)	(0.58)	(0.83)
% missing	12.57	43.84	2.75	34.19	49.73	0	0	0	24.46	0	0.41	0	0	0	0	51.99	3.73
**Safety from crime**																	
Mean	2.81	3.14	3.10	2.88	2.80	3.67	2.69	3.25	2.98	3.25	2.95	3.07	1.97	2.04	3.03	2.05	2.72
(SD)	(0.93)	(0.78)	(0.76)	(0.73)	(0.72)	(0.59)	(0.87)	(0.86)	(0.55)	(0.79)	(0.72)	(0.73)	(0.86)	(0.84)	(1.10)	(0.71)	(1.15)
% missing	12.60	43.84	2.06	34.84	49.73	0	0	0.86	25.54	0.22	0.41	0	0	0	0	51.86	3.36
**Transit stop proximity**																	
Mean	4.11	4.53	4.64	4.33	4.25	4.61	3.91	4.50	4.56	4.77	3.63	4.03	2.15	4.74	3.79	3.19	4.03
(SD)	(1.16)	(0.76)	(0.76)	(1.11)	(1.01)	(0.72)	(1.08)	(0.88)	(0.77)	(0.57)	(1.44)	(1.22)	(1.25)	(0.62)	(1.11)	(1.38)	(1.02)
% missing	12.92	43.15	5.15	34.19	49.73	0	0	0.86	27.72	0	0.41	0	3.26	0	0.32	52.53	4.48
**Park proximity**																	
Mean	3.01	3.84	3.11	3.02	2.34	3.34	3.35	3.17	2.91	4.02	2.91	3.14	1.84	2.68	2.18	2.54	1.0^a^
(SD)	(1.25)	(0.96)	(1.28)	(1.12)	(1.20)	(1.27)	(1.07)	(1.18)	(0.97)	(0.83)	(1.21)	(1.15)	(0.93)	(0.97)	(1.07)	(1.14)	(0)
% missing	13.20	42.69	2.06	34.19	50.82	0	0	0	27.17	0	0.41	0	3.26	0	0	57.05	3.36
**Trees**																	
Mean	2.96	3.61	2.54	2.81	2.58	2.49	3.19	3.20	2.62	3.07	3.34	3.11	1.99	3.25	2.06	2.99	1.84
(SD)	(1.00)	(0.61)	(0.94)	(0.82)	(0.79)	(1.07)	(0.76)	(0.87)	(0.76)	(0.97)	(0.79)	(0.90)	(1.21)	(0.95)	(1.27)	(0.74)	(1.18)
% missing	12.73	43.84	3.44	34.84	50.82	0	0	0.86	26.63	0	0.21	0	0	0	0	52.13	3.36
**Buffers between street and footpath**																	
Mean	2.32	3.22	1.62	2.71	2.73	2.10	2.81	1.60	2.17	1.65	2.74	2.32	1.47	2.53	1.60	2.62	1.17
(SD)	(1.10)	(0.93)	(0.76)	(0.90)	(0.79)	(1.12)	(0.87)	(0.97)	(0.71)	(0.91)	(1.08)	(1.12)	(0.85)	(1.15)	(1.11)	(0.84)	(0.59)
% missing	12.77	44.29	2.41	34.84	50.27	0	0	0.86	26.63	0	0.21	0	1.09	0	0	52.13	4.85
**Parking difficult**																	
Mean	2.33	2.18	2.22	2.11	1.90	1.40	2.36	2.89	2.33	3.17	1.76	1.68	3.39	2.63	2.12	2.82	2.15
(SD)	(1.09)	(0.98)	(1.01)	(0.77)	(0.86)	(0.80)	(0.89)	(1.02)	(0.72)	(1.00)	(0.87)	(0.86)	(0.93)	(1.25)	(1.35)	(0.83)	(1.25)
% missing	12.87	43.84	3.09	34.84	51.91	0	0	2.16	26.63	0	0.21	0	1.09	0.20	0.63	52.26	3.36

^a^There is no variation in response, all the responses are 1

*KL* Kuala Lumpur, *Melb* Melbourne, *Rep* Republic, *HK* Hradec Kralove, *SES* Socio-economic-status, *LUM* Land use mix, *SD* Standard deviation, *min* minutes

Finally, cities varied in the amount of missing data on certain variables ranging, for example, from 0% (Hong Kong, China; Valencia, Spain; Dhaka, Bangladesh; Curitiba, Brazil) to 57% (Olomouc, Czech Republic) for number of motorised vehicles in the household (Table 1). The pooled descriptive statistics for the imputed dataset can be found in Tables S2 to S4.

### Latent profile analyses of active transport to/from school

A four-profile model of adolescents’ ATS with equal variances and covariance provided the best fit to the data according to both BIC and AIC values (Table S5). The entropy value of the four-profile model was 0.997, close to the theoretical maximum of 1, indicating a high degree of separation among the four profiles. Table 4 describes the four latent profiles in terms of average responses on the four items measuring weekly frequency of walking and cycling to/from school. The first profile was represented by adolescents regularly walking to and from school; the second profile encompassed adolescents who did not walk to school but regularly walked from school; the third profile included those who regularly cycled to/from school; the last profile denoted adolescents who did not engage or seldom engaged in ATS.

**Table 4:** Average weekly frequency of walking and cycling to/from school by latent profile of adolescents’ active transport to/from school

	**Latent profiles of adolescents’ active transport to/from school**			
**Type of active transport to/from school (survey items)**	Walking to and from school n_cc_ = 2023 n_mi_ = 2098	Walking from school n_cc_ = 581 n_mi_ = 610	Cycling to and from school n_cc_ = 452 n_mi_ = 479	No active transport to/from school n_cc_ = 2937 n_mi_ = 3115
Walking to school	4.85 (4.29, 5.40)	0.24 (− 0.32, 0.79)	0.56 (0.01, 1.12)	0.11 (− 0.45, 0.66)
Walking from school	4.59 (3.66, 5.51)	4.51 (3.59, 5.44)	0.66 (− 0.27, 1.58)	0.24 (− 0.68, 1.17)
Cycling to school	0.04 (− 0.26, 0.33)	0.04 (− 0.26, 0.33)	4.61 (4.31, 4.90)	0.03 (− 0.26, 0.33)
Cycling from school	0.11 (− 0.45, 0.68)	0.15 (− 0.42, 0.71)	4.40 (3.82, 4.95)	0.04 (− 0.53, 0.60)

Values represent means and their 95% confidence intervals (in brackets)

*cc* complete case, *mi* multiple imputations

### Parent-perceived neighbourhood environment and adolescents’ active transport to/from school

Tables 5 and 6 report the pooled direct main effects of parent-perceived neighbourhood environment characteristics on adolescents’ ATS outcomes. Distance to school was the most consistent correlate of ATS, showing negative associations with all outcomes, though the evidence of association was weak for regular cycling to/from school (Table 5). Land use mix – diversity was the second most consistent correlate and was positively related to all ATS outcomes except those pertaining to cycling. However, the evidence of association was weak for walking from school vs no ATS in the direct effect model (Table 6). Accessibility/walking facilities was positively associated with any ATS, regular vs occasional or no walking to/from school (Table 5) and walking to and from school vs no ATS (Table 6). Residential density was positively associated with walking to and from school (vs no ATS) and negatively related to regular cycling to/from school (Tables 5 and 6), though associations were stronger for total (minimally adjusted) than for direct effects for these outcomes (Tables S6 and S7). Positive associations were observed between traffic safety and any ATS, and between safety from crime, aesthetics and the odds of regular vs. occasional or no cycling (Table 5).

**Table 5:** Total and direct effects of parent-perceived neighbourhood environment characteristics on adolescents’ active transport to/from school (multiple imputations)

Neighbourhood characteristics [range of values]	Effect	Any active transport to/from school			Regular cycling to/from school^#^			Regular walking to/from school		
		OR	95% CI	*p*	OR	95% CI	*p*	OR	95% CI	*p*
Residential density [0–1000]	Total	**1.001**	**1.000, 1.001**	**.006**	**0.999**	**0.998, 1.00**	**.050**	**1.001**	**1.000, 1.001**	**.002**
	Direct	1.000	0.999, 1.001	.149	0.999	0.998, 1.00	.077	1.000	0.999, 1.001	.148
Land use mix – diversity^1^ [1–5]	Total	**1.38**	**1.26, 1.51**	**<.001**	1.09	0.93, 1.29	.284	**1.46**	**1.33, 1.59**	**<.001**
	Direct	**1.34**	**1.20, 1.49**	**<.001**	1.13	0.93, 1.39	.226	**1.44**	**1.30, 1.59**	**<.001**
Transit stop proximity [1–5]	Total	1.00	0.89, 1.13	.982	1.01	0.85, 1.19	.935	0.97	0.88, 1.07	.550
	Direct	1.00	0.88, 1.13	.951	1.01	0.85, 1.19	.919	0.96	0.87, 1.06	.460
Recreational facilities^2^ [1–5]	Total^a^	1.01	0.91, 1.13	.796	0.96	0.78, 1.18	.723	1.05	0.94, 1.16	.412
Park proximity [1–5]	Total	0.98	0.91, 1.06	.674	0.93	0.82, 1.06	.296	0.98	0.91, 1.05	.601
	Direct	0.98	0.91, 1.05	.541	0.92	0.80, 1.05	.211	0.98	0.91, 1.05	.493
Accessibility & walking facilities [1–4]	Total^a^	**1.15**	**1.00, 1.32**	**.050**	1.00	0.76, 1.31	.988	**1.18**	**1.01, 1.37**	**.036**
Traffic safety [1–4]	Total^a^	**1.13**	**1.00, 1.27**	**.044**	1.09	0.87, 1.36	.461	1.06	0.95, 1.19	.308
Pedestrian infrastructure [1–4]	Total^a^	1.08	0.97, 1.21	.176	1.03	0.82, 1.29	.800	1.10	0.97, 1.24	.128
Safety from crime [1–4]	Total^a^	0.96	0.84, 1.10	.566	**1.25**	**1.05, 1.49**	**.011**	0.92	0.79, 1.08	.333
Aesthetics [1–4]	Total^a^	1.00	0.87, 1.14	.955	**1.22**	**1.00, 1.48**	**.050**	0.96	0.85, 1.09	.533
Buffers between streets & footpath [1–4]	Total	0.98	0.91, 1.05	.499	0.97	0.84, 1.11	.647	0.99	0.92, 1.06	.720
	Direct	0.94	0.87, 1.02	.132	0.99	0.86, 1.15	.929	0.95	0.87, 1.02	.160
Parking difficult [1–4]	Total^a^	1.04	0.96, 1.12	.332	0.91	0.79, 1.05	.184	1.04	0.97, 1.13	.260
Trees [1–4]	Total	0.99	0.91, 1.07	.707	0.94	0.81, 1.09	.407	1.03	0.95, 1.11	.458
	Direct	0.97	0.89, 1.07	.545	0.89	0.76, 1.05	.181	1.03	0.94, 1.12	.555
Distance to school [1–5]	Total^a^	**0.39**	**0.36, 0.42**	**<.001**	0.91	0.83, 1.01	.081	**0.41**	**0.39, 0.44**	**<.001**

^1^excluding transit stops, ^2^ excluding parks, *OR* Odd ratio, *CI* Confidence intervals, *p* = *p*-value; in bold: effects significant at *p* < 0.05, ^#^ *N* = 5703 instead of 6302 because data from Israel, Portugal and Olomouc in the Czech Republic were excluded from the analyses due to 0% prevalence of regular cycling to/from school. The reference category of all outcome variables is ‘No’. Regular cycling/walking to/from school means cycling/walking to/from school 5–10 times a week. Analyses undertaken on 20 imputed datasets. ^a^Total and direct effects are equivalent as no mediating variables of characteristic-outcome associations were included in the models. Complete case analyses are in the Supplementary Material (Tables S21, S22 and S23). Model covariates were based on DAG depicted in Supplementary Materials (Fig S1). All models adjusted for adolescent age, adolescent sex, area-level SES, highest education in the household, number of children, number of adults and city. Additional adjustments per model included:

*Residential density models*: marital status. Direct effect: accessibility and walking facilities; aesthetics; buffers between street and footpath; land use mix – diversity^1^; number of driving license; number of motor vehicles; parking difficult; parks proximity; pedestrian infrastructure and safety; distance to school; recreational facilities^2^; safety from crime; traffic safety; transit stop proximity; trees

*Land use mix diversity models *(*excluding transit stops*): marital status; residential density. Direct effect: accessibility and walking facilities; aesthetics; buffers between street and footpath; number of driving license; number of motor vehicles; parking difficult; parks proximity; pedestrian infrastructure and safety; safety from crime; traffic safety; transit stop proximity; trees

*Transit stop proximity models*: land use mix diversity; residential density. Direct effect: accessibility and walking facilities; aesthetics; buffers between street and footpath; number of driving license; marital status; number of motor vehicles; parks proximity; pedestrian infrastructure and safety; recreational facilities^2^; safety from crime; traffic safety; trees

*Recreational facilities *(*excluding parks*): accessibility and walking facilities; aesthetics; buffers between street and footpath; land use mix diversity; number of driving license; marital status; number of motor vehicles; residential density; parks proximity; pedestrian infrastructure and safety; safety from crime; traffic safety; transit stop proximity; trees

*Park proximity*: land use mix diversity; residential density. Direct effect: accessibility and walking facilities; aesthetics; buffers between street and footpath; pedestrian infrastructure and safety; safety from crime; traffic safety; trees

*Accessibility and walking facilities*: aesthetics; buffers between street and footpath; land use mix diversity; residential density; parks proximity; pedestrian infrastructure and safety; safety from crime; traffic safety; trees; aesthetics; buffers between street and footpath; land use mix diversity; residential density; parks proximity; pedestrian infrastructure and safety; safety from crime; traffic safety; trees

*Traffic safety*: accessibility and walking facilities; aesthetics; buffers between street and footpath; land use mix diversity; residential density; parks proximity; pedestrian infrastructure and safety; safety from crime; trees

*Pedestrians infrastructure and safety*: accessibility and walking facilities; aesthetics; buffers between street and footpath; land use mix diversity; residential density; parks proximity; safety from crime; traffic safety; trees

*Safety from crime*: accessibility and walking facilities; aesthetics; buffers between street and footpath; land use mix diversity; residential density; parks proximity; pedestrian infrastructure and safety; traffic safety; trees

*Aesthetics*: accessibility and walking facilities; buffers between street and footpath; land use mix diversity; residential density; parks proximity; pedestrian infrastructure and safety; safety from crime; traffic safety; trees

*Buffers between street and footpath*: residential density. Direct effect: accessibility and walking facilities; aesthetics; land use mix diversity; parks proximity; pedestrian infrastructure and safety; safety from crime; traffic safety; trees

*Parking difficult*: accessibility and walking facilities; aesthetics; buffers between street and footpath; land use mix diversity; number of driving license; marital status; number of motor vehicles; residential density; parks proximity; pedestrian infrastructure and safety; recreational facilities no parks; safety from crime; traffic safety; transit stops; trees

*Trees*: residential density. Direct effect: accessibility and walking facilities; aesthetics; buffers between street and footpath; land use mix diversity; parks proximity; pedestrian infrastructure and safety; safety from crime; traffic safety

*Distance to school*: residential density

**Table 6:** Total and direct effects of parent-perceived neighbourhood environment characteristics on latent profiles of adolescents’ active transport to/from school (multiple imputations)

Neighbourhood characteristics [range of values]	Effect	Walking to & from school vs. no active transport [*n* = 5215]			Walking from school vs. no active transport [*n* = 3727]			Cycling to & from school vs. no active transport^#^ [*n* = 3389]			Walking to & from school vs. walking from school [*n* = 2708]		
		OR	95% CI	*p*	OR	95% CI	*p*	OR	95% CI	*p*	OR	95% CI	*p*
Residential density [0–1000]	Total	**1.001**	**1.000, 1.002**	**<.001**	1.000	0.999, 1.001	.338	0.999	0.998, 1.001	.239	1.001	0.999, 1.002	.059
	Direct	**1.001**	**1.000, 1.002**	**.047**	1.000	0.999, 1.001	.614	0.999	0.998, 1.001	.231	1.001	0.999, 1.002	.148
Land use mix – diversity^1^ [1–5]	Total	**1.57**	**1.42, 1.75**	**<.001**	**1.18**	**1.03, 1.35**	**.017**	1.19	0.99, 1.43	.068	**1.36**	**1.15, 1.60**	**<.001**
	Direct	**1.58**	**1.38, 1.79**	**<.001**	1.14	0.98, 1.33	.092	1.21	0.96, 1.52	.114	**1.36**	**1.12, 1.64**	**.002**
Transit stop proximity [1–5]	Total	0.93	0.83, 1.04	.204	1.04	0.88, 1.21	.666	1.03	0.85, 1.24	.789	0.93	0.80, 1.10	.403
	Direct	0.92	0.82, 1.03	.161	1.03	0.87, 1.21	.742	1.02	0.84, 1.24	.825	0.93	0.79, 1.10	.391
Recreational facilities^2^ [1–5]	Total^a^	1.04	0.91, 1.19	.545	1.06	0.90, 1.25	.481	0.93	0.73, 1.17	.528	1.01	0.84, 1.22	.902
Park proximity [1–5]	Total	0.98	0.90, 1.07	.621	1.00	0.90, 1.11	.973	0.92	0.79, 1.07	.293	1.00	0.89, 1.13	.958
	Direct	0.97	0.89, 1.05	.448	0.99	0.88, 1.11	.853	0.90	0.77, 1.06	.214	1.00	0.88, 1.12	.937
Accessibility & walking facilities [1–4]	Total^a^	**1.19**	**1.01, 1.40**	**.036**	1.20	0.97, 1.49	.095	1.08	0.80, 1.46	.620	0.99	0.77, 1.28	.966
Traffic safety [1–4]	Total^a^	1.13	0.99, 1.30	.081	1.07	0.89, 1.28	.484	1.05	0.83, 1.33	.704	1.10	0.89, 1.35	.381
Pedestrian infrastructure [1–4]	Total^a^	1.11	0.97, 1.26	.131	1.05	0.88, 1.26	.606	1.10	0.87, 1.41	.424	1.07	0.87, 1.31	.508
Safety from crime [1–4]	Total^a^	0.91	0.76, 1.08	.276	0.91	0.70, 1.19	.493	1.11	0.90, 1.36	.332	1.00	0.81, 1.22	.984
Aesthetics [1–4]	Total^a^	0.96	0.84, 1.10	.567	0.96	0.78, 1.18	.728	1.21	0.97, 1.52	.096	1.01	0.83, 1.23	.930
Buffers between streets & footpath [1–4]	Total	1.00	0.93, 1.09	.912	1.01	0.90, 1.14	.858	0.96	0.82, 1.13	.660	1.04	0.91, 1.18	.605
	Direct	0.95	0.88, 1.04	.290	0.97	0.85, 1.12	.711	0.96	0.81, 1.14	.669	1.02	0.89, 1.17	.800
Parking difficult [1–4]	Total^a^	1.04	0.95, 1.13	.391	1.03	0.92, 1.17	.588	0.93	0.80, 1.09	.370	1.03	0.90, 1.16	.702
Trees [1–4]	Total	1.04	0.95, 1.14	.364	1.03	0.90, 1.17	.696	0.95	0.80, 1.13	.555	1.02	0.89, 1.18	.746
	Direct	1.03	0.93, 1.13	.613	1.02	0.88, 1.18	.817	0.91	0.75, 1.10	.321	0.99	0.85, 1.17	.940
Distance to school [1–5]	Total^a^	**0.33**	**0.31, 0.35**	**<.001**	**0.56**	**0.50, 0.61**	**<.001**	**0.54**	**0.47, 0.63**	**<.001**	**0.58**	**0.52, 0.64**	**<.001**

^1^excluding transit stops, ^2^excluding parks, *OR* Odd ratio, *CI* Confidence intervals, *p* = *p*-value; in bold: effects significant at *p* < 0.05. ^#^*N* = 3389 instead of 3586 because data from Israel and Portugal were excluded from the analyses due to 0% prevalence of cycling to/from school. Analyses undertaken on 20 imputed datasets. ^a^Total and direct effects are equivalent as no mediating variables of characteristic-outcome associations were included in the models. Complete case analyses are in the Supplementary Material (Tables S24, S25, S26 and S27). Model covariates were based on DAG depicted in Supplementary Materials (Fig S1). All models adjusted for adolescent age, adolescent sex, area-level SES, highest education in the household, number of children, number of adults and city. Additional adjustments per model included:

*Residential density models:* marital status. Direct effect: accessibility and walking facilities; aesthetics; buffers between street and footpath; land use mix – diversity^1^; number of driving license; number of motor vehicles; parking difficult; parks proximity; pedestrian infrastructure and safety; distance to school; recreational facilities^2^; safety from crime; traffic safety; transit stop proximity; trees

*Land use mix diversity models* (*excluding transit stops*): marital status; residential density. Direct effect: accessibility and walking facilities; aesthetics; buffers between street and footpath; number of driving license; number of motor vehicles; parking difficult; parks proximity; pedestrian infrastructure and safety; safety from crime; traffic safety; transit stop proximity; trees

*Transit stop proximity models*: land use mix diversity; residential density. Direct effect: accessibility and walking facilities; aesthetics; buffers between street and footpath; number of driving license; marital status; number of motor vehicles; parks proximity; pedestrian infrastructure and safety; recreational facilities^2^; safety from crime; traffic safety; trees

*Recreational facilities* (*excluding parks*): accessibility and walking facilities; aesthetics; buffers between street and footpath; land use mix diversity; number of driving license; marital status; number of motor vehicles; residential density; parks proximity; pedestrian infrastructure and safety; safety from crime; traffic safety; transit stop proximity; trees

*Park proximity*: land use mix diversity; residential density. Direct effect: accessibility and walking facilities; aesthetics; buffers between street and footpath; pedestrian infrastructure and safety; safety from crime; traffic safety; trees

*Accessibility and walking facilities*: aesthetics; buffers between street and footpath; land use mix diversity; residential density; parks proximity; pedestrian infrastructure and safety; safety from crime; traffic safety; trees; aesthetics; buffers between street and footpath; land use mix diversity; residential density; parks proximity; pedestrian infrastructure and safety; safety from crime; traffic safety; trees

*Traffic safety*: accessibility and walking facilities; aesthetics; buffers between street and footpath; land use mix diversity; residential density; parks proximity; pedestrian infrastructure and safety; safety from crime; trees

*Pedestrians infrastructure and safety*: accessibility and walking facilities; aesthetics; buffers between street and footpath; land use mix diversity; residential density; parks proximity; safety from crime; traffic safety; trees

*Safety from crime*: accessibility and walking facilities; aesthetics; buffers between street and footpath; land use mix diversity; residential density; parks proximity; pedestrian infrastructure and safety; traffic safety; trees

*Aesthetics*: accessibility and walking facilities; buffers between street and footpath; land use mix diversity; residential density; parks proximity; pedestrian infrastructure and safety; safety from crime; traffic safety; trees

*Buffers between street and footpath*: residential density. Direct effect: accessibility and walking facilities; aesthetics; land use mix diversity; parks proximity; pedestrian infrastructure and safety; safety from crime; traffic safety; trees

*Parking difficult*: accessibility and walking facilities; aesthetics; buffers between street and footpath; land use mix diversity; number of driving license; marital status; number of motor vehicles; parks proximity; pedestrian infrastructure and safety; recreational facilities no parks; safety from crime; traffic safety; transit stops; trees

*Trees*: residential density. Direct effect: accessibility and walking facilities; aesthetics; buffers between street and footpath; land use mix diversity; parks proximity; pedestrian infrastructure and safety; safety from crime; traffic safety

*Distance to school*: residential density

### Moderating effects

Moderation of direct effects models according to distance to school, adolescent sex and city is summarised below. Full moderation results for both total and direct effects are available in the Supplementary Materials.

#### Distance

**Fig. 1:**
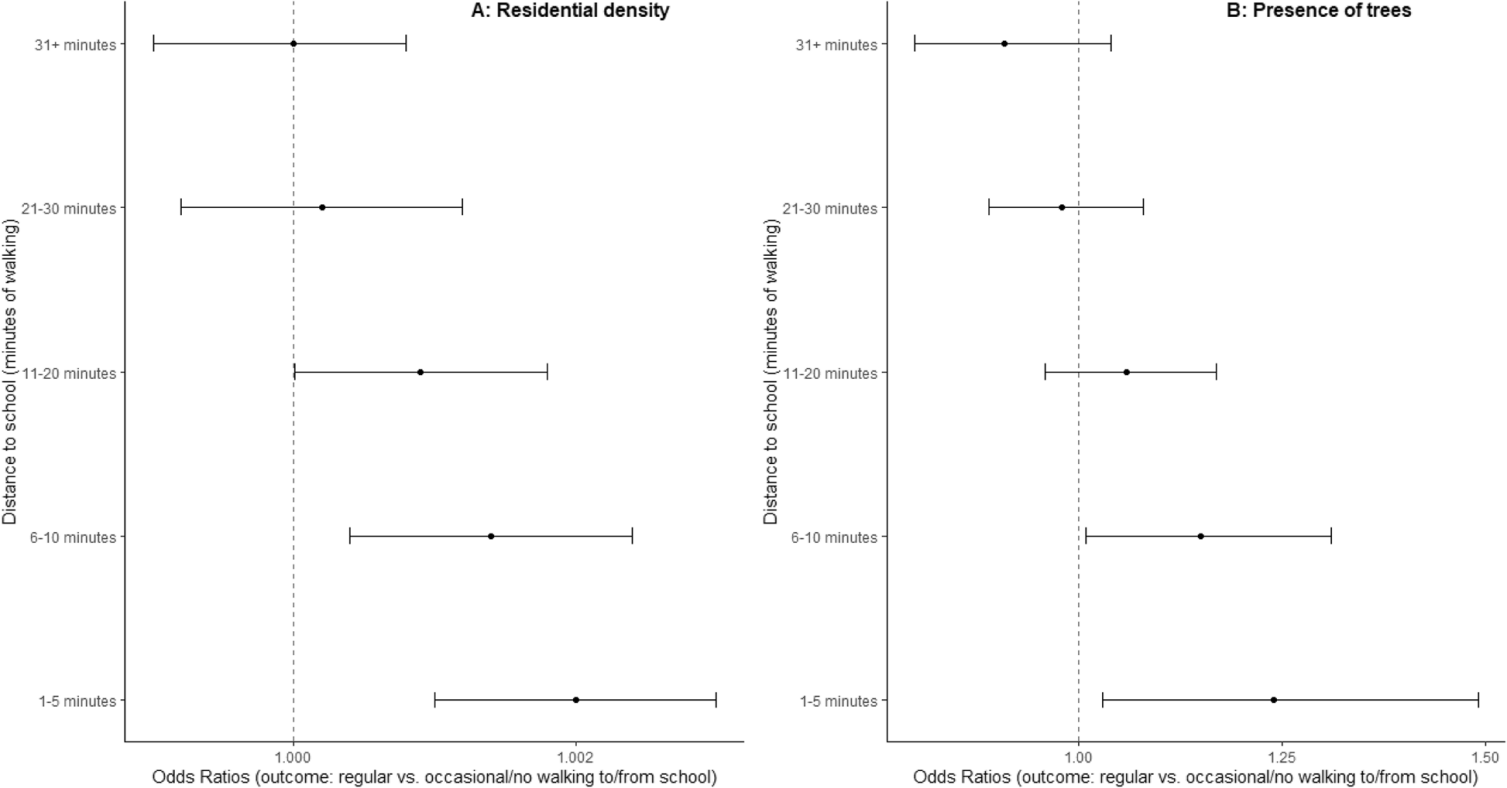
Associations of parent-perceived residential density and presence of trees with adolescents’ regular vs. occasional/no walking to/from school by distance to school (in minutes of walking). Dots represent estimates of odds ratios from direct effect models and whiskers represent 95% confidence intervals

**Fig. 2:**
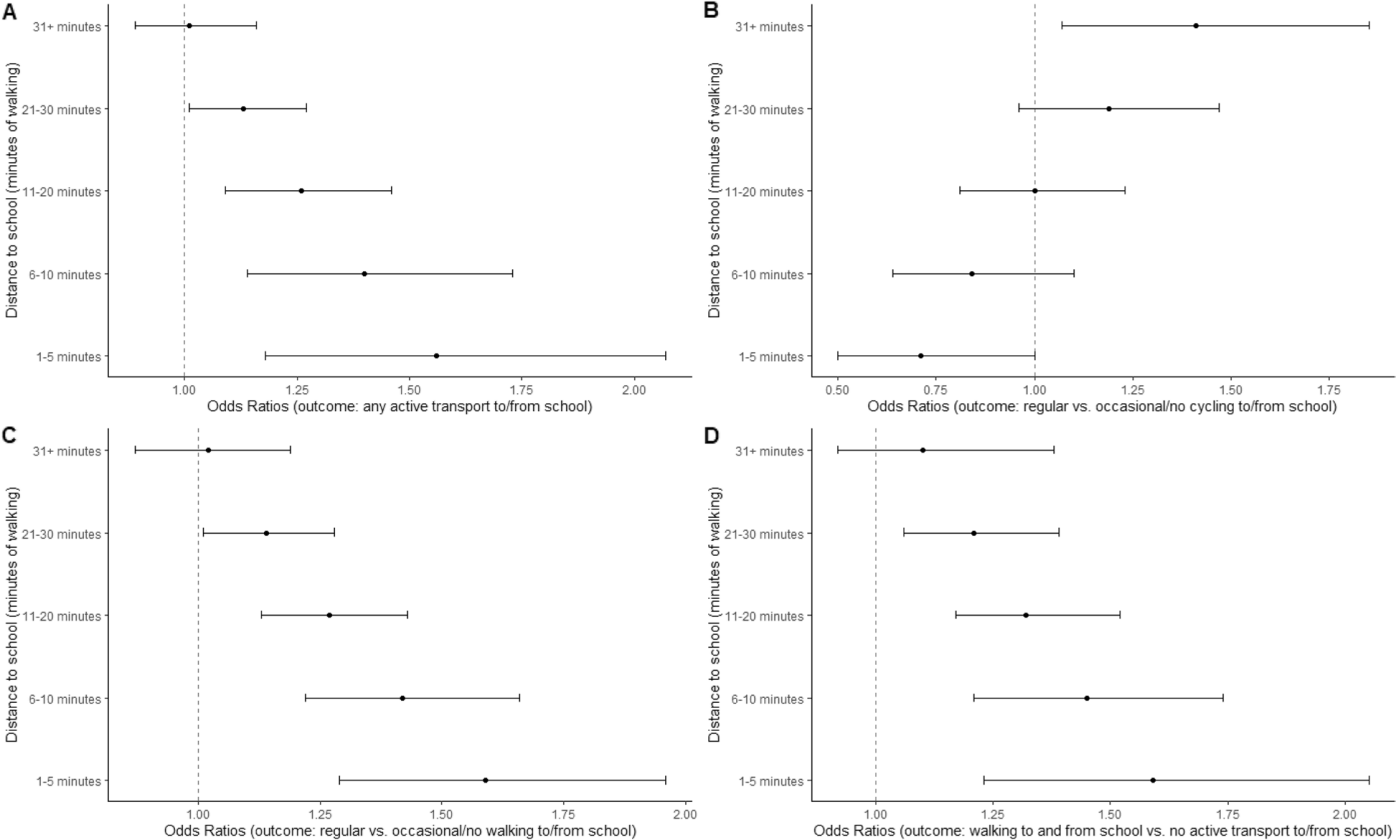
Associations of parent-perceived land use mix – diversity with adolescents’ active transport to/from school by distance to school (in minutes of walking). Dots represent estimates of odds ratios from direct effect models and whiskers represent 95% confidence intervals

**Fig. 3:**
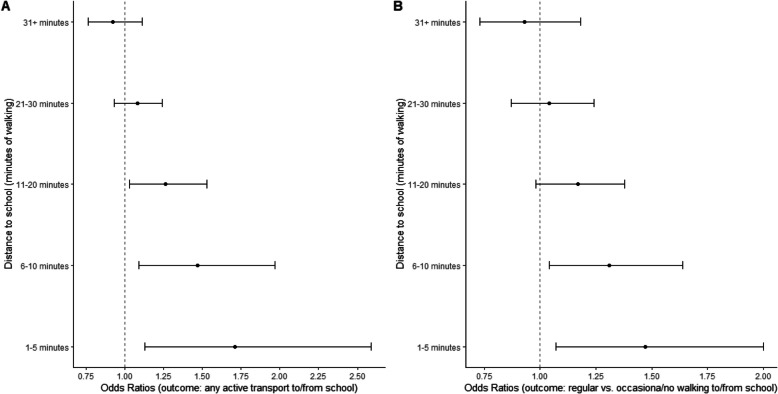
Associations of parent-perceived accessibility and walking facilities with adolescents’ active transport to/from school by distance to school (in minutes of walking). Dots represent estimates of odds ratios from direct effect models and whiskers represent 95% confidence intervals

Distance to school moderated several associations (Tables S8 and S9). Residential density, land use mix – diversity, presence of trees, and accessibility/walking facilities were more strongly positively associated with ATS outcomes at shorter distances to school (Figs. 1, 2 and 3). However land use mix – diversity was negatively related to regular vs. occasional or no cycling to/from school in adolescents whose school was within a 5-min walk from their home (Fig. 2; panel B). Park proximity was negatively related to any ATS and regular vs. occasional or no walking to/from school (Table S10) only if the school was further than a 30-min walk from home, and unrelated to these outcomes for shorter distances to school. Lastly, safety from crime, aesthetics and having buffers between streets and footpaths tended to be positively related to the odds of walking to and from school vs. walking from school only among adolescents who lived further than a 30-min walk from their school (Table S11).

#### Sex

Adolescent’s sex moderated some associations between parent-perceived neighbourhood environment attributes and ATS outcomes (Tables S12 and S13). Positive associations between land use mix – diversity and accessibility/walking facilities, with regular cycling to/from school were found in females, but not males (Table S14). In contrast, park proximity was negatively related to regular cycling in males, but not in females. Males with better access to recreation facilities in the neighbourhood were less likely to regularly cycle to/from school, while for females these effects tended to be in the opposite direction (Table S14). Similar between-sex differences were observed in the effects of park proximity and recreational facilities in relation to the profile cycling to/from school vs. no ATS (Table S15). Finally, parent perceptions of parking being difficult in the neighbourhood tended to be positively associated with the odds of walking to and from school vs. walking from school in females only (Table S16).

#### City

**Fig. 4:**
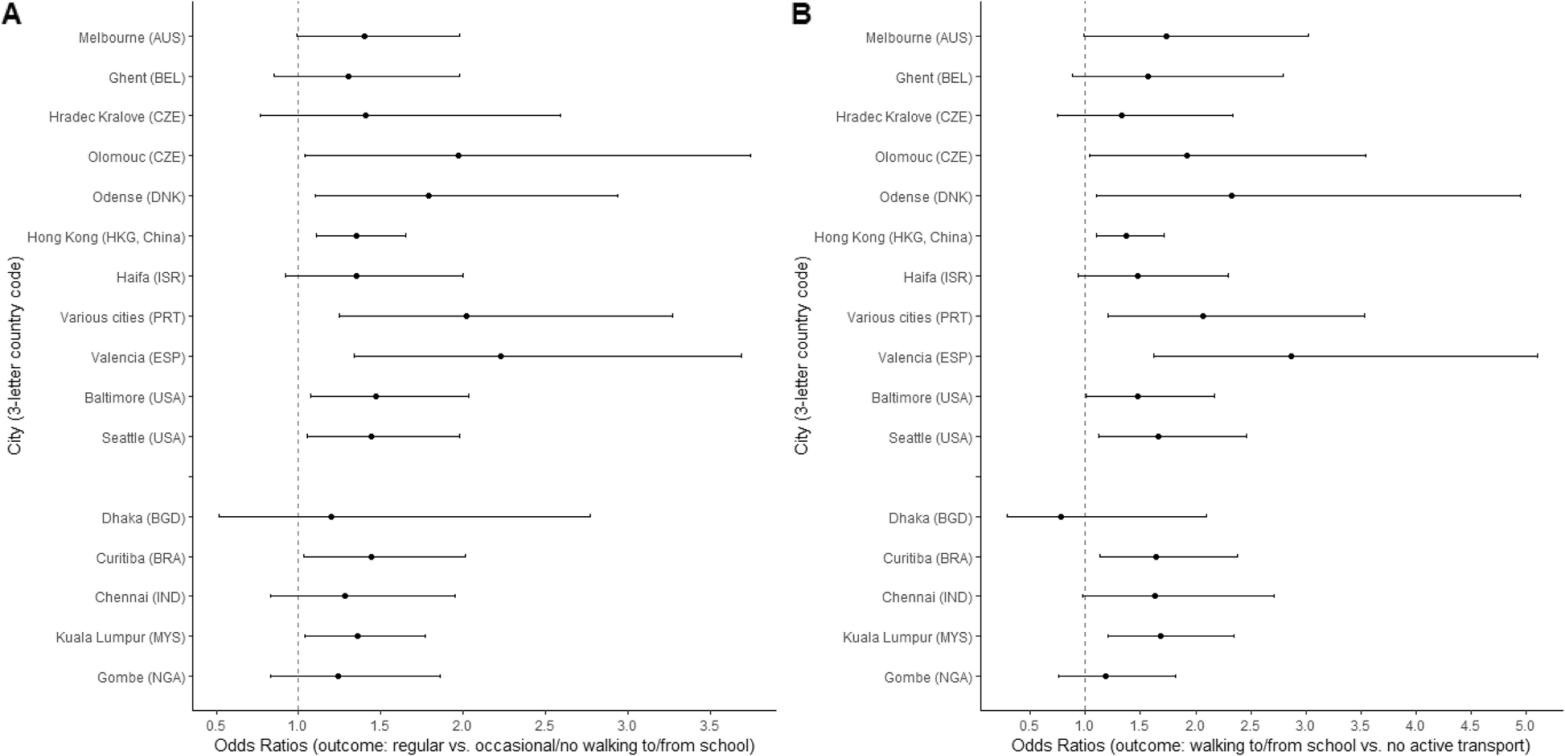
City-specific associations of parent-perceived land use mix – diversity with adolescents’ regular vs. occasional/no walking to/from school (panel A) and walking to and from school vs. no active transport to/from school (panel B). Dots represent estimates of odds ratios from direct effect models and whiskers represent 95% confidence intervals

Associations of a few parent-perceived neighbourhood attributes with cycling to/from school vs. no ATS, and with two walking to/from school outcomes, differed by city (Tables S17 and S18). Whilst higher land use mix – diversity tended to be associated with greater odds of regular vs. occasional or no walking to/from school and walking to and from school vs. no ATS in most cities, the effect sizes (ORs) ranged from 1.20 (Dhaka, Bangladesh) to 2.23 (Valencia, Spain) for the first outcome, and from 0.78 (Dhaka, Bangladesh) to 2.87 (Valencia, Spain) for the second outcome (Fig. 4). Positive associations of access to recreation facilities with walking to and from school vs. no ATS were observed in Odense (Denmark) only (Table S19). Parking being difficult in the neighbourhood was positively related to this ATS outcome only in adolescents from Haifa (Israel) and Baltimore (USA) (Table S19). Study site also moderated the association between parent-perceived distance to school and cycling to/from school vs. no ATS (Table S18). Although the site-specific ORs suggested a negative association between the two variables, the effects were statistically significant in seven out of the 14 cities with a non-zero prevalence of cycling to/from school and ranged from 0.29 (95% CI: 0.17, 0.49; *p* < 0.001) in Odense (Denmark) to 0.94 (95% CI: 0.43, 2.06; *p* = 0.882) in Melbourne (Australia) (Table S20).

### Complete case analyses

Complete case analyses are presented in the Supplementary Materials (Tables S21-S50). There were few substantive differences with analyses based on multiple imputed data. Complete case analyses did not identify any significant environmental correlates of regular cycling to/from school (Table S22), while the models with multiple imputation did (Table 5). Transit stop proximity and safety from crime were negatively associated, and traffic safety and pedestrian infrastructure positively associated, with walking to and from school in complete-case analyses only (Tables 6 and S24). Between-city differences in associations of recreation facilities and distance to school with ATS outcomes were observed in the multiple-imputation, but not complete-case analyses (Tables S48 and S49). Several weak moderating effects of adolescent sex and distance to school on environment-ATS outcome associations were significant in complete-case analyses only (see Supplementary Material).

## Discussion

This heterogeneous, multi-country study found wide variation in active travel to/from school across cities/countries among adolescents and uniquely identified distinct groups of adolescents based on their patterns of travel. The most common profile included those who never or rarely used ATS (almost half of the sample); followed by those who regularly walked both directions (a third), and two smaller profiles comprising those who walked from school but rarely to school (10%) and those who cycled to and from school (8%). Perceived distance to school was the strongest (negative) correlate of all active travel outcomes examined, except regular cycling to/from school. Overall, associations between parent perceptions of the neighbourhood and adolescent active travel differed for walking and for cycling, and there were stronger associations with perceived neighbourhood characteristics among those living closer to school. Indicators of compact, mixed use development such as distance to and diversity of land uses, perceived accessibility/walking facilities, and traffic safety were positively associated with *any* active travel. Diversity of land use and accessibility/walking facilities were associated with more regular walking and the profiles characterised by walking one or both ways. Higher residential density and traffic safety were associated with the profile characterised by walking both to and from school. Regarding cycling, residential density was negatively and aesthetics and safety from crime were positively associated with regular cycling, and aesthetics was positively associated with the profile characterised by cycling to and from school. The differing patterns of environmental correlates of walking and cycling may be due to the longer distances travelled by bike where urban design attributes closer to home may be less important than attributes along the whole route. The lack of behaviour-specific environment attributes for cycling in the NEWS-Y, which includes items more closely aligned with walking than cycling, was an important limitation. In particular, presence and quality of cycling infrastructure were not assessed, though these are known correlates of cycling [38]. Importantly, associations with ATS were generally consistent across cities (though effect sizes varied). There were few perceived environment variables that were related to an ATS outcome in one city only.

Diversity of land uses was a strong positive correlate of ATS outcomes related to walking. Composite measures of land use mix based on broad categories of land use (e.g., commercial, residential, industrial) have consistently been associated with transport-related physical activity among adults [39], including in the multi-country IPEN Adult study which also included some LMICs [40]. There is mixed evidence among adolescents [18]. The land use mix – diversity score used in the current study aggregates access to a range of specific destinations near home, including food stores, eateries, schools, and service-related stores such as post offices and laundries. While some of the service-related destinations are unlikely to be visited frequently by adolescents, the positive associations found suggest that diverse destinations may make it possible for adolescents to engage in other activities en route to school or home, limiting reliance on parents for transport to these types of destinations, particularly after school when there may be fewer time pressures. Areas with a mix of destinations may be more vibrant with more people about, which may increase the desirability of walking compared to areas with fewer destinations [41]. Although locations of friend’s houses were not collected, diversity of destinations may include opportunities to socialise after school that make walking more appealing than direct trips home by car. However, opportunities to socialise after school may only be part of the explanation given that higher land use mix – diversity was associated with 36% higher odds of walking both ways compared to the profile characterised by walking from school only.

Although diversity of destinations was important for walking, associations with cycling outcomes were observed only among girls (positive association) and those living close to school (negative association). The immediate area around schools in destination-rich areas may not provide optimal conditions for cycling due to density of pedestrian and vehicular traffic [44], possible crowding, and potentially unsafe driver behaviour around schools [43], particularly if quality infrastructure to support cycling is lacking (e.g., dedicated cycle lanes, secure bicycle storage at the school). Adolescents may not be confident to cycle in such environments and may prefer to walk rather than cycle short distances. Adolescents in New Zealand, for example, have been shown to have more negative safety perceptions, perceptions of infrastructure (e.g., bike paths) and confidence for cycling compared to walking [19]. These findings highlight the importance of examining environmental correlates of walking and cycling separately and may help explain mixed findings in previous studies [18].

Distance to school is a consistent correlate of transport-related physical activity in children and adolescents [18, 44]. However a novel finding in the present study was the strength of associations between some environmental attributes and ATS depended on distance. For example, associations between walking outcomes and residential density, diversity of land uses, and accessibility/walking facilities were stronger among those living a shorter walk to school. This is perhaps unsurprising given the NEWS-Y is designed to reflect perceived conditions within a 10–15 walk from home. Although not specific to routes to school, NEWS-Y scales had greater context-specificity with the behaviour of interest among those whose school was closer. Exposures to environmental attributes may vary along longer commutes that travel through several areas, and as such the home neighbourhood may not be characteristic of the entire route. Among those living further than a 30-min walk from school, aesthetics and safety-related indicators (safety from crime and buffers between streets and footpaths) were more strongly associated with ATS and walking outcomes compared to those living closer. This finding is consistent with a study in children, which showed that parental concern about dangerous traffic was negatively associated with usually walking and cycling to school among those living 1–2 km away, but not among those living closer [45]. Safety may become a more important consideration as travel distances from home increase due to greater exposure to risk, whereas these considerations may not factor as strongly in decision making when the journey is very short.

Several interactions with sex were identified in this study for cycling, and one interaction was identified for walking. Diversity of destinations, accessibility/walking facilities, and recreation facilities were positively associated with regular cycling among girls, but not boys. It may be that boys (and their parents) were more confident in their ability to cycle, as has been reported in some countries among younger children [46], and that girls require more supportive infrastructure or a need to travel to other destinations beyond school (such as recreation facilities) to enable them to cycle. Women have reported lower confidence and more traffic-related safety concerns related to cycling compared to men [47], and a systematic review found stronger preferences for cycling infrastructure to be separated from traffic among women compared to men [48]. Across the countries represented in the present study, cycling to school was uncommon in all countries except Belgium, Denmark, and India, and previous studies in many countries showed ATS is less common among adolescent girls than boys [14, 49–51]. As noted, the NEWS-Y does not include any items related to cycling-specific infrastructure, such as cycle corridors, protected cycle lanes, or off-road cycling infrastructure. Further, the extent of cycling infrastructure and laws governing cycling (e.g., right of way, ability to ride on footpaths) is likely to vary across cities and countries. Future multi-country studies should incorporate cycling-specific attributes of local neighbourhoods to address this limitation.

The present study had notable strengths. The geographic and socioeconomic heterogeneity of the countries involved in IPEN Adolescent, which included five LMICs, ensured variation in both exposures and behaviours, and these features were intended to enhance power to detect meaningful associations [25]. The variation this approach provided in the pooled analyses enabled associations to be detected. The limited moderation effects identified according to site suggest the reported findings are robust and not driven by a particular country. Such a conclusion is only possible from a multi-country study using comparable methods. Use of common measures allowed these variations to be documented. Wide variation was indeed observed in ATS, with the lowest prevalence in the USA (32%) and the highest in Denmark (91%), though both are high-income countries. Assessing direction of active travel modes and using LPA to identify profiles of ATS were particularly novel. A data-driven, person-centred approach such as LPA allowed underlying patterns of behaviour to be identified from the data rather than relying on arbitrary or conceptually-defined categories of behaviour [20] and enabled more specificity in interpretation and more authentic comparisons. Analytic strengths included assessment of site, distance from school, and sex as moderators, multiple imputation analyses, and supplemental complete-case analyses.

Despite these strengths, the cross-sectional design and reliance on self-reported ATS are limitations, and the results are not generalisable to adolescents living in rural areas. The four latent profiles identified using LPA were based on pooled data; different profiles may have been identified if LPA had been applied within each city/site. Further, given the data-driven nature of LPA and the sampling approach to maximise heterogeneity rather than representativeness within each city/site, other studies may generate different latent profiles. The questions did not allow walking or cycling as part of multi-modal trips to be identified, particularly trips involving public transport, which typically involve walking or cycling [52]. Although proximity to a public transit stop was not associated with any of the ATS outcomes in this study, it is possible the most proximal stop may not provide a direct service or easy access to school. Given that a third of participants lived more than a 30-min walk from school, public transport may be more viable than ATS-only trips, particularly in the absence of safe cycling infrastructure. Future studies should examine neighbourhood correlates of school trips involving public transport, which may have different associations with the neighbourhood environment. We did not adjust analyses for household income. It is possible travel patterns among some participants may be shaped by economic necessity rather than choice [53].

Though widely used to assess neighbourhood perceptions, the NEWS-Y may lack adequate contextual and behaviour-specificity for ATS, given it assesses general perceptions of neighbourhoods rather than route characteristics, does not include items related specifically to school neighbourhoods, and does not include cycling-specific considerations, such as separated bike lanes, continuity of bike lanes, and cycling-related driver behaviour. It is possible parents’ perceptions of the neighbourhood do not adequately match reality, which could impact associations with behaviour [54]. While it is possible adolescents’ perceptions of their neighbourhood are more strongly associated with ATS than parent perceptions as they gain autonomy, the use of parent rather than adolescent-reported perceived neighbourhood avoids common-method bias, given ATS was self-reported. Previous work showed parents remain actively involved in decision making related to mode choice [55], and family support for ATS [56] has been positively associated with ATS during adolescence.

## Conclusions

Promoting active travel is a key strategy for achieving the World Health Organization’s global physical activity targets [57], and ATS has numerous co-benefits for minimising noise, poor air quality and other environmental harms related to motorised transport [58]. The provision of environments that support walking and cycling is critical to realise sustained population-wide improvements, particularly given the large variation in ATS reported across countries in the present study, and that the most common profile consisted of adolescents who never or rarely used ATS. Parent-reported distance to school, diversity in land uses, accessibility/walking facilities, and both traffic and crime-related safety among those living further from school, were important supportive correlates of ATS, particularly walking. Policies that achieve these neighbourhood attributes need to be prioritised to shape neighbourhoods to better support active, environmentally-friendly transport modes. Future research directions include the incorporation of social and economic aspects of the environment, objective measures of the environment attributes related specifically to cycling, as well as built environment correlates of trips involving public transport, which provide opportunities for physical activity as well as sustainable travel, particularly for those living beyond a walkable or cyclable distance.

## Supplementary Information

Supplementary Material 1.

## Data Availability

The datasets used and/or analysed during the current study are available on reasonable request.

## Abbreviations

ATS: Active School Travel
HICs: High-income countries
IPEN: International Physical activity and Environment Network
GAMMs: Generalised Additive Mixed Models
LMICs: Low-to-middle-income countries
LPA: Latent Profile Analyses
NEWS-Y: Neighbourhood Environment Walkability Scale for Youth
SES: Socioeconomic Status
