# Supplementary material for: Associations between parental perceptions of neighbourhood environments and active travel to school: IPEN Adolescent study
Source: Int J Behav Nutr Phys Act. 2025 May 15;22:55. doi: 10.1186/s12966-025-01738-3 (PMC12079927; doi:10.1186/s12966-025-01738-3)
Supplement: Supplementary file 1 — Supplementary Material 1. [file 12966_2025_1738_MOESM1_ESM.docx]

**Supplementary Material**

**Associations between parental perceptions of neighbourhood environments and active travel to school: IPEN Adolescent Study**

*Authors: Anna Timperio, Scott Duncan, Muhammad Akram, Javier Molina-García, Delfien Van Dyck, Anthony Barnett, Ferdinand Salonna, Anjana RM, James F Sallis, Michal Vorlíček, Erica Hinckson, Kelli L. Cain, Terry L. Conway, Wan Abdul Manan Wan Muda, Mika Moran, Adewale L Oyeyemi, Andreia Pizarro, Rodrigo S Reis, Shiekh Muhammad Rezwan, Jasper Schipperijn, Ester Cerin*

*Supplementary files prepared by: Ester Cerin, Muhammad Akram, Anthony Barnett*

**Contents**

[**Figure S1.** Directed acyclic graph (DAG) 5](#_Toc195539510)

[**Table S1.** Covariates of regression models of perceived neighbourhood environment characteristics (exposures) and adolescents’ active transport to/from school (outcome) 6](#_Toc195539511)

[**Table S2.** Overall and site-specific socio-demographic characteristics (imputed data) 9](#_Toc195539512)

[**Table S3.** Overall and site-specific outcome variables: active transport to/from school (imputed data) 10](#_Toc195539513)

[**Table S4.** Overall and site-specific perceived neighbourhood environment attributes (imputed data) 11](#_Toc195539514)

[**LATENT PROFILE ANALYSES OF ADOLESCENTS’ ACTIVE TRANSPORT TO/FROM SCHOOL** 13](#_Toc195539515)

[**Table S5.** Fit indices for various latent profile analysis models 14](#_Toc195539516)

[**TOTAL-EFFECT MODELS** 15](#_Toc195539517)

[**Table S6.** Total effects of parent-perceived neighbourhood environment characteristics on adolescents’ active transport to/from school (multiple imputations) 15](#_Toc195539518)

[**Table S7.** Total effects of parent-perceived neighbourhood environment characteristics on latent profiles of adolescents’ active transport to/from school (multiple imputations) 16](#_Toc195539519)

[**MODERATION ANALYSES: Distance to school** 17](#_Toc195539520)

[**Table S8.** Distance to school as a moderator of total and direct effects of perceived neighbourhood environment characteristics on adolescents’ active transport to/from school (multiple imputations; N=6302) 17](#_Toc195539521)

[**Table S9.** Distance to school as a moderator of total and direct effects of perceived neighbourhood environment characteristics on latent profiles of adolescents’ active transport to/from school (multiple imputations) 18](#_Toc195539522)

[**Table S10.** Total and direct effects of parent-perceived park proximity on adolescents’ any vs. no active transport to/from school and regular vs. occasional/no walking to/from school by distance to school (multiple imputations; N = 6302) 19](#_Toc195539523)

[**Table S11.** Total and direct effects of parent-perceived neighbourhood environment characteristics on adolescents’ walking to and from school vs. walking from school by distance to school (multiple imputations; N = 2708) 20](#_Toc195539524)

[**MODERATION ANALYSES: Adolescent’s sex** 21](#_Toc195539525)

[**Table S12.** Adolescents’ sex as a moderator of total and direct effects of perceived neighbourhood environment characteristics on adolescents’ active transport to/from school (multiple imputations; N=6302) 21](#_Toc195539526)

[**Table S13.** Adolescents’ sex as a moderator of total and direct effects of perceived neighbourhood environment characteristics on latent profiles of adolescents’ active transport to/from school (multiple imputations) 22](#_Toc195539527)

[**Table S14.** Sex-specific effects of land use mix-diversity, neighbourhood accessibility and walking facilities, park proximity and recreation facilities on regular cycling to/from school vs. no or less frequent cycling to/from school (multiple imputations; N = 5703^#^) 23](#_Toc195539528)

[**Table S15.** Sex-specific effects of park proximity and recreational facilities on cycling to and from school vs. no active transport to/from school (multiple imputations; N = 3389^#^) 23](#_Toc195539529)

[**Table S16.** Sex-specific effects of parking being difficult on regular walking to/from school vs. walking from school (multiple imputations; N = 2708) 24](#_Toc195539530)

[**MODERATION ANALYSES: City** 25](#_Toc195539531)

[**Table S17.** Difference in Akaike Information Criterion (AIC) values between models with and without city as a moderator of total and direct effects of perceived neighbourhood environment characteristics on adolescents’ active transport to/from school (multiple imputations; N=6302) 25](#_Toc195539532)

[**Table S18.** Difference in Akaike Information Criterion (AIC) values between models with and without city as a moderator of total and direct effects of perceived neighbourhood environment characteristics on latent profiles of adolescents’ active transport to/from school (multiple imputations) 26](#_Toc195539533)

[**Table S19.** City-specific total and direct effects of parent-perceived neighbourhood recreational facilities and parking on adolescents’ walking to and from school vs. no active transport to/from school (reference category) (2 latent profiles) (multiple imputations; n=5213) 27](#_Toc195539534)

[**Table S20.** City-specific total and direct effects of parent-perceived distance to school on adolescents’ cycling to/from school vs. no active transport to/from school (reference category) (2 latent profiles) (multiple imputations; n=3389)^#^ 28](#_Toc195539535)

[**COMPLETE CASE ANALYSES** 29](#_Toc195539536)

[**Table S21.** Total and direct effects of perceived neighbourhood environment characteristics on adolescents’ active transport to/from school [complete case analyses; N=4725] 29](#_Toc195539537)

[**Table S22.** Total and direct effects of perceived neighbourhood environment characteristics on adolescents’ regular cycling to/from school [complete case analyses; N= 4355] (excluding Israel*, Portugal* and Czech Rep-Olomouc)] 31](#_Toc195539538)

[**Table S23.** Total and direct effects of perceived neighbourhood environment characteristics on adolescents’ regular walking to/from school [complete case analyses; N=4725] 32](#_Toc195539539)

[**Table S24.** Total and direct effects of perceived neighbourhood environment characteristics on adolescents’ walking to and from school vs. no active transport to/from school (reference category) (2 latent profiles) [complete case analyses; N=4960] 33](#_Toc195539540)

[**Table S25.** Total and direct effects of perceived neighbourhood environment characteristics on adolescents’ walking from school vs. no active transport to/from school (reference category) (2 latent profiles) [complete case analyses; N=3518] 34](#_Toc195539541)

[**Table S26.** Total and direct effects of perceived neighbourhood environment characteristics on adolescents’ cycling to and from school vs. no active transport to/from school (reference category) (2 latent classes) [complete case analyses; N=3389] 35](#_Toc195539542)

[**Table S27.** Total and direct effects of perceived neighbourhood environment characteristics on adolescents’ walking to and from school vs. walking from school (reference category) (2 latent profiles) [complete case analyses; N=2604] 36](#_Toc195539543)

[**Table S28.** Distance to school as a moderator of total and direct effects of perceived neighbourhood environment characteristics on adolescents’ active transport to/from school [complete case analyses] 37](#_Toc195539544)

[**Table S29.** Distance to school as a moderator of total and direct effects of perceived neighbourhood environment characteristics on adolescents’ regular cycling to/from school (excluding Israel, Portugal and Czech Rep-Olomouc) [complete case analyses] 40](#_Toc195539545)

[**Table S30.** Distance to school as a moderator of total and direct effects of perceived neighbourhood environment characteristics on adolescents’ regular walking to/from school [complete case analyses] 42](#_Toc195539546)

[**Table S31.** Distance to school as a moderator of total and direct effects of perceived neighbourhood environment characteristics on adolescents’ walking to and from school vs. no active transport to from school (reference category) (2 latent profiles) [complete case analyses] 45](#_Toc195539547)

[**Table S32.** Distance to school as a moderator of total and direct effects of perceived neighbourhood environment characteristics on adolescents’ walking from school vs. no active transport to/from school (reference category) (2 latent profiles) [complete case analyses] 47](#_Toc195539548)

[**Table S33.** Proximity to school as a moderator of total and direct effects of perceived neighbourhood environment characteristics on adolescents’ cycling to and from school vs. no active transport to/from school (reference category) (2 latent classes) [complete case analyses] 49](#_Toc195539549)

[**Table S34.** Distance to school as a moderator of total and direct effects of perceived neighbourhood environment characteristics on adolescents’ walking to and from school vs. walking from school (reference category) (2 latent profiles) [complete case analyses] 50](#_Toc195539550)

[**Table S35.** Adolescents’ sex as a moderator of total and direct effects of perceived neighbourhood environment characteristics on adolescents’ active transport to/from school [complete case analyses] 52](#_Toc195539551)

[**Table S36.** Adolescents’ sex as a moderator of total and direct effects of perceived neighbourhood environment characteristics on adolescents’ regular cycling to/from school (excluding Israel, Portugal and Czech Rep-Olomouc) [complete case analyses] 53](#_Toc195539552)

[**Table S37.** Adolescents’ sex as a moderator of total and direct effects of perceived neighbourhood environment characteristics on adolescents’ regular walking to/from school [complete case analyses] 55](#_Toc195539553)

[**Table S38.** Adolescents’ sex as a moderator of total and direct effects of perceived neighbourhood environment characteristics on adolescents’ walking to and from school vs. no active transport to/from school (reference category) (2 latent profiles) [complete case analyses] 56](#_Toc195539554)

[**Table S39.** Adolescents’ sex as a moderator of total and direct effects of perceived neighbourhood environment characteristics on adolescents’ walking from school vs. no active transport to/from school (reference category) (2 latent profiles) [complete case analyses] 57](#_Toc195539555)

[**Table S40.** Adolescents’ sex as a moderator of total and direct effects of perceived neighbourhood environment characteristics on adolescents’ cycling to and from school vs. no active transport to/from school (reference category) (2 latent classes) [complete case analyses] 58](#_Toc195539556)

[**Table S41.** Adolescents’ sex as a moderator of total and direct effects of perceived neighbourhood environment characteristics on adolescents’ walking to and from school vs. walking from school (reference category). (2 latent profiles) 60](#_Toc195539557)

[**Table S42.** Difference in Akaike Information Criterion (AIC) values between models with and without city as a moderator of total/direct effects of perceived environment characteristics on adolescents’ active transport to/from school 62](#_Toc195539558)

[**Table S43.** Difference in Akaike Information Criterion (AIC) values between models with and without city as a moderator of total/direct effects of perceived environment characteristics on adolescents’ regular cycling to/from school (excluding Israel, Portugal and Czech Rep-Olomouc) 63](#_Toc195539559)

[**Table S44.** Difference in Akaike Information Criterion (AIC) values between models with and without city as a moderator of total/direct effects of perceived neighbourhood environment characteristics on adolescents’ regular walking to/from school 64](#_Toc195539560)

[**Table S45.** City as a moderator of the direct effects of perceived neighbourhood environment characteristics on adolescents’ regular walking to/from school 65](#_Toc195539561)

[**Table S46.** Difference in Akaike Information Criterion (AIC) values between models with and without city as a moderator of total/direct effects of perceived neighbourhood environment characteristics on adolescents’ walking to and from school vs. no active transport to/from school (reference category) (2 latent profiles) 66](#_Toc195539562)

[**Table S47.** City as a moderator of direct effects of perceived neighbourhood environment characteristics on adolescents’ walking to and from school vs. no active transport to/from school (reference category) (2 latent profiles) 67](#_Toc195539563)

[**Table S48.** Difference in Akaike Information Criterion (AIC) values between models with and without city as a moderator of total/direct effects of perceived environment characteristics on adolescents’ walking from school vs. no active transport to/from school (reference category) (2 latent classes) 68](#_Toc195539564)

[**Table S49.** Difference in Akaike Information Criterion (AIC) values between models with and without city as a moderator of total/direct effects of perceived environment characteristics on adolescents’ cycling to/from school vs. no active transport to/from school (reference category) (2 latent classes) 69](#_Toc195539565)

[**Table S50.** Difference in Akaike Information Criterion (AIC) values between models with and without city as a moderator of total/direct effects of perceived neighbourhood environment characteristics on adolescents’ walking to and from school vs. walking from school (reference category) (2 latent profiles) 70](#_Toc195539566)

**
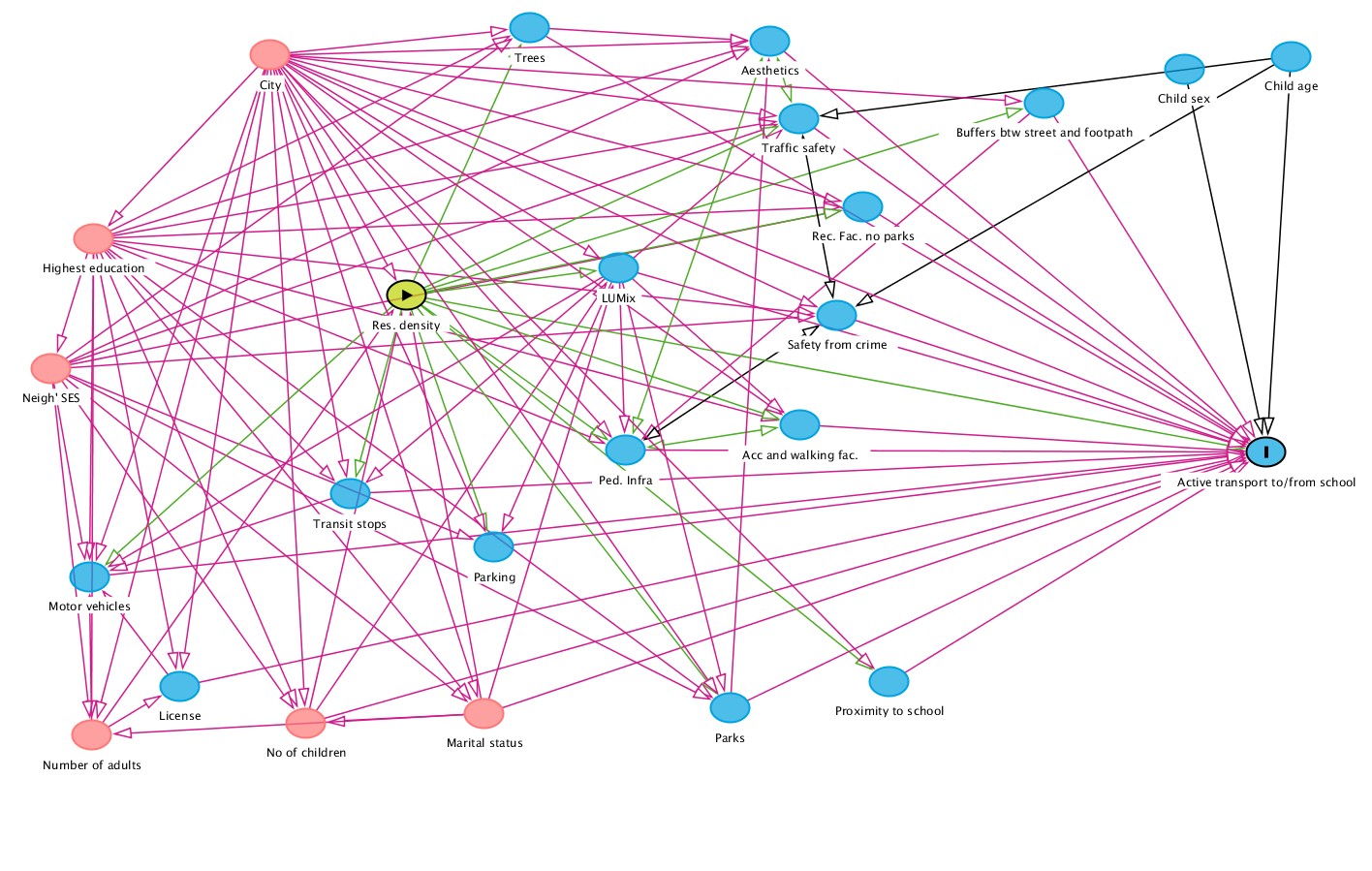
**

**Figure S1.** Directed acyclic graph (DAG)
Showing hypothesised relations between characteristics of the neighbourhood environments with adolescents’ active transport to/from school. Covariates to be included in the statistical analyses were chosen based on the DAG to sufficiently control for potential confounders. The DAG depicted here informed the model of the total effect of parental perceptions of residential density on adolescents’ active transport to/from school. Red circles denote potential confounders. A subset of these variables were included in the regression models as the minimal sufficient set of confounders.

# Table S1. Covariates of regression models of perceived neighbourhood environment characteristics (exposures) and adolescents’ active transport to/from school (outcome)

| **Model** | **Effect estimated** | **Covariates** |
| --- | --- | --- |
| 1T | Total effects of Residential density | adolescent age; adolescent sex; number of children; number of adults; area-level SES; highest education in household; city; marital status |
| 1D | Direct effects of Residential density | adolescent age; adolescent sex; area-level SES; highest education in household; city; number of children; number of adults; accessibility and walking facilities; aesthetics; buffers between street and footpath; land use mix – diversity^1^; number of driving license; marital status; number of motor vehicles; parking difficult; parks proximity; pedestrian infrastructure and safety; distance to school; recreational facilities^2^; safety from crime; traffic safety; transit stop proximity; trees |
| 2T | Total effects of Land use mix diversity^1^ | adolescent age; adolescent sex; number of children; number of adults; area-level SES; highest education in household; city; marital status; residential density |
| 2D | Direct effects of Land use mix diversity^1^ | adolescent age; adolescent sex; area-level SES; highest education in household; city; number of children; number of adults; accessibility and walking facilities; aesthetics; buffers between street and footpath; number of driving license; marital status; number of motor vehicles; residential density; parking difficult; parks proximity; pedestrian infrastructure and safety; safety from crime; traffic safety; transit stop proximity; trees |
| 3T | Total effects of Transit stop proximity | adolescent age; adolescent sex; number of children; number of adults; area-level SES; highest education in household; city; land use mix diversity; residential density |
| 3D | Direct effects of Transit stop proximity | adolescent age; adolescent sex; number of children; number of adults; area-level SES; highest education in household; city; accessibility and walking facilities; aesthetics; buffers between street and footpath; land use mix diversity; number of driving license; marital status; number of motor vehicles; residential density; parks proximity; pedestrian infrastructure and safety; recreational facilities^2^; safety from crime; traffic safety; trees |
| 4T | Total effects of Recreational facilities^2^ | adolescent age; adolescent sex; number of children; number of adults; area-level SES; highest education in household; city; accessibility and walking facilities; aesthetics; buffers between street and footpath; land use mix diversity; number of driving license; marital status; number of motor vehicles; residential density; parks proximity; pedestrian infrastructure and safety; safety from crime; traffic safety; transit stop proximity; trees |
| 4D | Direct effects of Recreational facilities^2^ | adolescent age; adolescent sex; number of children; number of adults; area-level SES; highest education in household; city; accessibility and walking facilities; aesthetics; buffers between street and footpath; land use mix diversity; number of driving license; marital status; number of motor vehicles; residential density; parks proximity; pedestrian infrastructure and safety; safety from crime; traffic safety; transit stop proximity; trees |
| 5T | Total effects of Park proximity | adolescent age; adolescent sex; number of children; number of adults; area-level SES; highest education in household; city; land use mix diversity; residential density |
| 5D | Direct effects of Park proximity | adolescent age; adolescent sex; number of children; number of adults; area-level SES; highest education in household; city; accessibility and walking facilities; aesthetics; buffers between street and footpath; land use mix diversity; residential density; pedestrian infrastructure and safety; safety from crime; traffic safety; trees |
| 6T | Total effects of Accessibility and walking facilities | adolescent age; adolescent sex; number of children; number of adults; area-level SES; highest education in household; city; aesthetics; buffers between street and footpath; land use mix diversity; residential density; parks proximity; pedestrian infrastructure and safety; safety from crime; traffic safety; trees |
| 6D | Direct effects of Accessibility and walking facilities | adolescent age; adolescent sex; number of children; number of adults; area-level SES; highest education in household; city; aesthetics; buffers between street and footpath; land use mix diversity; residential density; parks proximity; pedestrian infrastructure and safety; safety from crime; traffic safety; trees |
| 7T | Total effects of Traffic safety | adolescent age; adolescent sex; number of children; number of adults; area-level SES; highest education in household; city; accessibility and walking facilities; aesthetics; buffers between street and footpath; land use mix diversity; residential density; parks proximity; pedestrian infrastructure and safety; safety from crime; trees |
| 7D | Direct effects of Traffic safety | adolescent age; adolescent sex; number of children; number of adults; area-level SES; highest education in household; city; accessibility and walking facilities; aesthetics; buffers between street and footpath; land use mix diversity; residential density; parks proximity; pedestrian infrastructure and safety; safety from crime; trees |
| 8T | Total effects of Pedestrians infrastructure and safety | adolescent age; adolescent sex; number of children; number of adults; area-level SES; highest education in household; city; accessibility and walking facilities; aesthetics; buffers between street and footpath; land use mix diversity; residential density; parks proximity; safety from crime; traffic safety; trees |
| 8D | Direct effects of Pedestrians infrastructure and safety | adolescent age; adolescent sex; number of children; number of adults; area-level SES; highest education in household; city; accessibility and walking facilities; aesthetics; buffers between street and footpath; land use mix diversity; residential density; parks proximity; safety from crime; traffic safety; trees |
| 9T | Total effects of Safety from crime | adolescent age; adolescent sex; number of children; number of adults; area-level SES; highest education in household; city; accessibility and walking facilities; aesthetics; buffers between street and footpath; land use mix diversity; residential density; parks proximity; pedestrian infrastructure and safety; traffic safety; trees |
| 9D | Direct effects of Safety from crime | adolescent age; adolescent sex; number of children; number of adults; area-level SES; highest education in household; city; accessibility and walking facilities; aesthetics; buffers between street and footpath; land use mix diversity; residential density; parks proximity; pedestrian infrastructure and safety; traffic safety; trees |
| 10T | Total effects of Aesthetics | adolescent age; adolescent sex; number of children; number of adults; area-level SES; highest education in household; city; accessibility and walking facilities; buffers between street and footpath; land use mix diversity; residential density; parks proximity; pedestrian infrastructure and safety; safety from crime; traffic safety; trees |
| 10D | Direct effects of Aesthetics | adolescent age; adolescent sex; number of children; number of adults; area-level SES; highest education in household; city; accessibility and walking facilities; buffers between street and footpath; land use mix diversity; residential density; parks proximity; pedestrian infrastructure and safety; safety from crime; traffic safety; trees |
| 11T | Total effects of Buffers between street and footpath | adolescent age; adolescent sex; number of children; number of adults; area-level SES; highest education in household; city; residential density |
| 11D | Direct effects of Buffers between street and footpath | adolescent age; adolescent sex; number of children; number of adults; area-level SES; highest education in household; city; accessibility and walking facilities; aesthetics; land use mix diversity; residential density; parks proximity; pedestrian infrastructure and safety; safety from crime; traffic safety; trees |
| 12T | Total effects of Parking difficult | adolescent age; adolescent sex; number of children; number of adults; area-level SES; highest education in household; city; accessibility and walking facilities; aesthetics; buffers between street and footpath; land use mix diversity; number of driving license; marital status; number of motor vehicles; residential density; parks proximity; pedestrian infrastructure and safety; recreational facilities no parks; safety from crime; traffic safety; transit stops; trees |
| 12D | Direct effects of Parking difficult | adolescent age; adolescent sex; number of children; number of adults; area-level SES; highest education in household; city; accessibility and walking facilities; aesthetics; buffers between street and footpath; land use mix diversity; number of driving license; marital status; number of motor vehicles; residential density; parks proximity; pedestrian infrastructure and safety; recreational facilities no parks; safety from crime; traffic safety; transit stops; trees |
| 13T | Total effects of Trees | adolescent age; adolescent sex; number of children; number of adults; area-level SES; highest education in household; city; residential density |
| 13D | Direct effects of Trees | adolescent age; adolescent sex; number of children; number of adults; area-level SES; highest education in household; city; accessibility and walking facilities; aesthetics; buffers between street and footpath; land use mix diversity; residential density; parks proximity; pedestrian infrastructure and safety; safety from crime; traffic safety |
| 14T | Total effects of Distance to school | adolescent age; adolescent sex; number of children; number of adults; area-level SES; highest education in household; city; residential density |
| 14D | Direct effects of Distance to school | adolescent age; adolescent sex; number of children; number of adults; area-level SES; highest education in household; city; residential density |

*Notes.* ^1^ excluding transit stops; ^2^ excluding parks; SES, socio-economic status

# **Table S2.** Overall and site-specific socio-demographic characteristics (imputed data)

|  |  | **High-income countries** | | | | | | | | | | | **Low-middle-income countries** | | | | |
| --- | --- | --- | --- | --- | --- | --- | --- | --- | --- | --- | --- | --- | --- | --- | --- | --- | --- |
|  | **All sites** | **Australia Melb** | **Belgium Ghent** | **Czech Rep HK** | **Czech Rep Olomouc** | **Denmark Odense** | **China Hong Kong** | **Israel Haifa** | **Portugal Various cities** | **Spain Valencia** | **USA Baltimore** | **USA Seattle** | **Bangladesh Dhaka** | **Brazil Curitiba** | **India Chennai** | **Malaysia KL** | **Nigeria Gombe** |
| **N** | 6302 | 438 | 291 | 155 | 183 | 210 | 1295 | 232 | 184 | 465 | 485 | 443 | 92 | 493 | 316 | 752 | 268 |
| **Child’s age (year)** | |  |  |  |  |  |  |  |  |  |  |  |  |  |  |  |  |
| Mean  (SD) | 14.46 (1.69) | 14.91  (1.59) | 13.36  (1.36) | 14.32  (1.73) | 13.73  (1.61) | 13.00  (1.19) | 14.31  (1.70) | 15.28  (1.44) | 15.94  (1.17) | 16.56  (0.78) | 14.15  (1.40) | 14.04  (1.40) | 13.89  (1.76) | 14.08  (1.63) | 13.75  (1.53) | 14.40  (1.32) | 15.26  (1.64) |
| **Child’s sex** |  |  |  |  |  |  |  |  |  |  |  |  |  |  |  |  |  |
| % male | 46.46 | 40.18 | 41.92 | 50.32 | 44.81 | 40.95 | 42.78 | 39.22 | 37.50 | 44.95 | 46.60 | 52.82 | 53.26 | 48.88 | 52.53 | 53.06 | 54.48 |
| **Highest education in the household** | | |  |  |  |  |  |  |  |  |  |  |  |  |  |  |  |
| % College or higher | 52.18 | 63.13 | 74.55 | 53.55 | 36.48 | 69.07 | 35.29 | 61.62 | 42.88 | 55.70 | 74.49 | 76.23 | 58.70 | 40.77 | 47.28 | 41.18 | 55.75 |
| **Area-level SES** | |  |  |  |  |  |  |  |  |  |  |  |  |  |  |  |  |
| % High | 47.11 | 45.89 | 51.89 | 49.68 | 44.26 | 53.81 | 46.49 | 50.43 | 52.72 | 53.55 | 50.93 | 49.21 | 44.57 | 43.20 | 48.10 | 39.89 | 41.04 |
| **Parental marital status** | |  |  |  |  |  |  |  |  |  |  |  |  |  |  |  |  |
| % married/ LWP | 84.92 | 81.75 | 85.48 | 80.65 | 82.43 | 84.24 | 89.65 | 78.81 | 81.58 | 78.71 | 80.54 | 87.78 | 95.65 | 72.01 | 95.57 | 89.95 | 86.19 |
| **Household size** | |  |  |  |  |  |  |  |  |  |  |  |  |  |  |  |  |
| Mean  (SD) | 4.65  (2.61) | 4.35  (1.83) | 4.32  (1.19) | 3.88  (1.23) | 3.90  (1.08) | 4.25  (1.35) | 4.16  (1.16) | 4.34  (1.16) | 3.91  (1.49) | 3.77  (0.86) | 4.31  (1.45) | 4.22  (1.22) | 5.88  (3.99) | 4.26  (1.31) | 4.56  (1.75) | 5.20  (2.27) | 11.71  (7.12) |
| **Children in the household** | | |  |  |  |  |  |  |  |  |  |  |  |  |  |  |  |
| Mean  (SD) | 2.09  (1.55) | 1.99  (0.94) | 2.25  (1.24) | 1.69  (0.74) | 1.77  (0.71) | 2.21  (1.11) | 1.66  (0.75) | 1.96  (0.97) | 1.50  (0.93) | 1.45  (0.58) | 2.08  (1.21) | 1.97  (0.96) | 2.37  (1.66) | 1.85  (0.99) | 1.81  (0.70) | 2.49  (1.41) | 5.98  (4.05) |
| **Motor vehicle in the household** | | |  |  |  |  |  |  |  |  |  |  |  |  |  |  |  |
| % 0  % 1  % 2  % 3+ | 21.41  30.54  30.39  17.66 | 3.70  28.01  44.58  23.71 | 4.97  39.21  43.20  12.63 | 10.06  49.65  32.42  7.87 | 12.43  51.89  27.35  8.33 | 7.95  49.76  36.36  5.93 | 69.19  22.86  6.18  1.78 | 11.31  42.46  38.36  7.87 | 7.39  33.21  47.50  11.90 | 7.31  45.59  33.12  13.98 | 2.11  16.04  46.41  35.43 | 0.90  9.27  43.01  46.82 | 68.48  25.00  1.09  5.43 | 18.86  41.99  28.40  10.75 | 24.53  49.15  18.97  7.36 | 2.37  19.87  41.51  36.25 | 10.50  33.68  29.16  26.66 |
| **Licensed driver in the household** | | |  |  |  |  |  |  |  |  |  |  |  |  |  |  |  |
| % 0  % 1  % 2  % 3+ | 14.03  25.64  44.33  16.00 | 1.31  19.38  57.24  22.07 | 1.80  19.24  73.85  5.10 | 2.52  22.45  56.77  18.26 | 5.41  29.02  51.17  14.40 | 3.50  22.05  64.83  9.62 | 39.46  32.28  26.25  2.01 | 6.47  25.54  42.93  25.06 | 3.18  20.43  59.84  16.55 | 3.23  26.02  51.18  19.57 | 1.26  11.95  57.96  28.84 | 0.23  7.00  60.68  32.10 | 67.01  26.36  1.20  5.43 | 16.43  30.22  38.95  14.40 | 22.55  56.23  18.02  3.20 | 1.24  19.30  50.00  29.47 | 27.95  44.78  17.29  9.98 |

*Notes.* KL=Kuala Lumpur; Melb=Melbourne; Rep=Republic; HK=Hradec Kralove; SES=Socio-economic-status; SD=standard deviation; LWP=living with partner.

# **Table S3.** Overall and site-specific outcome variables: active transport to/from school (imputed data)

|  |  | **High-income countries** | | | | | | | | | | | **Low-middle-income countries** | | | | |
| --- | --- | --- | --- | --- | --- | --- | --- | --- | --- | --- | --- | --- | --- | --- | --- | --- | --- |
|  | **All sites** | **Australia Melb** | **Belgium Ghent** | **Czech Rep HK** | **Czech Rep Olomouc** | **Denmark Odense** | **China Hong Kong** | **Israel Haifa** | **Portugal Various cities** | **Spain Valencia** | **USA Baltimore** | **USA Seattle** | **Bangladesh Dhaka** | **Brazil Curitiba** | **India Chennai** | **Malaysia KL** | **Nigeria Gombe** |
| **N** | 6302 | 438 | 291 | 155 | 183 | 210 | 1295 | 232 | 184 | 465 | 485 | 443 | 92 | 493 | 316 | 752 | 268 |
| **Walk to school (times/week)** | | |  |  |  |  |  |  |  |  |  |  |  |  |  |  |  |
| Mean  (SD) | 1.74 (2.28) | 0.56  (1.40) | 1.11  (1.96) | 2.73  (2.39) | 2.97  (2.37) | 0.85  (1.64) | 2.16  (2.39) | 1.58  (2.11) | 2.28  (2.34) | 4.00  (1.87) | 0.57  (1.44) | 0.77 (1.65) | 2.90  (2.25) | 2.39  (2.44) | 1.23  (2.12) | 1.04  (1.89) | 2.16  (2.38) |
| **Cycle to school (times/week)** | | |  |  |  |  |  |  |  |  |  |  |  |  |  |  |  |
| Mean  (SD) | 0.39 (1.26) | 0.09  (0.57) | 2.45  (2.35) | 0.34  (1.09) | 0.16  (0.76) | 3.49  (1.99) | 0.09  (0.57) | 0  (0) | 0.03  (0.27) | 0.23  (0.93) | 0.06  (0.45) | 0.15  (0.73) | 0.26  (0.98) | 0.08  (0.55) | 1.03  (2.01) | 0.15  (0.73) | 0.21  (0.91) |
| **Walk home from school (times/week)** | | | |  |  |  |  |  |  |  |  |  |  |  |  |  |  |
| Mean  (SD) | 2.15  (2.31) | 2.08  (2.18) | 1.20  (1.99) | 3.16  (2.21) | 3.17  (2.24) | 0.98  (1.71) | 2.30  (2.34) | 2.28  (2.27) | 2.65  (2.32) | 4.15  (1.71) | 1.03  (1.80) | 1.26  (1.93) | 3.08  (2.19) | 2.73  (2.41) | 1.44  (2.23) | 1.76  (2.17) | 2.47  (2.40) |
| **Cycle home from school (times/week)** | | | |  |  |  |  |  |  |  |  |  |  |  |  |  |  |
| Mean  (SD) | 0.41  (1.29) | 0.45  (1.26) | 2.45  (2.34) | 0.36  (1.13) | 0.20  (0.84) | 3.44  (2.00) | 0.08  (0.57) | 0  (0) | 0.05  (0.40) | 0.32  (1.06) | 0.07  (0.46) | 0.17  (0.82) | 0.15  (0.68) | 0.08  (0.55) | 1.04  (2.01) | 0.12  (0.68) | 0.19  (0.89) |
| **Any walking trips to or from school** | | |  |  |  |  |  |  |  |  |  |  |  |  |  |  |  |
| % 1 or more | 54.70 | 63.11 | 31.79 | 82.94 | 80.55 | 32.40 | 57.07 | 60.78 | 64.86 | 89.03 | 32.49 | 38.56 | 74.18 | 60.59 | 31.96 | 48.77 | 58.96 |
| **Any cycling trips to or from school** | | |  |  |  |  |  |  |  |  |  |  |  |  |  |  |  |
| % 1 or more | 11.38 | 14.93 | 56.19 | 12.94 | 6.89 | 83.10 | 3.24 | 0 | 3.02 | 10.32 | 3.34 | 5.81 | 7.66 | 2.65 | 21.52 | 5.28 | 5.97 |
| **Any cycling or walking trips to or from school** | | | |  |  |  |  |  |  |  |  |  |  |  |  |  |  |
| % 1 or more | 60.91 | 63.61 | 77.80 | 80.03 | 76.67 | 93.95 | 57.99 | 60.78 | 65.08 | 91.83 | 34.40 | 42.27 | 74.57 | 61.77 | 50.63 | 50.41 | 62.31 |
| **Regular walking to/from school** | | | |  |  |  |  |  |  |  |  |  |  |  |  |  |  |
| % Yes | 41.61 | 34.26 | 22.44 | 69.29 | 70.66 | 16.12 | 46.10 | 42.24 | 50.98 | 83.87 | 16.60 | 21.48 | 62.28 | 54.48 | 29.43 | 29.77 | 51.87 |
| **Regular cycling to/from school** | | | | |  |  |  |  |  |  |  |  |  |  |  |  |  |
| % Yes | 8.03 | 4.78 | 50.70 | 6.32 | 3.17 | 72.71 | 1.62 | 0 | 0.38 | 5.16 | 1.23 | 3.19 | 3.59 | 1.43 | 20.57 | 2.43 | 3.73 |

*Notes.* KL=Kuala Lumpur; Melb=Melbourne; Rep=Republic; HK=Hradec Kralove; SD=standard deviation.

# **Table S4.** Overall and site-specific perceived neighbourhood environment attributes (imputed data)

|  |  | **High-income countries** | | | | | | | | | | | **Low-middle-income countries** | | | | |
| --- | --- | --- | --- | --- | --- | --- | --- | --- | --- | --- | --- | --- | --- | --- | --- | --- | --- |
|  | **All sites** | **Australia Melb** | **Belgium Ghent** | **Czech Rep HK** | **Czech Rep Olomouc** | **Denmark Odense** | **China Hong Kong** | **Israel Haifa** | **Portugal Various cities** | **Spain Valencia** | **USA Baltimore** | **USA Seattle** | **Bangladesh Dhaka** | **Brazil Curitiba** | **India Chennai** | **Malaysia KL** | **Nigeria Gombe** |
| **N** | 6302 | 438 | 291 | 155 | 183 | 210 | 1295 | 232 | 184 | 465 | 485 | 443 | 92 | 493 | 316 | 752 | 268 |
| **Walking distance to school** | | | |  |  |  |  |  |  |  |  |  |  |  |  |  |  |
| Mean  (SD)  % 1-5 min  % 6-10 min  % 11-20 min  % 21-30 min  % 31+ min | 3.56  (1.35)  8.90  15.12  24.06  14.75  37.18 | 3.88  (1.28)  7.17  8.76  19.18  18.48  46.42 | 4.10  (1.25)  6.08  7.35  14.85  14.16  57.56 | 3.18  (1.31)  12.10  19.55  28.90  17.03  22.42 | 2.97  (1.40)  16.31  27.21  21.78  12.13  22.57 | 2.89  (1.25)  14.07  26.24  32.05  12.12  15.52 | 3.58  (1.35)  7.95  16.68  23.10  14.23  38.03 | 3.49  (1.21)  7.33  11.21  34.55  19.46  27.46 | 3.63  (1.30)  6.30  15.05  26.66  12.88  39.10 | 2.50  (1.14)  21.29  30.75  32.04  8.60  7.31 | 4.13  (1.17)  4.23  6.53  17.77  15.17  56.30 | 4.12  (1.15)  3.83  7.14  16.69  17.89  54.45 | 2.95  (1.14)  11.14  22.07  38.37  17.50  10.92 | 3.35  (1.37)  11.17  18.30  25.62  14.44  30.48 | 3.91  (1.32)  4.75  14.56  19.30  8.23  53.16 | 3.52  (1.37)  10.28  14.30  25.14  14.14  36.14 | 3.78  (1.07)  2.87  6.74  32.71  25.15  32.54 |
| **Residential density** | |  |  |  |  |  |  |  |  |  |  |  |  |  |  |  |  |
| Mean  (SD) | 208.3  (218) | 48.82  (92.29) | 71.49  (108.80) | 155.86  (110.2) | 115.73  (108.0) | 106.12  (109.96) | 468.40  (203.2) | 216.41  (148.4) | 120.57  (100.18) | 251.10  (134.67) | 39.01  (60.04) | 24.21  (35.07) | 177.30  (82.87) | 96.30  (126.68) | 65.64  (77.79) | 294.0  (225.77) | 271.38  (159.39) |
| **LUM diversity (excluding transit stops)** | | | |  |  |  |  |  |  |  |  |  |  |  |  |  |  |
| Mean  (SD) | 3.22 (0.89) | 3.06  (0.85) | 3.38  (0.83) | 3.30  (0.86) | 3.20  (0.87) | 2.99  (0.94) | 3.43  (0.81) | 3.01  (0.86) | 3.45  (0.80) | 4.19  (0.53) | 2.72  (0.90) | 2.73  (0.88) | 3.39  (0.65) | 2.97  (0.69) | 3.38  (0.69) | 2.99  (0.86) | 3.36  (0.79) |
| **Recreation facilities (excluding parks)** | | | |  |  |  |  |  |  |  |  |  |  |  |  |  |  |
| Mean  (SD) | 2.68 (0.90) | 2.81  (0.77) | 2.68  (0.90) | 2.96  (0.82) | 2.84  (0.83) | 3.69  (0.72) | 2.82  (0.86) | 2.43  (0.81) | 2.58  (0.90) | 2.92  (0.78) | 2.87  (0.91) | 2.91  (0.84) | 2.00  (0.73) | 2.38  (0.75) | 1.76  (0.57) | 2.35  (0.91) | 2.78  (0.53) |
| **Accessibility and walking facilities** | | |  |  |  |  |  |  |  |  |  |  |  |  |  |  |  |
| Mean  (SD) | 2.99  (0.58) | 3.13  (0.53) | 2.98  (0.58) | 3.16  (0.51) | 3.04  (0.55) | 3.08  (0.46) | 2.99  (0.50) | 3.10  (0.51) | 3.00  (0.44) | 3.58  (0.42) | 2.97  (0.57) | 2.82  (0.65) | 2.83  (0.57) | 2.88  (0.64) | 2.59  (0.59) | 2.89  (0.53) | 2.75  (0.65) |
| **Aesthetics** |  |  |  |  |  |  |  |  |  |  |  |  |  |  |  |  |  |
| Mean  (SD) | 2.52 (0.82) | 2.91  (0.79) | 2.27  (0.69) | 2.29  (0.64) | 2.24  (0.70) | 2.66  (0.76) | 2.47  (0.68) | 2.55  (0.82) | 2.39  (0.59) | 2.25  (0.74) | 3.04  (0.71) | 3.12  (0.66) | 1.81  (0.79) | 2.38  (0.87) | 1.52  (0.82) | 2.51  (0.68) | 2.91  (0.85) |
| **Traffic safety** |  |  |  |  |  |  |  |  |  |  |  |  |  |  |  |  |  |
| Mean  (SD) | 2.61 (0.66) | 2.74  (0.63) | 2.51  (0.59) | 2.78  (0.59) | 2.68  (0.61) | 2.94  (0.71) | 2.82  (0.50) | 2.36  (0.70) | 2.74  (0.50) | 2.61  (0.72) | 2.51  (0.59) | 2.66  (0.57) | 2.37  (0.60) | 2.17  (0.77) | 2.27  (0.68) | 2.47  (0.58) | 2.94  (0.88) |
| **Pedestrian infrastructure & safety** | | |  |  |  |  |  |  |  |  |  |  |  |  |  |  |  |
| Mean  (SD) | 2.85 (0.65) | 2.82  (0.54) | 2.68  (0.58) | 2.97  (0.59) | 2.90  (0.52) | 2.90  (0.70) | 2.95  (0.56) | 2.90  (0.74) | 2.87  (0.51) | 3.03  (0.62) | 2.80  (0.66) | 2.87  (0.64) | 2.46  (0.59) | 2.55  (0.79) | 2.92  (0.80) | 2.78  (0.56) | 2.95  (0.82) |
| **Crime safety** |  |  |  |  |  |  |  |  |  |  |  |  |  |  |  |  |  |
| Mean  (SD) | 2.81 (0.93) | 2.99  (0.84) | 3.09  (0.76) | 2.86  (0.79) | 2.79  (0.82) | 3.67  (0.59) | 2.69  (0.87) | 3.25  (0.86) | 2.93  (0.66) | 3.25  (0.79) | 2.95  (0.72) | 3.07  (0.73) | 1.97  (0.86) | 2.04  (0.84) | 3.03  (1.10) | 2.42  (0.89) | 2.72  (1.14) |
| **Transit stop proximity** | | |  |  |  |  |  |  |  |  |  |  |  |  |  |  |  |
| Mean  (SD) | 4.09  (1.17) | 4.53  (0.80) | 4.64  (0.76) | 4.33  (1.07) | 4.35  (0.94) | 4.61  (0.72) | 3.91  (1.08) | 4.51  (0.88) | 4.57  (0.76) | 4.77  (0.57) | 3.63  (1.44) | 4.03  (1.22) | 2.15  (1.25) | 4.74  (0.62) | 3.79  (1.11) | 3.30  (1.35) | 4.03  (1.03) |
| **Park proximity** | |  |  |  |  |  |  |  |  |  |  |  |  |  |  |  |  |
| Mean  (SD) | 3.01 (1.25) | 3.46  (1.19) | 3.11  (1.28) | 3.04  (1.16) | 2.73  (1.29) | 3.34  (1.27) | 3.35  (1.07) | 3.17  (1.18) | 2.93  (1.06) | 4.02  (0.83) | 2.90  (1.21) | 3.14  (1.15) | 1.86  (0.96) | 2.68  (0.97) | 2.18  (1.07) | 2.82  (1.23) | 1.05  (0.36) |
| **Trees** |  |  |  |  |  |  |  |  |  |  |  |  |  |  |  |  |  |
| Mean  (SD) | 2.97 (1.00) | 3.36  (0.85) | 2.55  (0.94) | 2.86  (0.90) | 2.80  (0.93) | 2.49  (1.07) | 3.19  (0.76) | 3.19  (0.87) | 2.73  (0.84) | 3.07  (0.97) | 3.34  (0.79) | 3.11  (0.90) | 1.99  (1.21) | 3.25  (0.95) | 2.06  (1.27) | 3.01  (0.88) | 1.88  (1.19) |
| **Buffers between street & footpaths** | | | |  |  |  |  |  |  |  |  |  |  |  |  |  |  |
| Mean  (SD) | 2.33 (1.11) | 2.87  (1.09) | 1.63  (0.78) | 2.60  (0.99) | 2.57  (0.98) | 2.10  (1.12) | 2.81  (0.87) | 1.60  (0.97) | 2.24  (0.83) | 1.65  (0.91) | 2.74  (1.08) | 2.32  (1.12) | 1.48  (0.85) | 2.53  (1.15) | 1.60  (1.11) | 2.51  (1.01) | 1.21  (0.66) |
| **Parking difficult** | |  |  |  |  |  |  |  |  |  |  |  |  |  |  |  |  |
| Mean  (SD) | 2.35 (1.09) | 2.18  (1.02) | 2.22  (1.01) | 2.10  (0.88) | 1.86  (0.90) | 1.40  (0.80) | 2.36  (0.89) | 2.89  (1.02) | 2.34  (0.80) | 3.17  (1.00) | 1.76  (0.87) | 1.68  (0.86) | 3.38  (0.93) | 2.63  (1.25) | 2.12  (1.35) | 2.84  (0.88) | 2.15  (1.24) |

*Notes.* KL=Kuala Lumpur; Melb=Melbourne; Rep=Republic; HK=Hradec Kralove; SES=Socio-economic-status; LUM=Land use mix; SD=standard deviation; min=minutes.

# **LATENT PROFILE ANALYSES OF ADOLESCENTS’ ACTIVE TRANSPORT TO/FROM SCHOOL**

Latent profile analyses (LPA) (Muthén, 2004) were conducted to identify groups of adolescents with different profiles of active travel to/from school (thereafter, latent profiles of active transport to/from school) based on their responses to items measuring weekly frequency of walking to school, walking from school, cycling to school and cycling from school. LPA is a probabilistic model-based clustering (of participants) approach which assumes that data arise from a mixture distribution with k components (i.e., k clusters or profiles), where k is not known a priori. LPA starts with comparing the fit of a 2-profile model vs. a single-profile model. The number of profiles is increased until the addition of more profiles is no longer warranted. In addition to the number of profiles, latent profile models can be specified in terms of whether and how the variable variances and covariances are estimated. This allows for four models to be specified: 1) equal variances and covariances fixed to zero; 2) varying variances and covariances fixed to zero; 3) equal variances and equal covariances; and 4) varying variances and varying covariances (Pastor et al., 2007). All the analyses were performed using R version 4.0.3 (R-core team 2020). The package `tidyLPA’ used to perform latent profile analysis (Rosenberg at al., 2019).

In this study, the optimal number of profiles was evaluated using several selection criteria. These included the Bayesian Information Criterion (BIC) (Nylund, Asparouhov, & Muthén, 2007), the Akaike Information Criterion (AIC) (Akaike, 1974) and the sample-size adjusted BIC (SABIC). Models producing smaller values for these criteria are preferred as they provide a better fit to the data. We also examined the entropy measure of classification uncertainty. Entropy values are bounded between 0 and 1. A value approaching 1 is an indication of a high degree of separation between the identified profiles and values greater than 0.70 indicate acceptable classification accuracy (Jung & Wickrama, 2008).

We explored different models with varying numbers of profiles ranging from two to five, and combination of indices and criteria were used to determine the model with the optimal number of profiles. For all models with two to five profiles, entropy values were very close to 1 and well above 0.7, suggesting a high degree of separation between profiles. A four-profile model of adolescents’ active transport to/from school with equal variances and covariance provided the best fit to the data according to both BIC and AIC values (Table S5). As a result, we deemed this model to be optimal. Table 4 (in the main manuscript) describes the four profiles. The first profile was represented by adolescents regularly walking to and from school; the second profile encompassed adolescents who did not walk to school but regularly walked from school; the third profile included those who regularly cycled to/from school; the last profile denoted adolescent who did not engage or seldom engaged in active transport to/from school.

**References**

1. Akaike H. A new look at the statistical model identification. IEEE Trans Autom Contr. 1974;19:716–23.
2. Jung T, Wickrama KAS. An introduction to latent class growth analysis and growth mixture modeling. Soc Personal Psychol Compass. 2008;2:302–17.
3. Muthén B. Latent variable analysis: growth mixture modeling and related techniques for longitudinal data. In: Kaplan D, editor. The SAGE handbook of quantitative methodology for the social sciences. Thousand Oaks: Sage Publications; 2004. p. 345–68.
4. Nylund KL, Asparouhov T, Muthén BO. Deciding on the number of classes in latent class analysis and growth mixture modeling: a Monte Carlo simulation study. Struct Equ Model. 2007;14(4):535–69.
5. Pastor DA, Barron KE, Miller BJ, Davis SL. A latent profile analysis of college students’ achievement goal orientation. Contemp Educ Psychol. 2007;32(1):8–47.
6. R Core Team. R: A language and environment for statistical computing. R Foundation for Statistical Computing, Vienna, Austria. 2020. URL https://www.R-project.org/.
7. Rosenberg JM, van Lissa CJ, Beymer PN, Anderson DJ, Schell M, Schmidt JA. tidyLPA: Easily carry out Latent Profile Analysis (LPA) using open-source or commercial software [R package]. 2019. https://data-edu.github.io/tidyLPA/

# **Table S5.** Fit indices for various latent profile analysis models

| **Model** | **No. profiles** | **Variance / covariance structure** | **AIC** | **BIC** | **CAIC** | **SABIC** | **Entropy** |
| --- | --- | --- | --- | --- | --- | --- | --- |
| 1 | 2 | Equal variances; zero covariances | 79428 | 79515 | 79528 | 79474 | 0.996 |
| 2 | 3 | As above | 54997 | 55118 | 55136 | 55061 | **0.999** |
| 3 | 4 | As above | 51636 | 51790 | 51813 | 51717 | 0.991 |
| 4 | 5 | As above | 48064 | 48252 | 48280 | 48163 | 0.997 |
| 5 | 2 | Equal variances and covariances | 68791 | 68918 | 68937 | 68858 | 0.995 |
| 6 | 3 | As above | 60052 | 60213 | 60237 | 60137 | 0.989 |
| 7 | 4 | As above | 47835 | **48029** | **48058** | **47937** | 0.995 |
| 8 | 5 | As above | **47817** | 48045 | 48079 | **47937** | 0.993 |

*Notes.* AIC: Akaike information criterion based on -2 log-likelihood and penalized by number of parameters; BIC: Bayesian information criterion; based on -2 log-likelihood and penalized by number of parameters adjusted by sample size; CAIC: Consistent Akaike information criterion; based on -2 log-likelihood and penalized by number of parameters adjusted by sample size; Entropy: a measure of classification uncertainty, reverse-coded so that 1 reflects complete certainty of classification, and 0 complete uncertainty. AIC is not adjusted for sample size, while CAIC and BIC are. Therefore, the latter are better measures of model fit than AIC. Models with unequal variances did not converge. Hence, their fit indices are not reported

# **TOTAL-EFFECT MODELS**

# **Table S6.** Total effects of parent-perceived neighbourhood environment characteristics on adolescents’ active transport to/from school (multiple imputations)

| **Neighbourhood characteristics [range of values]** | **Effect** | **Any active transport to/from school** | | | **Regular cycling to/from school^#^** | | | **Regular walking to/from school** | | |
| --- | --- | --- | --- | --- | --- | --- | --- | --- | --- | --- |
|  |  | OR | 95% CI | *p* | OR | 95% CI | *p* | OR | 95% CI | *p* |
| Residential density [0-1000] | Total | **1.001** | **1.000, 1.001** | **.006** | **0.999** | **0.998, 1.00** | **.050** | **1.001** | **1.000, 1.001** | **.002** |
| Land use mix – diversity^1^ [1-5] | Total | **1.38** | **1.26, 1.51** | **<.001** | 1.09 | 0.93, 1.29 | .284 | **1.46** | **1.33, 1.59** | **<.001** |
| Transit stop proximity [1-5] | Total | 1.00 | 0.89, 1.13 | .982 | 1.01 | 0.85, 1.19 | .935 | 0.97 | 0.88, 1.07 | .550 |
| Park proximity [1-5] | Total | 0.98 | 0.91, 1.06 | .674 | 0.93 | 0.82, 1.06 | .296 | 0.98 | 0.91, 1.05 | .601 |
| Buffers between streets & footpath [1-4] | Total | 0.98 | 0.91, 1.05 | .499 | 0.97 | 0.84, 1.11 | .647 | 0.99 | 0.92, 1.06 | .720 |
| Trees [1-4] | Total | 0.99 | 0.91, 1.07 | .707 | 0.94 | 0.81, 1.09 | .407 | 1.03 | 0.95, 1.11 | .458 |

*Notes.* ^1^ excluding transit stops; OR=odd ratio; CI = confidence intervals; *p* = *p*-value; in bold: effects significant at *p*<0.05; ^#^ N=5703 instead of 6302 because data from Israel, Portugal and Olomouc in the Czech Republic were excluded from the analyses due to 0% prevalence of regular cycling to/from school. The reference category of all outcome variables is ‘No’. Regular cycling/walking to/from school means cycling/walking to/from school 5-10 times a week. Analyses undertaken on 20 imputed datasets. Total effects for recreational facilities, traffic safety, pedestrian infrastructure, safety from crime, aesthetics, parking difficult and distance to school not shown as total and direct effect models are equivalent as no mediating variables of characteristic-outcome associations were included in the models. Complete case analyses are in the Supplementary Material (Tables S21, S22, S23). Model covariates are reported in Table S1.

# **Table S7.** Total effects of parent-perceived neighbourhood environment characteristics on latent profiles of adolescents’ active transport to/from school (multiple imputations)

| **Neighbourhood characteristics [range of values]** | **Effect** | **Walking to & from school vs. no active transport [n=5215]** | | | **Walking from school vs. no active transport [n=3727]** | | | **Cycling to & from school vs. no active transport^#^ [n=3389]** | | | **Walking to & from school vs. walking from school [n=2708]** | | |
| --- | --- | --- | --- | --- | --- | --- | --- | --- | --- | --- | --- | --- | --- |
|  |  | OR | 95% CI | *p* | OR | 95% CI | *p* | OR | 95% CI | *p* | OR | 95% CI | *p* |
| Residential density [0-1000] | Total | **1.001** | **1.000, 1.002** | **<.001** | 1.000 | 0.999, 1.001 | .338 | 0.999 | 0.998, 1.001 | .239 | 1.001 | 0.999, 1.002 | .059 |
| Land use mix – diversity^1^ [1-5] | Total | **1.57** | **1.42, 1.75** | **<.001** | **1.18** | **1.03, 1.35** | **.017** | 1.19 | 0.99, 1.43 | .068 | **1.36** | **1.15, 1.60** | **<.001** |
| Transit stop proximity [1-5] | Total | 0.93 | 0.83, 1.04 | .204 | 1.04 | 0.88, 1.21 | .666 | 1.03 | 0.85, 1.24 | .789 | 0.93 | 0.80, 1.10 | .403 |
| Park proximity [1-5] | Total | 0.98 | 0.90, 1.07 | .621 | 1.00 | 0.90, 1.11 | .973 | 0.92 | 0.79, 1.07 | .293 | 1.00 | 0.89, 1.13 | .958 |
| Buffers between streets & footpath [1-4] | Total | 1.00 | 0.93, 1.09 | .912 | 1.01 | 0.90, 1.14 | .858 | 0.96 | 0.82, 1.13 | .660 | 1.04 | 0.91, 1.18 | .605 |
| Trees [1-4] | Total | 1.04 | 0.95, 1.14 | .364 | 1.03 | 0.90, 1.17 | .696 | 0.95 | 0.80, 1.13 | .555 | 1.02 | 0.89, 1.18 | .746 |

*Notes.* ^1^ excluding transit stops; ^2^ excluding parks; OR=odd ratio; CI = confidence intervals; *p* = *p*-value; in bold: effects significant at *p*<0.05. ^#^ N=3389 instead of 3594 because data from Israel and Portugal were excluded from the analyses due to 0% prevalence of cycling to/from school. Analyses undertaken on 20 imputed datasets. Total effects for recreational facilities, traffic safety, pedestrian infrastructure, safety from crime, aesthetics, parking difficult and distance to school not shown as total and direct effect models are equivalent as no mediating variables of characteristic-outcome associations were included in the models. Complete case analyses are in the Supplementary Material (Tables S24, S25, S26 and S27). Model covariates are reported in Table S1.

# **MODERATION ANALYSES: Distance to school**

# **Table S8.** Distance to school as a moderator of total and direct effects of perceived neighbourhood environment characteristics on adolescents’ active transport to/from school (multiple imputations; N=6302)

| **Neighbourhood characteristics [range of values]** | **Effect** | **Any active transport to/from school** | | | **Regular cycling to/from school^#^** | | | **Regular walking to/from school** | | |
| --- | --- | --- | --- | --- | --- | --- | --- | --- | --- | --- |
|  |  | OR | 95% CI | *p* | OR | 95% CI | *p* | OR | 95% CI | *p* |
| Residential density [0-1000] | Total | 1.00 | 0.99, 1.00 | .116 | 1.00 | 0.999, 1.001 | .962 | **0.9996** | **0.9993, 0.9999** | **.008** |
|  | Direct | 1.00 | 0.99, 1.00 | .084 | 1.00 | 0.999, 1.001 | .987 | **0.9996** | **0.9993, 0.9999** | **.005** |
| Land use mix – diversity^1^ [1-5] | Total | **0.90** | **0.83, 0.97** | **.008** | **1.18** | **1.05, 1.32** | **.005** | **0.90** | **0.84, 0.97** | **.004** |
|  | Direct | **0.90** | **0.83, 0.97** | **.009** | **1.19** | **1.06, 1.34** | **.004** | **0.90** | **0.83, 0.96** | **.003** |
| Transit stop proximity [1-5] | Total | 0.96 | 0.91, 1.02 | .240 | 1.08 | 0.98, 1.18 | .103 | 0.99 | 0.93, 1.04 | .644 |
|  | Direct | 0.96 | 0.91, 1.02 | .222 | 1.08 | 0.99, 1.18 | .098 | 0.99 | 0.93, 1.04 | .644 |
| Recreational facilities^2^ [1-5] | Total^a^ | 0.99 | 0.92, 1.07 | .814 | 1.05 | 0.95, 1.16 | .309 | 0.95 | 0.89, 1.02 | .177 |
| Park proximity [1-5] | Total | **0.94** | **0.89, 0.99** | **.032** | 1.00 | 0.93, 1.07 | .943 | 0.95 | 0.90, 1.00 | .053 |
|  | Direct | **0.94** | **0.89, 1.00** | **.035** | 1.00 | 0.93, 1.08 | .903 | **0.95** | **0.90, 1.00** | **.050** |
| Accessibility & walking facilities [1-4] | Total^a^ | **0.86** | **0.75, 0.97** | **.019** | 1.14 | 0.97, 1.34 | .101 | **0.89** | **0.80, 1.00** | **.043** |
| Traffic safety [1-4] | Total^a^ | 1.02 | 0.93, 1.13 | .638 | 1.13 | 0.98, 1.29 | .090 | 0.96 | 0.88, 1.05 | .385 |
| Pedestrian infrastructure [1-4] | Total^a^ | 0.91 | 0.81, 1.01 | .064 | 1.08 | 0.94, 1.24 | .254 | 0.94 | 0.85, 1.05 | .275 |
| Safety from crime [1-4] | Total^a^ | 0.99 | 0.92, 1.06 | .693 | 1.02 | 0.91, 1.13 | .772 | 1.00 | 0.93, 1.06 | .899 |
| Aesthetics [1-4] | Total^a^ | 1.04 | 0.96, 1.13 | .297 | 1.02 | 0.91, 1.14 | .749 | 0.99 | 0.92, 1.07 | .809 |
| Buffers between streets & footpath [1-4] | Total | 1.03 | 0.97, 1.10 | .309 | 0.94 | 0.87, 1.03 | .180 | 1.00 | 0.94, 1.06 | .989 |
|  | Direct | 1.03 | 0.97, 1.10 | .357 | 0.94 | 0.87, 1.03 | .192 | 1.00 | 0.94, 1.06 | .902 |
| Parking difficult [1-4] | Total^a^ | 0.99 | 0.92, 1.06 | .755 | 1.01 | 0.93, 1.10 | .828 | 0.99 | 0.93, 1.05 | .791 |
| Trees [1-4] | Total | 0.97 | 0.91, 1.04 | .422 | 1.06 | 0.97, 1.15 | .198 | **0.92** | **0.87, 0.98** | **.013** |
|  | Direct | 0.97 | 0.91, 1.04 | .448 | 1.06 | 0.97, 1.15 | .201 | **0.93** | **0.87, 0.99** | **.017** |

*Notes.* ^1^ excluding transit stops; ^2^ excluding parks; OR=odd ratio; CI = confidence intervals; *p* = *p*-value; in bold: effects significant at *p*<0.05; ^#^ N=5703 instead of 6302 because data from Israel, Portugal and Olomouc in the Czech Republic were excluded from the analyses due to 0% prevalence of regular cycling to/from school. The reference category of all outcome variables is ‘No’. Regular cycling/walking to/from school means cycling/walking to/from school 5-10 times a week. Analyses undertaken on 20 imputed datasets. ^a^Total and direct effects are equivalent as no mediating variables of characteristic-outcome associations were included in the models. Complete case analyses are in Tables S28, S29 and S30. Model covariates are reported in Table S1. Regression coefficients (ORs) are those of the interaction terms between a specific environmental attribute and parent-perceived distance to school.

# **Table S9.** Distance to school as a moderator of total and direct effects of perceived neighbourhood environment characteristics on latent profiles of adolescents’ active transport to/from school (multiple imputations)

| **Neighbourhood characteristics [range of values]** | **Effect** | **Walking to & from school vs. no active transport [n=5215]** | | | **Walking from school vs. no active transport [n=3727]** | | | **Cycling to & from school vs. no active transport^#^ [n=3389]** | | | **Walking to & from school vs. walking from school [n=2708]** | | |
| --- | --- | --- | --- | --- | --- | --- | --- | --- | --- | --- | --- | --- | --- |
|  |  | OR | 95% CI | *p* | OR | 95% CI | *p* | OR | 95% CI | *P* | OR | 95% CI | *p* |
| Residential density [0-1000] | Total | 0.999 | 0.999, 1.000 | .188 | 0.999 | 0.999, 1.000 | .553 | 0.999 | 0.999, 1.000 | .133 | 0.999 | 0.999, 1.000 | .241 |
|  | Direct | 0.999 | 0.999, 1.000 | .133 | 0.999 | 0.999, 1.000 | .491 | 0.999 | 0.999, 1.000 | .154 | 0.999 | 0.999, 1.000 | .215 |
| Land use mix – diversity^1^ [1-5] | Total | 0.91 | 0.83, 1.00 | .052 | 0.95 | 0.85, 1.06 | .345 | 0.93 | 0.81, 1.08 | .336 | 0.99 | 0.88, 1.13 | .928 |
|  | Direct | **0.91** | **0.83, 1.00** | **.050** | 0.94 | 0.85, 1.05 | .306 | 0.94 | 0.81, 1.09 | .390 | 1.00 | 0.88, 1.13 | .964 |
| Transit stop proximity [1-5] | Total | 1.01 | 0.94, 1.08 | .819 | 0.97 | 0.90, 1.05 | .504 | 1.02 | 0.91, 1.14 | .796 | 1.04 | 0.95, 1.15 | .393 |
|  | Direct | 1.01 | 0.94, 1.08 | .745 | 0.97 | 0.90, 1.05 | .470 | 1.01 | 0.90, 1.14 | .809 | 1.04 | 0.94, 1.14 | .434 |
| Recreational facilities^2^ [1-5] | Total^a^ | 1.01 | 0.93, 1.09 | .840 | 0.94 | 0.85, 1.05 | .272 | 0.96 | 0.84, 1.10 | .546 | 1.05 | 0.94, 1.18 | .348 |
| Park proximity [1-5] | Total | 0.99 | 0.93, 1.05 | .670 | 0.94 | 0.87, 1.02 | .139 | 0.95 | 0.86, 1.06 | .389 | 1.07 | 0.98, 1.17 | .128 |
|  | Direct | 0.99 | 0.93, 1.05 | .642 | 0.94 | 0.87, 1.02 | .126 | 0.96 | 0.87, 1.07 | .475 | 1.07 | 0.98, 1.17 | .125 |
| Accessibility & walking facilities [1-4] | Total^a^ | 0.91 | 0.79, 1.04 | .178 | 0.89 | 0.75, 1.06 | .203 | 1.01 | 0.81, 1.26 | .931 | 1.06 | 0.88, 1.28 | .559 |
| Traffic safety [1-4] | Total^a^ | 1.04 | 0.92, 1.17 | .556 | 0.94 | 0.82, 1.09 | .410 | 1.17 | 0.97, 1.42 | .108 | 1.14 | 0.98, 1.32 | .097 |
| Pedestrian infrastructure [1-4] | Total^a^ | 0.95 | 0.83, 1.08 | .407 | 0.93 | 0.80, 1.08 | .355 | 1.04 | 0.86, 1.26 | .653 | 1.01 | 0.85, 1.20 | .872 |
| Safety from crime [1-4] | Total^a^ | 1.01 | 0.93, 1.10 | .844 | 0.92 | 0.83, 1.01 | .085 | 0.93 | 0.80, 1.08 | .344 | **1.13** | **1.01, 1.27** | **.028** |
| Aesthetics [1-4] | Total^a^ | 1.04 | 0.94, 1.14 | .447 | 0.95 | 0.85, 1.06 | .364 | 1.08 | 0.93, 1.26 | .319 | **1.13** | **1.01, 1.28** | **.040** |
| Buffers between streets & footpath [1-4] | Total | 1.05 | 0.97, 1.13 | .201 | 1.01 | 0.93, 1.11 | .766 | 1.04 | 0.92, 1.17 | .537 | **1.12** | **1.02, 1.25** | **.025** |
|  | Direct | 1.04 | 0.97, 1.13 | .267 | 1.01 | 0.92, 1.10 | .825 | 1.05 | 0.93, 1.18 | .457 | **1.13** | **1.02, 1.25** | **.024** |
| Parking difficult [1-4] | Total^a^ | 0.99 | 0.92, 1.06 | .747 | 1.00 | 0.91, 1.10 | .975 | 0.95 | 0.84, 1.08 | .455 | 0.97 | 0.88, 1.07 | .519 |
| Trees [1-4] | Total | 0.95 | 0.88, 1.02 | .159 | 0.94 | 0.86, 1.04 | .251 | 1.08 | 0.96, 1.23 | .197 | 1.05 | 0.95, 1.16 | .318 |
|  | Direct | 0.95 | 0.88, 1.02 | .189 | 0.94 | 0.86, 1.04 | .248 | 1.09 | 0.96, 1.23 | .175 | 1.06 | 0.96, 1.17 | .271 |

*Notes.* ^1^ excluding transit stops; ^2^ excluding parks; OR=odd ratio; CI = confidence intervals; *p* = *p*-value; in bold: effects significant at *p*<0.05. ^#^ N=3389 instead of 3594 because data from Israel and Portugal were excluded from the analyses due to 0% prevalence of cycling to/from school. Analyses undertaken on 20 imputed datasets. ^a^Total and direct effects are equivalent as no mediating variables of characteristic-outcome associations were included in the models. Complete case analyses are in Tables S31, S32, S33 and S34. Model covariates are reported in Table S1. Regression coefficients (ORs) are those of the interaction term between a specific environmental attribute and parent-reported distance to school.

# **Table S10.** Total and direct effects of parent-perceived park proximity on adolescents’ any vs. no active transport to/from school and regular vs. occasional/no walking to/from school by distance to school (multiple imputations; N = 6302)

| **Distance to school (walking)** | **Effects** | **Any active transport to/from school** | | | **Regular walking to/from school** | | |
| --- | --- | --- | --- | --- | --- | --- | --- |
|  |  | OR | 95% CI | *p* | OR | 95% CI | *p* |
| 1-5 minutes | Total | 1.16 | 0.96, 1.39 | .118 | 1.09 | 0.94, 1.25 | .246 |
|  | Direct | 1.14 | 0.95, 1.38 | .150 | 1.08 | 0.94, 1.25 | .269 |
| 6-10 minutes | Total | 1.09 | 0.95, 1.24 | .214 | 1.03 | 0.93, 1.14 | .524 |
|  | Direct | 1.08 | 0.94, 1.23 | .275 | 1.03 | 0.93, 1.14 | .585 |
| 11-20 minutes | Total | 1.02 | 0.93, 1.12 | .611 | 0.98 | 0.91, 1.06 | .630 |
|  | Direct | 1.02 | 0.92, 1.12 | .751 | 0.98 | 0.91, 1.05 | .542 |
| 21-30 minutes | Total | 0.96 | 0.90, 1.04 | .325 | 0.93 | 0.86, 1.01 | .088 |
|  | Direct | 0.96 | 0.89, 1.03 | .244 | 0.93 | 0.86, 1.01 | .067 |
| 31+ minutes | Total | **0.91** | **0.83, 0.99** | **.038** | **0.89** | **0.79, 0.99** | **.035** |
|  | Direct | **0.90** | **0.82, 0.99** | **.028** | **0.88** | **0.79, 0.98** | **.027** |

*Notes.* excluding parks; OR=odd ratio; CI = confidence intervals; *p* = *p*-value; in bold: effects significant at *p*<0.05; The reference category of all outcome variables is ‘No’. Regular walking to/from school means walking to/from school 5-10 times a week. Analyses undertaken on 20 imputed datasets. Complete case analyses are in Tables S28 and S30. Model covariates are reported in Table S1.

# **Table S11.** Total and direct effects of parent-perceived neighbourhood environment characteristics on adolescents’ walking to and from school vs. walking from school by distance to school (multiple imputations; N = 2708)

| **Distance to school (walking)** | **Safety from crime^a^** | | | **Aesthetics^a^** | | | **Buffers between streets & footpaths (total effect)** | | | **Buffers between streets & footpaths (direct effect)** | | |
| --- | --- | --- | --- | --- | --- | --- | --- | --- | --- | --- | --- | --- |
|  | OR | 95% CI | *p* | OR | 95% CI | *p* | OR | 95% CI | *p* | OR | 95% CI | *p* |
| 1-5 minutes | 0.78 | 0.56, 1.10 | .155 | 0.77 | 0.55, 1.08 | .134 | 0.81 | 0.63, 1.05 | .110 | 0.81 | 0.63, 1.04 | .106 |
| 6-10 minutes | 0.89 | 0.68, 1.15 | .363 | 0.88 | 0.68, 1.13 | .304 | 0.92 | 0.77, 1.09 | .322 | 0.91 | 0.76, 1.09 | .324 |
| 11-20 minutes | 1.01 | 0.81, 1.24 | .956 | 1.00 | 0.81, 1.22 | .963 | 1.03 | 0.90, 1.18 | .675 | 1.03 | 0.89, 1.19 | .692 |
| 21-30 minutes | 1.14 | 0.92, 1.42 | .236 | 1.13 | 0.91, 1.41 | .281 | 1.16 | 0.98, 1.37 | .084 | 1.16 | 0.97, 1.38 | .101 |
| 31+ minutes | 1.30 | 0.98, 1.71 | .066 | 1.28 | 0.96, 1.71 | .097 | **1.30** | **1.03, 1.65** | **.031** | **1.31** | **1.02, 1.68** | **.037** |

*Notes.* excluding parks; OR=odd ratio; CI = confidence intervals; *p* = *p*-value; in bold: effects significant at *p*<0.05. ^a^Total and direct effects are equivalent as no mediating variables of characteristic-outcome associations were included in the models. Regular walking to/from school means walking to/from school 5-10 times a week. Analyses undertaken on 20 imputed datasets. Complete case analyses are in Table S34. Model covariates are reported in Table S1.

# **MODERATION ANALYSES: Adolescent’s sex**

# **Table S12.** Adolescents’ sex as a moderator of total and direct effects of perceived neighbourhood environment characteristics on adolescents’ active transport to/from school (multiple imputations; N=6302)

| **Neighbourhood characteristics [range of values]** | **Effect** | **Any active transport to/from school** | | | **Regular cycling to/from school^#^** | | | **Regular walking to/from school** | | |
| --- | --- | --- | --- | --- | --- | --- | --- | --- | --- | --- |
|  |  | OR | 95% CI | *p* | OR | 95% CI | *p* | OR | 95% CI | *p* |
| Residential density [0-1000] | Total | 1.00 | 0.99, 1.00 | .658 | 1.000 | 0.999, 1.002 | .553 | 1.00 | 0.99, 1.00 | .814 |
|  | Direct | 1.00 | 0.99, 1.00 | .849 | 1.000 | 0.999, 1.002 | .617 | 1.00 | 0.99, 1.00 | .828 |
| Land use mix – diversity^1^ [1-5] | Total | 1.05 | 0.90, 1.22 | .556 | **1.37** | **1.00, 1.86** | **.047** | 1.11 | 0.94, 1.30 | .214 |
|  | Direct | 1.05 | 0.90, 1.22 | .534 | **1.40** | **1.02, 1.90** | **.035** | 1.11 | 0.95, 1.31 | .195 |
| Transit stop proximity [1-5] | Total | 1.02 | 0.91, 1.15 | .720 | 1.18 | 0.90, 1.54 | .233 | 1.02 | 0.90, 1.15 | .779 |
|  | Direct | 1.03 | 0.91, 1.17 | .616 | 1.20 | 0.91, 1.57 | .192 | 1.02 | 0.91, 1.16 | .699 |
| Recreational facilities^2^ [1-5] | Total^a^ | 1.10 | 0.94, 1.29 | .230 | **1.63** | **1.23, 2.16** | **<.001** | 1.01 | 0.87, 1.18 | .881 |
| Park proximity [1-5] | Total | 1.06 | 0.95, 1.17 | .305 | **1.25** | **1.02, 1.52** | **.029** | 1.04 | 0.93, 1.15 | .514 |
|  | Direct | 1.05 | 0.95, 1.17 | .326 | **1.24** | **1.02, 1.52** | **.033** | 1.04 | 0.93, 1.15 | .524 |
| Accessibility & walking facilities [1-4] | Total^a^ | 1.10 | 0.87, 1.39 | .405 | **1.87** | **1.21, 2.89** | **.005** | 0.98 | 0.77, 1.23 | .850 |
| Traffic safety [1-4] | Total^a^ | 1.05 | 0.86, 1.29 | .623 | 1.04 | 0.71, 1.52 | .829 | 0.98 | 0.81, 1.20 | .864 |
| Pedestrian infrastructure [1-4] | Total^a^ | 1.12 | 0.91, 1.36 | .278 | 1.11 | 0.75, 1.63 | .600 | 0.97 | 0.80, 1.19 | .806 |
| Safety from crime [1-4] | Total^a^ | 1.04 | 0.90, 1.21 | .591 | 1.12 | 0.83, 1.51 | .455 | 1.05 | 0.90, 1.22 | .531 |
| Aesthetics [1-4] | Total^a^ | 1.04 | 0.88, 1.22 | .651 | 1.07 | 0.80, 1.44 | .634 | 0.96 | 0.81, 1.14 | .662 |
| Buffers between streets & footpath [1-4] | Total | 1.03 | 0.91, 1.17 | .600 | 1.03 | 0.81, 1.30 | .833 | 0.93 | 0.82, 1.06 | .274 |
|  | Direct | 1.02 | 0.90, 1.16 | .726 | 1.01 | 0.80, 1.28 | .925 | 0.92 | 0.81 1.05 | .215 |
| Parking difficult [1-4] | Total^a^ | 0.92 | 0.80, 1.04 | .185 | 1.13 | 0.90, 1.42 | .289 | 0.98 | 0.86, 1.11 | .741 |
| Trees [1-4] | Total | 1.05 | 0.92, 1.21 | .460 | 1.25 | 0.98, 1.58 | .068 | 0.92 | 0.81, 1.05 | .223 |
|  | Direct | 1.05 | 0.92, 1.21 | .459 | 1.24 | 0.98, 1.57 | .078 | 0.92 | 0.81, 1.05 | .243 |
| Distance to school [1-5] | Total^a^ | 0.90 | 0.79, 1.02 | .093 | 0.93 | 0.78, 1.12 | .464 | 0.93 | 0.83, 1.05 | .231 |

*Notes.* ^1^ excluding transit stops; ^2^ excluding parks; OR=odd ratio; CI = confidence intervals; *p* = *p*-value; in bold: effects significant at *p*<0.05; ^#^ N=5703 instead of 6302 because data from Israel, Portugal and Olomouc in the Czech Republic were excluded from the analyses due to 0% prevalence of regular cycling to/from school. The reference category of all outcome variables is ‘No’. Regular cycling/walking to/from school means cycling/walking to/from school 5-10 times a week. Analyses undertaken on 20 imputed datasets. ^a^Total and direct effects are equivalent as no mediating variables of characteristic-outcome associations were included in the models. Complete case analyses are in Tables S35, S36 and S37. Model covariates are reported in Table S1. Regression coefficients (ORs) are those of the interaction terms between a specific environmental attribute and adolescent sex.

# **Table S13.** Adolescents’ sex as a moderator of total and direct effects of perceived neighbourhood environment characteristics on latent profiles of adolescents’ active transport to/from school (multiple imputations)

| **Neighbourhood characteristics [range of values]** | **Effect** | **Walking to & from school vs. no active transport [n=5215]** | | | **Walking from school vs. no active transport [n=3727]** | | | **Cycling to & from school vs. no active transport^#^ [n=3389]** | | | **Walking to & from school vs. walking from school [n=2708]** | | |
| --- | --- | --- | --- | --- | --- | --- | --- | --- | --- | --- | --- | --- | --- |
|  |  | OR | 95% CI | *p* | OR | 95% CI | *p* | OR | 95% CI | *p* | OR | 95% CI | *P* |
| Residential density [0-1000] | Total | 1.000 | 0.999, 1.001 | .473 | 0.999 | 0.998, 1.000 | .260 | 1.001 | 0.999, 1.003 | .202 | 1.001 | 0.999, 1.002 | .153 |
|  | Direct | 1.000 | 0.999, 1.001 | .764 | 0.999 | 0.998, 1.000 | .172 | 1.001 | 0.999, 1.003 | .234 | 1.001 | 0.999, 1.002 | .200 |
| Land use mix – diversity^1^ [1-5] | Total | 1.12 | 0.93, 1.34 | .244 | 0.99 | 0.78, 1.25 | .912 | 1.33 | 0.94, 1.88 | .103 | 1.07 | 0.81, 1.42 | .619 |
|  | Direct | 1.12 | 0.93, 1.36 | .233 | 0.98 | 0.78, 1.25 | .898 | 1.36 | 0.96, 1.93 | .087 | 1.08 | 0.82, 1.44 | .576 |
| Transit stop proximity [1-5] | Total | 0.98 | 0.85, 1.13 | .752 | 1.03 | 0.86, 1.24 | .746 | 1.12 | 0.83, 1.51 | .445 | 0.96 | 0.78, 1.20 | .741 |
|  | Direct | 0.99 | 0.85, 1.14 | .844 | 1.03 | 0.85, 1.24 | .768 | 1.14 | 0.84, 1.55 | .385 | 0.98 | 0.78, 1.21 | .828 |
| Recreational facilities^2^ [1-5] | Total^a^ | 1.04 | 0.88, 1.24 | .658 | 0.88 | 0.69, 1.11 | .282 | **1.53** | **1.10, 2.11** | **.010** | 1.15 | 0.87, 1.50 | .324 |
| Park proximity [1-5] | Total | 1.07 | 0.94, 1.21 | .290 | 0.89 | 0.75, 1.06 | .193 | **1.30** | **1.02, 1.65** | **.032** | 1.15 | 0.94, 1.41 | .168 |
|  | Direct | 1.07 | 0.94, 1.21 | .312 | 0.90 | 0.75, 1.07 | .216 | **1.28** | **1.01, 1.63** | **.043** | 1.15 | 0.94, 1.41 | .182 |
| Accessibility & walking facilities [1-4] | Total^a^ | 1.00 | 0.76, 1.31 | .986 | 1.01 | 0.70, 1.45 | .968 | 1.55 | 0.94, 2.56 | .089 | 1.06 | 0.70, 1.60 | .782 |
| Traffic safety [1-4] | Total^a^ | 0.98 | 0.77, 1.23 | .845 | 1.18 | 0.87, 1.61 | .290 | 0.91 | 0.58, 1.42 | .681 | 0.87 | 0.61, 1.23 | .421 |
| Pedestrian infrastructure [1-4] | Total^a^ | 0.96 | 0.76, 1.21 | .707 | 1.08 | 0.78, 1.50 | .645 | 0.99 | 0.64, 1.52 | .953 | 0.89 | 0.61, 1.29 | .530 |
| Safety from crime [1-4] | Total^a^ | 0.98 | 0.83, 1.17 | .851 | 1.05 | 0.84, 1.32 | .659 | 1.08 | 0.77, 1.52 | .645 | 0.94 | 0.73, 1.21 | .646 |
| Aesthetics [1-4] | Total^a^ | 0.97 | 0.79, 1.20 | .801 | 0.98 | 0.76, 1.27 | .877 | 0.97 | 0.68, 1.36 | .847 | 1.07 | 0.80, 1.44 | .643 |
| Buffers between streets & footpath [1-4] | Total | 0.99 | 0.86, 1.14 | .886 | 0.88 | 0.73, 1.07 | .192 | 0.96 | 0.73, 1.28 | .803 | 1.14 | 0.92, 1.42 | .241 |
|  | Direct | 0.97 | 0.84, 1.12 | .664 | 0.88 | 0.72, 1.07 | .197 | 0.95 | 0.72, 1.26 | .729 | 1.14 | 0.91, 1.42 | .265 |
| Parking difficult [1-4] | Total^a^ | 1.09 | 0.94, 1.26 | .277 | 0.83 | 0.68, 1.03 | .086 | 1.19 | 0.91, 1.55 | .207 | **1.35** | **1.06, 1.72** | **.017** |
| Trees [1-4] | Total | 0.96 | 0.82, 1.13 | .651 | 0.90 | 0.71, 1.12 | .342 | 1.20 | 0.90, 1.61 | .223 | 1.16 | 0.90, 1.50 | .239 |
|  | Direct | 0.95 | 0.81, 1.12 | .562 | 0.90 | 0.71, 1.13 | .355 | 1.21 | 0.90, 1.63 | .214 | 1.18 | 0.91, 1.53 | .203 |
| Distance to school [1-5] | Total^a^ | 0.89 | 0.78, 1.03 | .121 | 0.93 | 0.78, 1.11 | .414 | 1.03 | 0.79, 1.33 | .827 | 0.94 | 0.77, 1.14 | .523 |

*Notes.* ^1^ excluding transit stops; ^2^ excluding parks; OR=odd ratio; CI = confidence intervals; *p* = *p*-value; in bold: effects significant at *p*<0.05. ^#^ N=3389 instead of 3594 because data from Israel and Portugal were excluded from the analyses due to 0% prevalence of cycling to/from school. Analyses undertaken on 20 imputed datasets. ^a^Total and direct effects are equivalent as no mediating variables of characteristic-outcome associations were included in the models. Complete case analyses are in Tables S38, S39, S40 and S41. Model covariates are reported in Table S1. Regression coefficients (ORs) are those of the interaction term between a specific environmental attribute and adolescent sex.

# **Table S14.** Sex-specific effects of land use mix-diversity, neighbourhood accessibility and walking facilities, park proximity and recreation facilities on regular cycling to/from school vs. no or less frequent cycling to/from school (multiple imputations; N = 5703^#^)

| **Effect** |  | **Females** | | | **Males** | | |
| --- | --- | --- | --- | --- | --- | --- | --- |
|  |  | OR | 95% CI | *p* | OR | 95% CI | *p* |
| Land use mix-diversity | Total | **1.30** | **1.02, 1.90** | **.032** | 0.95 | 0.77, 1.17 | .642 |
|  | Direct | **1.36** | **1.04, 1.79** | **.025** | 0.98 | 0.77, 1.24 | .865 |
| Neighbourhood accessibility and walking facilities | Total^a^ | 1.42 | 0.97, 2.07 | .070 | 0.76 | 0.55, 1.05 | .101 |
| Park proximity | Total | 1.05 | 0.89, 1.23 | .598 | **0.84** | **0.71, 0.99** | **.037** |
|  | Direct | 1.03 | 0.87, 1.21 | .746 | **0.83** | **0.70, 0.98** | **.028** |
| Recreation facilities | Total^a^ | 1.25 | 0.97, 1.61 | .084 | **0.77** | **0.60, 0.98** | **.037** |

*Notes.* OR=odd ratio; CI = confidence intervals; *p* = *p*-value; in bold: effects significant at *p*<0.05. ^#^ N=5703 instead of 6302 because data from Israel, Portugal and Olomouc in the Czech Republic were excluded from the analyses due to 0% prevalence of regular cycling to/from school. Analyses undertaken on 20 imputed datasets. ^a^Total and direct effects are equivalent as no mediating variables of characteristic-outcome associations were included in the models. Complete case analyses are in Table S36. Model covariates are reported in Table S1.

# **Table S15.** Sex-specific effects of park proximity and recreational facilities on cycling to and from school vs. no active transport to/from school (multiple imputations; N = 3389^#^)

|  | **Effect** | **Females** | | | **Males** | | |
| --- | --- | --- | --- | --- | --- | --- | --- |
|  |  | OR | 95% CI | *p* | OR | 95% CI | *p* |
| Park proximity | Total | 1.06 | 0.87, 1.29 | .580 | **0.82** | **0.67, 0.99** | **.040** |
|  | Direct | 1.03 | 0.84, 1.27 | .744 | **0.81** | **0.66, 0.98** | **.033** |
| Recreation facilities | Total^a^ | 1.24 | 0.88, 1.75 | .321 | **0.70** | **0.50, 0.99** | **.047** |

*Notes.* OR=odd ratio; CI = confidence intervals; *p* = *p*-value; in bold: effects significant at *p*<0.05. ^#^ N=3389 instead of 3594 because data from Israel and Portugal were excluded from the analyses due to 0% prevalence of cycling to/from school. Analyses undertaken on 20 imputed datasets. Complete case analyses are in Table S40. Model covariates are reported in Table S1.

# **Table S16.** Sex-specific effects of parking being difficult on regular walking to/from school vs. walking from school (multiple imputations; N = 2708)

| **Effect** | **Females** | | | **Males** | | |
| --- | --- | --- | --- | --- | --- | --- |
|  | OR | 95% CI | *p* | OR | 95% CI | *P* |
| Total^a^ | **1.18** | **0.99, 1.41** | **.062** | 0.88 | 0.73, 1.05 | .152 |

*Notes.* OR=odd ratio; CI = confidence intervals; *p* = *p*-value; in bold: effects significant at *p*<0.05. ^a^Total and direct effects are equivalent as no mediating variables of characteristic-outcome associations were included in the models. Analyses undertaken on 20 imputed datasets. Complete case analyses are in Table S41. Model covariates are reported in Table S1.

# **MODERATION ANALYSES: City**

# **Table S17.** Difference in Akaike Information Criterion (AIC) values between models with and without city as a moderator of total and direct effects of perceived neighbourhood environment characteristics on adolescents’ active transport to/from school (multiple imputations; N=6302)

| **Neighbourhood characteristics [range of values]** | **Effect** | **Any active transport to/from school** | **Regular cycling to/from school^#^** | **Regular walking to/from school** |
| --- | --- | --- | --- | --- |
| Residential density [0-1000] | Total | 63.4 | 469.3 | 64.4 |
|  | Direct | 64.5 | 485.8 | 124.4 |
| Land use mix – diversity^1^ [1-5] | Total | 91.7 | 563.7 | 57.0 |
|  | Direct | 82.3 | 605.6 | **-17.2** |
| Transit stop proximity [1-5] | Total | 81.4 | 734.4 | 99.5 |
|  | Direct | 86.0 | 753.3 | 117.2 |
| Recreational facilities^2^ [1-5] | Total^a^ | 113.1 | 192.4 | 85.0 |
| Park proximity [1-5] | Total | 66.2 | 375.0 | 53.1 |
|  | Direct | 66.3 | 387.4 | 54.2 |
| Accessibility & walking facilities [1-4] | Total^a^ | 55.3 | 380.7 | 61.0 |
| Traffic safety [1-4] | Total^a^ | 56.6 | 133.1 | 51.8 |
| Pedestrian infrastructure [1-4] | Total^a^ | 47.7 | 429.8 | 54.5 |
| Safety from crime [1-4] | Total^a^ | 105.6 | 415.3 | 69.0 |
| Aesthetics [1-4] | Total^a^ | 90.5 | 332.8 | 72.3 |
| Buffers between streets & footpath [1-4] | Total | 63.4 | 282.6 | 52.2 |
|  | Direct | 64.6 | 290.4 | 52.8 |
| Parking difficult [1-4] | Total^a^ | 246.3 | 190.1 | -0.2 |
| Trees [1-4] | Total | 49.5 | 2373.6 | 49.5 |
|  | Direct | 50.2 | 2396.6 | 52.9 |
| Distance to school [1-5] | Total^a^ | 891.8 | 214.4 | 741.6 |

*Notes.* ^1^ excluding transit stops; ^2^ excluding parks; in bold: effects significant because AIC value of model with interaction term 10+ smaller than that without interaction term; ^#^ N=5703 instead of 6302 because data from Israel, Portugal and Olomouc in the Czech Republic were excluded from the analyses due to 0% prevalence of regular cycling to/from school. The reference category of all outcome variables is ‘No’. Regular cycling/walking to/from school means cycling/walking to/from school 5-10 times a week. Analyses undertaken on 20 imputed datasets. ^a^Total and direct effects are equivalent as no mediating variables of characteristic-outcome associations were included in the models. Complete case analyses are in Tables S42, S43 and S44. Model covariates are reported in Table S1.

# **Table S18.** Difference in Akaike Information Criterion (AIC) values between models with and without city as a moderator of total and direct effects of perceived neighbourhood environment characteristics on latent profiles of adolescents’ active transport to/from school (multiple imputations)

| **Neighbourhood characteristics [range of values]** | **Effect** | **Walking to & from school vs. no active transport [n=5215]** | **Walking from school vs. no active transport [n=3727]** | **Cycling to & from school vs. no active transport^#^ [n=3389]** | **Walking to & from school vs. walking from school [n=2708]** |
| --- | --- | --- | --- | --- | --- |
| Residential density [0-1000] | Total | 75.9 | 76.0 | 367.2 | 80.6 |
|  | Direct | 427.8 | 76.9 | 382.9 | 72.1 |
| Land use mix – diversity^1^ [1-5] | Total | 68.2 | 78.5 | 339.0 | 78.6 |
|  | Direct | **-1040.0** | 75.0 | 322.7 | 90.6 |
| Transit stop proximity [1-5] | Total | 93.8 | 118.5 | 564.9 | 102.7 |
|  | Direct | 555.5 | 122.1 | 578.6 | 104.0 |
| Recreational facilities^2^ [1-5] | Total^a^ | **-19.7** | 57.8 | 209.5 | 73.6 |
| Park proximity [1-5] | Total | 71.0 | 74.1 | 163.6 | 92.7 |
|  | Direct | 72.6 | 77.3 | 238.3 | 92.7 |
| Accessibility & walking facilities [1-4] | Total^a^ | 68.9 | 66.2 | 279.1 | 399.9 |
| Traffic safety [1-4] | Total^a^ | 73.7 | 86.8 | 117.1 | 88.9 |
| Pedestrian infrastructure [1-4] | Total^a^ | 68.9 | 129.1 | 183.6 | 160.5 |
| Safety from crime [1-4] | Total^a^ | 97.1 | 246.9 | 188.6 | 222.6 |
| Aesthetics [1-4] | Total^a^ | 94.8 | 61.4 | 245.2 | 57.4 |
| Buffers between streets & footpath [1-4] | Total | 65.0 | 315.5 | 256.4 | 245.8 |
|  | Direct | 69.4 | 311.8 | 259.7 | 250.5 |
| Parking difficult [1-4] | Total^a^ | **-548.2** | 176.2 | 146.1 | 179.9 |
| Trees [1-4] | Total | 67.9 | 60.1 | 2152.3 | 101.4 |
|  | Direct | 75.2 | 62.9 | 2160.1 | 109.1 |
| Distance to school [1-5] | Total^a^ | 674.9 | 111.6 | **-295.1** | 531.6 |

*Notes.* ^1^ excluding transit stops; ^2^ excluding parks; in bold: effects significant because AIC value of model with interaction term 10+ smaller than that without interaction term; ^#^ N=3389 instead of 3594 because data from Israel and Portugal were excluded from the analyses due to 0% prevalence of cycling to/from school. Analyses undertaken on 20 imputed datasets. ^a^Total and direct effects are equivalent as no mediating variables of characteristic-outcome associations were included in the models. Complete case analyses are in Tables S46, S48, S49 and S50. Model covariates are reported in Table S1.

# **Table S19.** City-specific total and direct effects of parent-perceived neighbourhood recreational facilities and parking on adolescents’ walking to and from school vs. no active transport to/from school (reference category) (2 latent profiles) (multiple imputations; n=5213)

|  | **Recreational facilities^1^** | | | **Parking difficult** | | |
| --- | --- | --- | --- | --- | --- | --- |
| City (country) | ***OR*** | **95% CI** | ***p*** | ***OR*** | **95% CI** | ***p*** |
| *High-income countries* |  |  |  |  |  |  |
| Melbourne (AUS) | 1.36 | 0.73, 2.53 | .338 | 0.98 | 0.64, 1.52 | .940 |
| Ghent (BEL) | 0.81 | 0.50, 1.30 | .378 | 1.23 | 0.79, 1.91 | .368 |
| Hradec Králové (CZE) | 0.97 | 0.50, 1.89 | .936 | 1.30 | 0.64, 2.65 | .475 |
| Olomouc (CZE) | 1.11 | 0.60, 2.05 | .737 | 0.91 | 0.46, 1.78 | .780 |
| Odense (DNK) | **3.58** | **1.15, 11.21** | **.029** | 0.99 | 0.40, 2.46 | .989 |
| Hong Kong (HKG, China) | 1.09 | 0.90, 1.33 | .363 | 0.97 | 0.82, 1.14 | .715 |
| Haifa (ISR) | 0.93 | 0.58, 1.47 | .747 | **1.47** | **1.00, 2.16** | **.048** |
| Various cities (PRT) | 1.13 | 0.72, 1.79 | .589 | 1.67 | 0.87, 3.20 | .123 |
| Valencia (ESP) | 0.91 | 0.60, 1.37 | .644 | 1.10 | 0.80, 1.51 | .546 |
| Baltimore (USA) | 0.95 | 0.65, 1.39 | .795 | **1.47** | **1.02, 2.10** | **.038** |
| Seattle (USA) | 1.33 | 0.89, 2.00 | .163 | 0.97 | 0.68, 1.39 | .879 |
| *Low-middle-income countries* |  |  |  |  |  |  |
| Dhaka (BGD) | 0.56 | 0.24, 1.28 | .171 | 0.56 | 0.25, 1.26 | .158 |
| Curitiba (BRA) | 0.89 | 0.65, 1.22 | .456 | 1.00 | 0.83, 1.21 | .959 |
| Chennai (IND) | 0.91 | 0.51, 1.63 | .752 | 1.01 | 0.79, 1.28 | .951 |
| Kuala Lumpur (MYS) | 1.08 | 0.79, 1.48 | .623 | 0.94 | 0.66, 1.33 | .721 |
| Gombe (NGA) | 0.96 | 0.52, 1.77 | .906 | 0.94 | 0.72, 1.23 | .656 |

*Notes.* ^1^ excluding parks; OR = odd ratio; CI = confidence interval; *p* = *p*-value, in bold: effects significant at *p*<0.05. Complete case analyses are in Table S47. Total and direct effects are equivalent as no mediating variables of characteristic-outcome associations were included in the models.

# **Table S20.** City-specific total and direct effects of parent-perceived distance to school on adolescents’ cycling to/from school vs. no active transport to/from school (reference category) (2 latent profiles) (multiple imputations; n=3389)^#^

|  | **Distance to school** | | |
| --- | --- | --- | --- |
| City (country) | ***OR*** | **95% CI** | ***p*** |
| *High-income countries* |  |  |  |
| Melbourne (AUS) | 0.94 | 0.43, 2.06 | .882 |
| Ghent (BEL) | **0.54** | **0.36, 0.80** | **.003** |
| Hradec Králové (CZE) | 0.66 | 0.25, 1.74 | .402 |
| Olomouc (CZE) | 0.50 | 0.10, 2.43 | .392 |
| Odense (DNK) | **0.29** | **0.17, 0.49** | **<.001** |
| Hong Kong (HKG, China) | **0.65** | **0.45, 0.94** | **.024** |
| Valencia (ESP) | **0.30** | **0.15, 0.59** | **<.001** |
| Baltimore (USA) | 0.91 | 0.36, 2.30 | .849 |
| Seattle (USA) | 0.69 | 0.36, 1.30 | .252 |
| *Low-middle-income countries* |  |  |  |
| Dhaka (BGD) | 0.87 | 0.27, 2.88 | .825 |
| Curitiba (BRA) | 0.60 | 0.32, 1.16 | .129 |
| Chennai (IND) | **0.44** | **0.32, 0.60** | **<.001** |
| Kuala Lumpur (MYS) | **0.65** | **0.42, 1.00** | **.050** |
| Gombe (NGA) | **0.36** | **0.17, 0.78** | **.009** |

*Notes.* OR = odd ratio; CI = confidence interval; *p* = *p*-value, in bold: effects significant at *p*<0.05. ^#^ excluding data from Israel and Portugal due to 0% prevalence of cycling to/from school. Total and direct effects are equivalent as no mediating variables of characteristic-outcome associations were included in the models.

# **COMPLETE CASE ANALYSES**

# **Table S21.** Total and direct effects of perceived neighbourhood environment characteristics on adolescents’ active transport to/from school [complete case analyses; N=4725]

| Model | Environmental effect estimated | ***OR*** | **95% CI** | ***p-value*** |
| --- | --- | --- | --- | --- |
| 1T | Total effects of Residential density | **1.001** | **1.000, 1.001** | **0.004** |
| 1D | Direct effects of Residential density | 1.001 | 0.999, 1.001 | 0.074 |
| 2T | Total effects of Land use mix diversity^1^ | **1.42** | **1.29, 1.56** | **<0.001** |
| 2D | Direct effects of Land use mix diversity^1^ | **1.43** | **1.27, 1.61** | **<0.001** |
| 3T | Total effects of Transit stop proximity | 0.97 | 0.90, 1.04 | 0.357 |
| 3D | Direct effects of Transit stop proximity | 0.96 | 0.89, 1.04 | 0.296 |
| 4T | Total effects of Recreational facilities^2^ | 1.11 | 0.99, 1.25 | 0.079 |
| 4D | Direct effects of Recreational facilities^2^ | 1.11 | 0.99, 1.25 | 0.079 |
| 5T | Total effects of Park proximity | 0.94 | 0.87, 1.01 | 0.102 |
| 5D | Direct effects of Park proximity | 0.93 | 0.86, 1.01 | 0.073 |
| 6T | Total effects of Accessibility and walking facilities | 1.09 | 0.94, 1.27 | 0.259 |
| 6D | Direct effects of Accessibility and walking facilities | 1.09 | 0.94, 1.27 | 0.259 |
| 7T | Total effects of Traffic safety | **1.16** | **1.03, 1.32** | **0.015** |
| 7D | Direct effects of Traffic safety | **1.16** | **1.03, 1.32** | **0.015** |
| 8T | Total effects of Pedestrians infrastructure | **1.14** | **1.01, 1.28** | **0.039** |
| 8D | Direct effects of Pedestrians infrastructure | **1.14** | **1.01, 1.28** | **0.039** |
| 9T | Total effects of Safety from crime | 0.92 | 0.85, 1.01 | 0.087 |
| 9D | Direct effects of Safety from crime | 0.92 | 0.85, 1.01 | 0.087 |
| 10T | Total effects of Aesthetics | 1.00 | 0.90, 1.12 | 0.987 |
| 10D | Direct effects of Aesthetics | 1.00 | 0.90, 1.12 | 0.987 |
| 11T | Total effects of Buffers between street and footpath | 0.99 | 0.91, 1.06 | 0.727 |
| 11D | Direct effects of Buffers between street and footpath | 0.94 | 0.87, 1.02 | 0.135 |
| 12T | Total effects of Parking difficult | 1.05 | 0.98, 1.14 | 0.171 |
| 12D | Direct effects of Parking difficult | 1.05 | 0.98, 1.14 | 0.171 |
| 13T | Total effects of Trees | 0.99 | 0.92, 1.08 | 0.897 |
| 13D | Direct effects of Trees | 0.99 | 0.90, 1.08 | 0.747 |
| 14T | Total effects of Distance to school | **0.36** | **0.34, 0.39** | **<0.001** |
| 14D | Direct effects of Distance to school | **0.36** | **0.34, 0.39** | **<0.001** |

*Notes.* ^1^ excluding transit stops; ^2^ excluding parks; OR=odd ratio; CI = confidence intervals; in bold: effects significant at *p*<0.05. The reference category of the outcome variable is “not engaging in active transport to/from school”.

# **Table S22.** Total and direct effects of perceived neighbourhood environment characteristics on adolescents’ regular cycling to/from school [complete case analyses; N= 4355] (excluding Israel*, Portugal* and Czech Rep-Olomouc)]

| Model | Environmental effect estimated | ***OR*** | **95% CI** | ***p-value*** |
| --- | --- | --- | --- | --- |
| 1T | Total effects of Residential density | 1.00 | 0.998, 1.00 | 0.147 |
| 1D | Direct effects of Residential density | 1.00 | 0.998, 1.00 | 0.302 |
| 2T | Total effects of Land use mix diversity^1^ | 1.07 | 0.89, 1.29 | 0.445 |
| 2D | Direct effects of Land use mix diversity^1^ | 1.10 | 0.87, 1.39 | 0.406 |
| 3T | Total effects of Transit stop proximity | 1.02 | 0.87, 1.21 | 0.795 |
| 3D | Direct effects of Transit stop proximity | 1.02 | 0.86, 1.20 | 0.846 |
| 4T | Total effects of Recreational facilities^2^ | 0.96 | 0.76, 1.22 | 0.761 |
| 4D | Direct effects of Recreational facilities^2^ | 0.96 | 0.76, 1.22 | 0.761 |
| 5T | Total effects of Park proximity | 0.93 | 0.80, 1.07 | 0.308 |
| 5D | Direct effects of Park proximity | 0.91 | 0.79, 1.05 | 0.210 |
| 6T | Total effects of Accessibility and walking facilities | 0.97 | 0.72, 1.30 | 0.829 |
| 6D | Direct effects of Accessibility and walking facilities | 0.97 | 0.72, 1.30 | 0.829 |
| 7T | Total effects of Traffic safety | 1.16 | 0.93, 1.45 | 0.195 |
| 7D | Direct effects of Traffic safety | 1.16 | 0.93, 1.45 | 0.195 |
| 8T | Total effects of Pedestrians infrastructure | 1.03 | 0.82, 1.29 | 0.807 |
| 8D | Direct effects of Pedestrians infrastructure | 1.03 | 0.82, 1.29 | 0.807 |
| 9T | Total effects of Safety from crime | 1.16 | 0.97, 1.38 | 0.108 |
| 9D | Direct effects of Safety from crime | 1.16 | 0.97, 1.38 | 0.108 |
| 10T | Total effects of Aesthetics | 1.20 | 0.98, 1.48 | 0.083 |
| 10D | Direct effects of Aesthetics | 1.20 | 0.98, 1.48 | 0.083 |
| 11T | Total effects of Buffers between street and footpath | 1.03 | 0.89, 1.19 | 0.704 |
| 11D | Direct effects of Buffers between street and footpath | 1.05 | 0.90, 1.23 | 0.507 |
| 12T | Total effects of Parking difficult | 0.96 | 0.84, 1.11 | 0.612 |
| 12D | Direct effects of Parking difficult | 0.96 | 0.84, 1.11 | 0.612 |
| 13T | Total effects of Trees | 0.99 | 0.86, 1.13 | 0.846 |
| 13D | Direct effects of Trees | 0.92 | 0.79, 1.08 | 0.315 |
| 14T | Total effects of Distance to school | 0.93 | 0.83, 1.04 | 0.197 |
| 14D | Direct effects of Distance to school | 0.93 | 0.83, 1.04 | 0.197 |

*Notes.* ^1^ excluding transit stops; ^2^ excluding parks; OR=odd ratio; CI = confidence intervals; in bold: effects significant at *p*<0.05. * No one cycled to/from school. Regular cycling means cycling to/from school 5 to 10 times a week. The reference category of the outcome variable is “not engaging in regular cycling to/from school”.

# **Table S23.** Total and direct effects of perceived neighbourhood environment characteristics on adolescents’ regular walking to/from school [complete case analyses; N=4725]

| Model | Environmental effect estimated | ***OR*** | **95% CI** | ***p-value*** |
| --- | --- | --- | --- | --- |
| 1T | Total effects of Residential density | **1.001** | **1.000, 1.001** | **0.001** |
| 1D | Direct effects of Residential density | 1.001 | 0.999, 1.001 | 0.091 |
| 2T | Total effects of Land use mix diversity^1^ | **1.45** | **1.32, 1.60** | **<0.001** |
| 2D | Direct effects of Land use mix diversity^1^ | **1.48** | **1.32, 1.66** | **<0.001** |
| 3T | Total effects of Transit stop proximity | 0.95 | 0.88, 1.03 | 0.199 |
| 3D | Direct effects of Transit stop proximity | 0.94 | 0.87, 1.02 | 0.151 |
| 4T | Total effects of Recreational facilities^2^ | 1.11 | 0.99, 1.25 | 0.072 |
| 4D | Direct effects of Recreational facilities^2^ | 1.11 | 0.99, 1.25 | 0.072 |
| 5T | Total effects of Park proximity | 0.95 | 0.88, 1.02 | 0.158 |
| 5D | Direct effects of Park proximity | 0.94 | 0.87, 1.02 | 0.141 |
| 6T | Total effects of Accessibility and walking facilities | **1.17** | **1.00, 1.36** | **0.044** |
| 6D | Direct effects of Accessibility and walking facilities | **1.17** | **1.00, 1.36** | **0.044** |
| 7T | Total effects of Traffic safety | 1.06 | 0.94, 1.19 | 0.350 |
| 7D | Direct effects of Traffic safety | 1.06 | 0.94, 1.19 | 0.350 |
| 8T | Total effects of Pedestrians infrastructure | **1.14** | **1.01, 1.28** | **0.038** |
| 8D | Direct effects of Pedestrians infrastructure | **1.14** | **1.01, 1.28** | **0.038** |
| 9T | Total effects of Safety from crime | 0.92 | 0.84, 1.01 | 0.065 |
| 9D | Direct effects of Safety from crime | 0.92 | 0.84, 1.01 | 0.065 |
| 10T | Total effects of Aesthetics | 0.94 | 0.84, 1.05 | 0.253 |
| 10D | Direct effects of Aesthetics | 0.94 | 0.84, 1.05 | 0.253 |
| 11T | Total effects of Buffers between street and footpath | 0.99 | 0.92, 1.07 | 0.821 |
| 11D | Direct effects of Buffers between street and footpath | 0.94 | 0.86, 1.02 | 0.124 |
| 12T | Total effects of Parking difficult | 1.06 | 0.98, 1.14 | 0.147 |
| 12D | Direct effects of Parking difficult | 1.06 | 0.98, 1.14 | 0.147 |
| 13T | Total effects of Trees | 1.03 | 0.95, 1.12 | 0.493 |
| 13D | Direct effects of Trees | 1.03 | 0.94, 1.13 | 0.484 |
| 14T | Total effects of Distance to school | **0.38** | **0.36, 0.41** | **<0.001** |
| 14D | Direct effects of Distance to school | **0.38** | **0.36, 0.41** | **<0.001** |

*Notes.* ^1^ excluding transit stops; ^2^ excluding parks; OR=odd ratio; CI = confidence intervals; in bold: effects significant at *p*<0.05. Regular walking means walking to/from school 5 to 10 times a week. The reference category of the outcome variable is “not engaging in regular walking to/from school”.

# **Table S24.** Total and direct effects of perceived neighbourhood environment characteristics on adolescents’ walking to and from school vs. no active transport to/from school (reference category) (2 latent profiles) [complete case analyses; N=4960]

| Model | Environmental effect estimated | ***OR*** | **95% CI** | ***p-value*** |
| --- | --- | --- | --- | --- |
| 1T | Total effects of Residential density | **1.001** | **1.000, 1.002** | **<0.001** |
| 1D | Direct effects of Residential density | **1.001** | **1.000, 1.001** | **0.040** |
| 2T | Total effects of Land use mix diversity^1^ | **1.55** | **1.39, 1.74** | **<0.001** |
| 2D | Direct effects of Land use mix diversity^1^ | **1.64** | **1.43, 1.88** | **0.001** |
| 3T | Total effects of Transit stop proximity | **0.89** | **0.82, 0.98** | **0.014** |
| 3D | Direct effects of Transit stop proximity | **0.88** | **0.80, 0.97** | **0.008** |
| 4T | Total effects of Recreational facilities^2^ | 1.12 | 0.98, 1.28 | 0.110 |
| 4D | Direct effects of Recreational facilities^2^ | 1.12 | 0.98, 1.28 | 0.110 |
| 5T | Total effects of Park proximity | 0.93 | 0.85, 1.02 | 0.130 |
| 5D | Direct effects of Park proximity | 0.93 | 0.85, 1.01 | 0.090 |
| 6T | Total effects of Accessibility and walking facilities | 1.18 | 0.99, 1.40 | 0.064 |
| 6D | Direct effects of Accessibility and walking facilities | 1.18 | 0.99, 1.40 | 0.064 |
| 7T | Total effects of Traffic safety | **1.16** | **1.01, 1.33** | **0.041** |
| 7D | Direct effects of Traffic safety | **1.16** | **1.01, 1.33** | **0.041** |
| 8T | Total effects of Pedestrians infrastructure | **1.16** | **1.01, 1.33** | **0.036** |
| 8D | Direct effects of Pedestrians infrastructure | **1.16** | **1.01, 1.33** | **0.036** |
| 9T | Tfotal effects of Safety from crime | **0.90** | **0.81, 1.00** | **0.041** |
| 9D | Direct effects of Safety from crime | **0.90** | **0.81, 1.00** | **0.041** |
| 10T | Total effects of Aesthetics | 0.94 | 0.83, 1.07 | 0.339 |
| 10D | Direct effects of Aesthetics | 0.94 | 0.83, 1.07 | 0.339 |
| 11T | Total effects of Buffers between street and footpath | 1.01 | 0.93, 1.11 | 0.773 |
| 11D | Direct effects of Buffers between street and footpath | 0.95 | 0.86, 1.04 | 0.281 |
| 12T | Total effects of Parking difficult | 1.04 | 0.96, 1.14 | 0.317 |
| 12D | Direct effects of Parking difficult | 1.04 | 0.96, 1.14 | 0.317 |
| 13T | Total effects of Trees | 1.04 | 0.95, 1.14 | 0.401 |
| 13D | Direct effects of Trees | 1.03 | 0.93, 1.15 | 0.541 |
| 14T | Total effects of Distance to school | **0.31** | **0.28, 0.34** | **<0.001** |
| 14D | Direct effects of Distance to school | **0.31** | **0.28, 0.34** | **<0.001** |

*Notes.* ^1^ excluding transit stops; ^2^ excluding parks; OR=odd ratio; CI = confidence intervals; in bold: effects significant at *p*<0.05.

# **Table S25.** Total and direct effects of perceived neighbourhood environment characteristics on adolescents’ walking from school vs. no active transport to/from school (reference category) (2 latent profiles) [complete case analyses; N=3518]

| Model | Environmental effect estimated | ***OR*** | **95% CI** | ***p-value*** |
| --- | --- | --- | --- | --- |
| 1T | Total effects of Residential density | 1.001 | 0.999, 1.001 | 0.214 |
| 1D | Direct effects of Residential density | 1.001 | 0.999 1.001 | 0.344 |
| 2T | Total effects of Land use mix diversity^1^ | **1.27** | **1.09, 1.47** | **0.002** |
| 2D | Direct effects of Land use mix diversity^1^ | **1.25** | **1.04, 1.49** | **0.017** |
| 3T | Total effects of Transit stop proximity | 1.04 | 0.93, 1.18 | 0.486 |
| 3D | Direct effects of Transit stop proximity | 1.05 | 0.92, 1.18 | 0.474 |
| 4T | Total effects of Recreational facilities^2^ | 1.14 | 0.95, 1.37 | 0.168 |
| 4D | Direct effects of Recreational facilities^2^ | 1.14 | 0.95, 1.37 | 0.168 |
| 5T | Total effects of Park proximity | 0.94 | 0.83, 1.06 | 0.309 |
| 5D | Direct effects of Park proximity | 0.93 | 0.82, 1.05 | 0.244 |
| 6T | Total effects of Accessibility and walking facilities | 1.17 | 0.92, 1.50 | 0.201 |
| 6D | Direct effects of Accessibility and walking facilities | 1.17 | 0.92, 1.50 | 0.201 |
| 7T | Total effects of Traffic safety | 1.10 | 0.91, 1.34 | 0.329 |
| 7D | Direct effects of Traffic safety | 1.10 | 0.91, 1.34 | 0.329 |
| 8T | Total effects of Pedestrians infrastructure | 1.09 | 0.90, 1.32 | 0.393 |
| 8D | Direct effects of Pedestrians infrastructure | 1.09 | 0.90, 1.32 | 0.393 |
| 9T | Total effects of Safety from crime | 0.90 | 0.76, 1.04 | 0.146 |
| 9D | Direct effects of Safety from crime | 0.90 | 0.76, 1.04 | 0.146 |
| 10T | Total effects of Aesthetics | 1.01 | 0.85, 1.21 | 0.873 |
| 10D | Direct effects of Aesthetics | 1.01 | 0.85, 1.21 | 0.873 |
| 11T | Total effects of Buffers between street and footpath | 1.03 | 0.91, 1.16 | 0.622 |
| 11D | Direct effects of Buffers between street and footpath | 0.98 | 0.86, 1.11 | 0.721 |
| 12T | Total effects of Parking | 1.03 | 0.91, 1.16 | 0.668 |
| 12D | Direct effects of Parking | 1.03 | 0.91, 1.16 | 0.668 |
| 13T | Total effects of Trees | 1.02 | 0.89, 1.16 | 0.806 |
| 13D | Direct effects of Trees | 0.99 | 0.85, 1.15 | 0.902 |
| 14T | Total effects of Distance to school | **0.52** | **0.47, 0.58** | **<0.001** |
| 14D | Direct effects of Distance to school | **0.52** | **0.47, 0.58** | **<0.001** |

*Notes.* ^1^ excluding transit stops; ^2^ excluding parks; OR=odd ratio; CI = confidence intervals; in bold: effects significant at *p*<0.05.

# **Table S26.** Total and direct effects of perceived neighbourhood environment characteristics on adolescents’ cycling to and from school vs. no active transport to/from school (reference category) (2 latent classes) [complete case analyses; N=3389]

| Model | Environmental effect estimated | ***OR*** | **95% CI** | ***p-value*** |
| --- | --- | --- | --- | --- |
| 1T | Total effects of Residential density | 0.999 | 0.998, 1.001 | 0.342 |
| 1D | Direct effects of Residential density | 0.999 | 0.998, 1.001 | 0.522 |
| 2T | Total effects of Land use mix diversity^1^ | 1.24 | 1.00, 1.53 | 0.054 |
| 2D | Direct effects of Land use mix diversity^1^ | 1.26 | 0.95, 1.68 | 0.114 |
| 3T | Total effects of Transit stop proximity | 1.00 | 0.83, 1.21 | 0.976 |
| 3D | Direct effects of Transit stop proximity | 0.99 | 0.81, 1.20 | 0.901 |
| 4T | Total effects of Recreational facilities^2^ | 0.92 | 0.69, 1.23 | 0.590 |
| 4D | Direct effects of Recreational facilities^2^ | 0.92 | 0.69, 1.23 | 0.590 |
| 5T | Total effects of Park proximity | 0.90 | 0.76, 1.07 | 0.252 |
| 5D | Direct effects of Park proximity | 0.89 | 0.74, 1.06 | 0.177 |
| 6T | Total effects of Accessibility and walking facilities | 1.00 | 0.71, 1.42 | 0.987 |
| 6D | Direct effects of Accessibility and walking facilities | 1.00 | 0.71, 1.42 | 0.987 |
| 7T | Total effects of Traffic safety | 1.15 | 0.88, 1.50 | 0.301 |
| 7D | Direct effects of Traffic safety | 1.15 | 0.88, 1.50 | 0.301 |
| 8T | Total effects of Pedestrians infrastructure | 1.12 | 0.86, 1.45 | 0.405 |
| 8D | Direct effects of Pedestrians infrastructure | 1.12 | 0.86, 1.45 | 0.405 |
| 9T | Total effects of Safety from crime | 1.05 | 0.86, 1.30 | 0.620 |
| 9D | Direct effects of Safety from crime | 1.05 | 0.86, 1.30 | 0.620 |
| 10T | Total effects of Aesthetics | 1.25 | 0.97, 1.60 | 0.087 |
| 10D | Direct effects of Aesthetics | 1.25 | 0.97, 1.60 | 0.087 |
| 11T | Total effects of Buffers between street and footpath | 1.00 | 0.84, 1.19 | 0.978 |
| 11D | Direct effects of Buffers between street and footpath | 1.00 | 0.82, 1.21 | 0.972 |
| 12T | Total effects of Parking | 0.96 | 0.81, 1.14 | 0.620 |
| 12D | Direct effects of Parking | 0.96 | 0.81, 1.14 | 0.620 |
| 13T | Total effects of Trees | 0.99 | 0.84, 1.17 | 0.942 |
| 13D | Direct effects of Trees | 0.94 | 0.78, 1.13 | 0.508 |
| 14T | Total effects of Distance to school | **0.47** | **0.40, 0.55** | **<0.001** |
| 14D | Direct effects of Distance to school | **0.47** | **0.40, 0.55** | **<0.001** |

*Notes.* ^1^ excluding transit stops; ^2^ excluding parks; OR=odd ratio; CI = confidence intervals; in bold: effects significant at *p*<0.05.

# **Table S27.** Total and direct effects of perceived neighbourhood environment characteristics on adolescents’ walking to and from school vs. walking from school (reference category) (2 latent profiles) [complete case analyses; N=2604]

| Model | Environmental effect estimated | ***OR*** | **95% CI** | ***p-value*** |
| --- | --- | --- | --- | --- |
| 1T | Total effects of Residential density | 1.001 | 0.999, 1.002 | 0.128 |
| 1D | Direct effects of Residential density | 1.001 | 0.999, 1.002 | 0.238 |
| 2T | Total effects of Land use mix diversity^1^ | **1.28** | **1.08, 1.52** | **0.005** |
| 2D | Direct effects of Land use mix diversity^1^ | **1.31** | **1.07, 1.61** | **0.010** |
| 3T | Total effects of Transit stop proximity | 0.90 | 0.78, 1.03 | 0.130 |
| 3D | Direct effects of Transit stop proximity | 0.89 | 0.77, 1.02 | 0.095 |
| 4T | Total effects of Recreational facilities^2^ | 1.03 | 0.84, 1.27 | 0.774 |
| 4D | Direct effects of Recreational facilities^2^ | 1.03 | 0.84, 1.27 | 0.774 |
| 5T | Total effects of Park proximity | 1.00 | 0.87, 1.15 | 0.995 |
| 5D | Direct effects of Park proximity | 0.99 | 0.86, 1.14 | 0.936 |
| 6T | Total effects of Accessibility and walking facilities | 1.03 | 0.78, 1.35 | 0.857 |
| 6D | Direct effects of Accessibility and walking facilities | 1.03 | 0.78, 1.35 | 0.857 |
| 7T | Total effects of Traffic safety | 1.11 | 0.90, 1.37 | 0.333 |
| 7D | Direct effects of Traffic safety | 1.11 | 0.90, 1.37 | 0.333 |
| 8T | Total effects of Pedestrians infrastructure | 1.10 | 0.88, 1.37 | 0.392 |
| 8D | Direct effects of Pedestrians infrastructure | 1.10 | 0.88, 1.37 | 0.392 |
| 9T | Total effects of Safety from crime | 1.01 | 0.86, 1.18 | 0.887 |
| 9D | Direct effects of Safety from crime | 1.01 | 0.86, 1.18 | 0.887 |
| 10T | Total effects of Aesthetics | 0.96 | 0.79, 1.16 | 0.651 |
| 10D | Direct effects of Aesthetics | 0.96 | 0.79, 1.16 | 0.651 |
| 11T | Total effects of Buffers between street and footpath | 1.04 | 0.91, 1.20 | 0.568 |
| 11D | Direct effects of Buffers between street and footpath | 1.02 | 0.88, 1.18 | 0.772 |
| 12T | Total effects of Parking difficult | 1.05 | 0.92, 1.20 | 0.506 |
| 12D | Direct effects of Parking difficult | 1.05 | 0.92, 1.20 | 0.506 |
| 13T | Total effects of Trees | 1.02 | 0.88, 1.18 | 0.828 |
| 13D | Direct effects of Trees | 0.99 | 0.84, 1.16 | 0.910 |
| 14T | Total effects of Distance to school | **0.56** | **0.50, 0.63** | **<0.001** |
| 14D | Direct effects of Distance to school | **0.56** | **0.50, 0.63** | **<0.001** |

*Notes.* ^1^ excluding transit stops; ^2^ excluding parks; OR=odd ratio; CI = confidence intervals; in bold: effects significant at *p*<0.05.

# **Table S28.** Distance to school as a moderator of total and direct effects of perceived neighbourhood environment characteristics on adolescents’ active transport to/from school [complete case analyses]

| Model | Environmental effect estimated | Regression coefficient | ***OR*** | **95% CI** | ***p-value*** |
| --- | --- | --- | --- | --- | --- |
| 1T | Total effects of Residential density | Interaction with Distance to school | 1.00 | 0.99, 1.00 | 0.312 |
| 1D | Direct effects of Residential density | Interaction with Distance to school | 1.00 | 0.99, 1.00 | 0.263 |
| 2T | Total effects of Land use mix diversity^1^ | Interaction with Distance to school | **0.87** | **0.80, 0.95** | **0.002** |
|  |  | 1-5 minutes | **1.83** | **1.37, 2.43** | **<0.001** |
|  |  | 6-10 minutes | **1.60** | **1.30, 1.96** | **<0.001** |
|  |  | 11-20 minutes | **1.40** | **1.21, 1.60** | **<0.001** |
|  |  | 21-30 minutes | **1.22** | **1.10, 1.35** | **<0.001** |
|  |  | 31+ minutes | 1.07 | 0.94, 1.22 | 0.333 |
| 2D | Direct effects of Land use mix diversity^1^ | Interaction with Distance to school | **0.87** | **0.80, 0.96** | **0.003** |
|  |  | 1-5 minutes | **1.81** | **1.35, 2.44** | **<0.001** |
|  |  | 6-10 minutes | **1.59** | **1.27, 1.98** | **<0.001** |
|  |  | 11-20 minutes | **1.39** | **1.18, 1.62** | **<0.001** |
|  |  | 21-30 minutes | **1.21** | **1.07, 1.38** | **0.003** |
|  |  | 31+ minutes | 1.06 | 0.91, 1.23 | 0.457 |
| 3T | Total effects of Transit stop proximity | Interaction with Distance to school | 0.95 | 0.89, 1.01 | 0.076 |
| 3D | Direct effects of Transit stop proximity | Interaction with Distance to school | **0.94** | **0.88, 1.00** | **0.045** |
|  |  | 1-5 minutes | 1.22 | 1.00, 1.50 | 0.053 |
|  |  | 6-10 minutes | 1.15 | 0.99, 1.33 | 0.072 |
|  |  | 11-20 minutes | 1.08 | 0.97, 1.20 | 0.171 |
|  |  | 21-30 minutes | 1.01 | 0.93, 1.10 | 0.840 |
|  |  | 31+ minutes | 0.95 | 0.85, 1.05 | 0.312 |
| 4T | Total effects of Recreational facilities^2^ | Interaction with Distance to school | 0.99 | 0.91, 1.08 | 0.830 |
| 4D | Direct effects of Recreational facilities^2^ | Interaction with Distance to school | 0.99 | 0.91, 1.08 | 0.830 |
| 5T | Total effects of Park proximity | Interaction with Distance to school | **0.92** | **0.86, 0.97** | **0.004** |
|  |  | 1-5 minutes | 1.19 | 0.98, 1.46 | 0.082 |
|  |  | 6-10 minutes | 1.09 | 0.94, 1.27 | 0.231 |
|  |  | 11-20 minutes | 1.00 | 0.90, 1.11 | 0.953 |
|  |  | 21-30 minutes | 0.92 | 0.85, 1.00 | 0.051 |
|  |  | 31+ minutes | **0.84** | **0.76, 0.93** | **0.001** |
| 5D | Direct effects of Park proximity | Interaction with Distance to school | **0.92** | **0.86, 0.98** | **0.005** |
|  |  | 1-5 minutes | 1.18 | 0.96, 1.44 | 0.109 |
|  |  | 6-10 minutes | 1.08 | 0.93, 1.26 | 0.299 |
|  |  | 11-20 minutes | 0.99 | 0.89, 1.10 | 0.897 |
|  |  | 21-30 minutes | **0.91** | **0.84, 0.99** | **0.034** |
|  |  | 31+ minutes | **0.84** | **0.75, 0.93** | **<0.001** |
| 6T | Total effects of Accessibility and walking facilities | Interaction with Distance to school | **0.80** | **0.70, 0.91** | **<0.001** |
|  |  | 1-5 minutes | **2.00** | **1.32, 3.04** | **0.001** |
|  |  | 6-10 minutes | **1.60** | **1.18, 2.17** | **0.003** |
|  |  | 11-20 minutes | **1.28** | **1.03, 1.57** | **0.023** |
|  |  | 21-30 minutes | 1.02 | 0.87, 1.20 | 0.824 |
|  |  | 31+ minutes | **0.81** | **0.66, 1.00** | **0.045** |
| 6D | Direct effects of Accessibility and walking facilities | Interaction with Distance to school | **0.80** | **0.70, 0.91** | **<0.001** |
|  |  | 1-5 minutes | **2.00** | **1.32, 3.04** | **0.001** |
|  |  | 6-10 minutes | **1.60** | **1.18, 2.17** | **0.003** |
|  |  | 11-20 minutes | **1.28** | **1.03, 1.57** | **0.023** |
|  |  | 21-30 minutes | 1.02 | 0.87, 1.20 | 0.824 |
|  |  | 31+ minutes | **0.81** | **0.66, 1.00** | **0.045** |
| 7T | Total effects of Traffic safety | Interaction with Distance to school | 1.04 | 0.94, 1.16 | 0.440 |
| 7D | Direct effects of Traffic safety | Interaction with Distance to school | 1.04 | 0.94, 1.16 | 0.440 |
| 8T | Total effects of Pedestrians infrastructure | Interaction with Distance to school | **0.87** | **0.78, 0.97** | **0.011** |
|  |  | 1-5 minutes | **1.77** | **1.24, 2.51** | **0.002** |
|  |  | 6-10 minutes | **1.53** | **1.19, 1.98** | **<0.001** |
|  |  | 11-20 minutes | **1.33** | **1.12, 1.58** | **0.001** |
|  |  | 21-30 minutes | **1.16** | **1.02, 1.32** | **0.027** |
|  |  | 31+ minutes | 1.01 | 0.85, 1.19 | 0.925 |
| 8D | Direct effects of Pedestrians infrastructure | Interaction with Distance to school | **0.87** | **0.78, 0.97** | **0.011** |
|  |  | 1-5 minutes | **1.77** | **1.24, 2.51** | **0.002** |
|  |  | 6-10 minutes | **1.53** | **1.19, 1.98** | **<0.001** |
|  |  | 11-20 minutes | **1.33** | **1.12, 1.58** | **0.001** |
|  |  | 21-30 minutes | **1.16** | **1.02, 1.32** | **0.027** |
|  |  | 31+ minutes | 1.01 | 0.85, 1.19 | 0.925 |
| 9T | Total effects of Safety from crime | Interaction with Distance to school | 0.96 | 0.89, 1.04 | 0.328 |
| 9D | Direct effects of Safety from crime | Interaction with Distance to school | 0.96 | 0.89, 1.04 | 0.328 |
| 10T | Total effects of Aesthetics | Interaction with Distance to school | 1.04 | 0.95, 1.14 | 0.368 |
| 10D | Direct effects of Aesthetics | Interaction with Distance to school | 1.04 | 0.95, 1.14 | 0.368 |
| 11T | Total effects of Buffers between street and footpath | Interaction with Distance to school | 1.04 | 0.97, 1.11 | 0.295 |
| 11D | Direct effects of Buffers between street and footpath | Interaction with Distance to school | 1.04 | 0.97, 1.11 | 0.304 |
| 12T | Total effects of Parking difficult | Interaction with Distance to school | 1.00 | 0.93, 1.07 | 0.989 |
| 12D | Direct effects of Parking difficult | Interaction with Distance to school | 1.00 | 0.93, 1.07 | 0.989 |
| 13T | Total effects of Trees | Interaction with Distance to school | 0.97 | 0.90, 1.04 | 0.344 |
| 13D | Direct effects of Trees | Interaction with Distance to school | 0.97 | 0.90, 1.04 | 0.342 |

*Notes.* ^1^ excluding transit stops; ^2^ excluding parks. OR = odd ratio; CI = confidence interval; in bold: effects significant at *p*<0.05. The reference category of the outcome variable is “not engaging in active transport to/from school”.

.

# **Table S29.** Distance to school as a moderator of total and direct effects of perceived neighbourhood environment characteristics on adolescents’ regular cycling to/from school (excluding Israel, Portugal and Czech Rep-Olomouc) [complete case analyses]

| Model | Environmental effect estimated | Regression coefficient | ***OR*** | **95% CI** | ***p-value*** |
| --- | --- | --- | --- | --- | --- |
| 1T | Total effects of Residential density | Interaction with Distance to school | 1.00 | 0.999, 1.001 | 0.510 |
| 1D | Direct effects of Residential density | Interaction with Distance to school | 1.00 | 0.999, 1.001 | 0.469 |
| 2T | Total effects of Land use mix diversity^1^ | Interaction with Distance to school | **1.21** | **1.07, 1.37** | **0.003** |
|  |  | 1-5 minutes | **0.62** | **0.43, 0.91** | **0.014** |
|  |  | 6-10 minutes | **0.76** | **0.57, 1.00** | **0.048** |
|  |  | 11-20 minutes | 0.92 | 0.75, 1.12 | 0.400 |
|  |  | 21-30 minutes | 1.11 | 0.91, 1.35 | 0.292 |
|  |  | 31+ minutes | **1.35** | **1.04, 1.74** | **0.023** |
| 2D | Direct effects of Land use mix diversity^1^ | Interaction with Distance to school | **1.22** | **1.07, 1.38** | **0.002** |
|  |  | 1-5 minutes | **0.64** | **0.43, 0.96** | **0.032** |
|  |  | 6-10 minutes | 0.79 | 0.58, 1.07 | 0.124 |
|  |  | 11-20 minutes | 0.96 | 0.75, 1.23 | 0.728 |
|  |  | 21-30 minutes | 1.17 | 0.91, 1.49 | 0.222 |
|  |  | 31+ minutes | **1.42** | **1.05, 1.92** | **0.024** |
| 3T | Total effects of Transit stop proximity | Interaction with Distance to school | 1.08 | 0.98, 1.20 | 0.119 |
| 3D | Direct effects of Transit stop proximity | Interaction with Distance to school | 1.09 | 0.99, 1.21 | 0.094 |
| 4T | Total effects of Recreational facilities^2^ | Interaction with Distance to school | 1.10 | 0.99, 1.23 | 0.087 |
| 4D | Direct effects of Recreational facilities^2^ | Interaction with Distance to school | 1.10 | 0.99, 1.23 | 0.087 |
| 5T | Total effects of Park proximity | Interaction with Distance to school | 1.01 | 0.93, 1.09 | 0.867 |
| 5D | Direct effects of Park proximity | Interaction with Distance to school | 1.01 | 0.93, 1.10 | 0.751 |
| 6T | Total effects of Accessibility and walking facilities | Interaction with Distance to school | **1.20** | **1.00, 1.43** | **0.045** |
|  |  | 1-5 minutes | 0.60 | 0.35, 1.05 | 0.075 |
|  |  | 6-10 minutes | 0.72 | 0.48, 1.10 | 0.127 |
|  |  | 11-20 minutes | 0.86 | 0.63, 1.19 | 0.370 |
|  |  | 21-30 minutes | 1.03 | 0.77, 1.40 | 0.822 |
|  |  | 31+ minutes | 1.24 | 0.58, 1.80 | 0.260 |
| 6D | Direct effects of Accessibility and walking facilities | Interaction with Distance to school | **1.20** | **1.00, 1.43** | **0.045** |
|  |  | 1-5 minutes | 0.60 | 0.35, 1.05 | 0.075 |
|  |  | 6-10 minutes | 0.72 | 0.48, 1.10 | 0.127 |
|  |  | 11-20 minutes | 0.86 | 0.63, 1.19 | 0.370 |
|  |  | 21-30 minutes | 1.03 | 0.77, 1.40 | 0.822 |
|  |  | 31+ minutes | 1.24 | 0.85, 1.80 | 0.260 |
| 7T | Total effects of Traffic safety | Interaction with Distance to school | 1.13 | 0.98, 1.31 | 0.101 |
| 7D | Direct effects of Traffic safety | Interaction with Distance to school | 1.13 | 0.98, 1.31 | 0.101 |
| 8T | Total effects of Pedestrians infrastructure | Interaction with Distance to school | 1.11 | 0.96, 1.29 | 0.153 |
| 8D | Direct effects of Pedestrians infrastructure | Interaction with Distance to school | 1.11 | 0.96, 1.29 | 0.153 |
| 9T | Total effects of Safety from crime | Interaction with Distance to school | 1.02 | 0.91, 1.15 | 0.714 |
| 9D | Direct effects of Safety from crime | Interaction with Distance to school | 1.02 | 0.91, 1.15 | 0.714 |
| 10T | Total effects of Aesthetics | Interaction with Distance to school | 1.03 | 0.91, 1.15 | 0.677 |
| 10D | Direct effects of Aesthetics | Interaction with Distance to school | 1.03 | 0.91, 1.15 | 0.677 |
| 11T | Total effects of Buffers between street and footpath | Interaction with Distance to school | 0.94 | 0.86, 1.03 | 0.216 |
| 11D | Direct effects of Buffers between street and footpath | Interaction with Distance to school | 0.95 | 0.87, 1.04 | 0.291 |
| 12T | Total effects of Parking difficult | Interaction with Distance to school | 1.02 | 0.93, 1.12 | 0.724 |
| 12D | Direct effects of Parking difficult | Interaction with Distance to school | 1.02 | 0.93, 1.12 | 0.724 |
| 13T | Total effects of Trees | Interaction with Distance to school | 1.07 | 0.97, 1.17 | 0.166 |
| 13D | Direct effects of Trees | Interaction with Distance to school | 1.07 | 0.97, 1.17 | 0.164 |

*Notes.* ^1^ excluding transit stops; ^2^ excluding parks; OR = odd ratio; CI = confidence interval; in bold: effects significant at *p*<0.05. Regular cycling means cycling to/from school 5 to 10 times a week. The reference category of the outcome variable is “not engaging in regular cycling to/from school”.

# **Table S30.** Distance to school as a moderator of total and direct effects of perceived neighbourhood environment characteristics on adolescents’ regular walking to/from school [complete case analyses]

| Model | Environmental effect estimated | Regression coefficient | ***OR*** | **95% CI** | ***p-value*** |
| --- | --- | --- | --- | --- | --- |
| 1T | Total effects of Residential density | Interaction with Distance to school | **0.9996** | **0.9993, 0.9999** | **0.017** |
|  |  | 1-5 minutes | **1.002** | **1.0007, 1.003** | **0.001** |
|  |  | 6-10 minutes | **1.001** | **1.0005, 1.002** | **0.001** |
|  |  | 11-20 minutes | **1.001** | **1.0003, 1.0015** | **0.003** |
|  |  | 21-30 minutes | 1.0005 | 0.999, 1.001 | 0.075 |
|  |  | 31+ minutes | 1.0002 | 0.999, 1.001 | 0.662 |
| 1D | Direct effects of Residential density | Interaction with Distance to school | **0.996** | **0.9993, 0.9999** | **0.017** |
|  |  | 1-5 minutes | **1.001** | **1.0005, 1.002** | **0.004** |
|  |  | 6-10 minutes | **1.001** | **1.0003, 1.002** | **0.005** |
|  |  | 11-20 minutes | **1.001** | **1.0001, 1.001** | **0.024** |
|  |  | 21-30 minutes | 1.000 | 0.9997, 1.001 | 0.286 |
|  |  | 31+ minutes | **0.999** | 0.9992, 1.001 | 0.900 |
| 2T | Total effects of Land use mix diversity^1^ | Interaction with Distance to school | **0.89** | **0.82, 0.96** | **0.003** |
|  |  | 1-5 minutes | **1.64** | **1.31, 2.06** | **<0.001** |
|  |  | 6-10 minutes | **1.46** | **1.24, 1.71** | **<0.001** |
|  |  | 11-20 minutes | **1.29** | **1.16, 1.45** | **<0.001** |
|  |  | 21-30 minutes | **1.15** | **1.03, 1.28** | **0.015** |
|  |  | 31+ minutes | 1.02 | 0.87, 1.19 | 0.820 |
| 2D | Direct effects of Land use mix diversity^1^ | Interaction with Distance to school | **0.88** | **0.81, 0.95** | **0.002** |
|  |  | 1-5 minutes | **1.71** | **1.35, 2.18** | **<0.001** |
|  |  | 6-10 minutes | **1.51** | **1.26, 1.80** | **<0.001** |
|  |  | 11-20 minutes | **1.32** | **1.16, 1.52** | **<0.001** |
|  |  | 21-30 minutes | **1.16** | **1.02, 1.33** | **0.025** |
|  |  | 31+ minutes | 1.02 | 0.86, 1.22 | 0.794 |
| 3T | Total effects of Transit stop proximity | Interaction with Distance to school | 0.97 | 0.92, 1.03 | 0.335 |
| 3D | Direct effects of Transit stop proximity | Interaction with Distance to school | 0.96 | 0.91, 1.02 | 0.197 |
| 4T | Total effects of Recreational facilities^2^ | Interaction with Distance to school | 0.94 | 0.87, 1.01 | 0.084 |
| 4D | Direct effects of Recreational facilities^2^ | Interaction with Distance to school | 0.94 | 0.87, 1.01 | 0.084 |
| 5T | Total effects of Park proximity | Interaction with Distance to school | **0.92** | **0.87, 0.97** | **0.003** |
|  |  | 1-5 minutes | 1.13 | 0.96, 1.32 | 0.136 |
|  |  | 6-10 minutes | 1.04 | 0.92, 1.16 | 0.537 |
|  |  | 11-20 minutes | 0.95 | 0.87, 1.04 | 0.302 |
|  |  | 21-30 minutes | **0.88** | **0.80, 0.96** | **0.005** |
|  |  | 31+ minutes | **0.81** | **0.72, 0.91** | **0.001** |
| 5D | Direct effects of Park proximity | Interaction with Distance to school | **0.92** | **0.87, 0.97** | **0.002** |
|  |  | 1-5 minutes | 1.13 | 0.96, 1.32 | 0.141 |
|  |  | 6-10 minutes | 1.03 | 0.92, 1.16 | 0.564 |
|  |  | 11-20 minutes | 0.95 | 0.87, 1.04 | 0.276 |
|  |  | 21-30 minutes | **0.87** | **0.80, 0.96** | **0.004** |
|  |  | 31+ minutes | **0.80** | **0.71, 0.91** | **<0.001** |
| 6T | Total effects of Accessibility and walking facilities | Interaction with Distance to school | **0.83** | **0.74, 0.93** | **0.002** |
|  |  | 1-5 minutes | **1.76** | **1.26, 2.48** | **0.001** |
|  |  | 6-10 minutes | **1.47** | **1.15, 1.87** | **0.002** |
|  |  | 11-20 minutes | **1.22** | **1.02, 1.46** | **0.027** |
|  |  | 21-30 minutes | 1.02 | 0.86, 1.21 | 0.856 |
|  |  | 31+ minutes | 0.85 | 0.67, 1.07 | 0.157 |
| 6D | Direct effects of Accessibility and walking facilities | Interaction with Distance to school | **0.83** | **0.74, 0.93** | **0.002** |
|  |  | 1-5 minutes | **1.76** | **1.26, 2.48** | **0.001** |
|  |  | 6-10 minutes | **1.47** | **1.15, 1.87** | **0.002** |
|  |  | 11-20 minutes | **1.22** | **1.02, 1.46** | **0.027** |
|  |  | 21-30 minutes | 1.02 | 0.86, 1.21 | 0.856 |
|  |  | 31+ minutes | 0.85 | 0.67, 1.07 | 0.157 |
| 7T | Total effects of Traffic safety | Interaction with Distance to school | 0.97 | 0.89, 1.07 | 0.597 |
| 7D | Direct effects of Traffic safety | Interaction with Distance to school | 0.97 | 0.89, 1.07 | 0.597 |
| 8T | Total effects of Pedestrians infrastructure | Interaction with Distance to school | 0.92 | 0.84, 1.02 | 0.102 |
| 8D | Direct effects of Pedestrians infrastructure | Interaction with Distance to school | 0.92 | 0.84, 1.02 | 0.102 |
| 9T | Total effects of Safety from crime | Interaction with Distance to school | 0.97 | 0.91, 1.05 | 0.461 |
| 9D | Direct effects of Safety from crime | Interaction with Distance to school | 0.97 | 0.91, 1.05 | 0.461 |
| 10T | Total effects of Aesthetics | Interaction with Distance to school | 0.98 | 0.91, 1.06 | 0.679 |
| 10D | Direct effects of Aesthetics | Interaction with Distance to school | 0.98 | 0.91, 1.06 | 0.679 |
| 11T | Total effects of Buffers between street and footpath | Interaction with Distance to school | 0.99 | 0.93, 1.05 | 0.700 |
| 11D | Direct effects of Buffers between street and footpath | Interaction with Distance to school | 0.99 | 0.93, 1.05 | 0.633 |
| 12T | Total effects of Parking difficult | Interaction with Distance to school | 0.99 | 0.93, 1.06 | 0.853 |
| 12D | Direct effects of Parking difficult | Interaction with Distance to school | 0.99 | 0.93, 1.06 | 0.853 |
| 13T | Total effects of Trees | Interaction with Distance to school | **0.91** | **0.85, 0.96** | **0.002** |
|  |  | 1-5 minutes | **1.29** | **1.08, 1.55** | **0.005** |
|  |  | 6-10 minutes | **1.17** | **1.03, 1.33** | **0.015** |
|  |  | 11-20 minutes | 1.06 | 0.97, 1.16 | 0.211 |
|  |  | 21-30 minutes | 0.96 | 0.87, 1.06 | 0.408 |
|  |  | 31+ minutes | **0.87** | **0.76, 0.99** | **0.039** |
| 13D | Direct effects of Trees | Interaction with Distance to school | **0.91** | **0.85, 0.97** | **0.005** |
|  |  | 1-5 minutes | **1.28** | **1.07, 1.54** | **0.008** |
|  |  | 6-10 minutes | **1.17** | **1.02, 1.34** | **0.022** |
|  |  | 11-20 minutes | 1.07 | 0.96, 1.18 | 0.218 |
|  |  | 21-30 minutes | 0.97 | 0.88, 1.08 | 0.589 |
|  |  | 31+ minutes | 0.89 | 0.77, 1.02 | 0.089 |

*Notes.* ^1^ excluding transit stops; ^2^ excluding parks; OR = odd ratio; CI = confidence interval; in bold: effects significant at *p*<0.05. Regular walking means walking to/from school 5 to 10 times a week. The reference category of the outcome variable is “not engaging in regular walking to/from school”.

# **Table S31.** Distance to school as a moderator of total and direct effects of perceived neighbourhood environment characteristics on adolescents’ walking to and from school vs. no active transport to from school (reference category) (2 latent profiles) [complete case analyses]

| Model | Environmental effect estimated | Regression coefficient | ***OR*** | **95% CI** | ***p-value*** |
| --- | --- | --- | --- | --- | --- |
| 1T | Total effects of Residential density | Interaction with Distance to school | 0.999 | 0.999, 1.000 | 0.651 |
| 1D | Direct effects of Residential density | Interaction with Distance to school | 0.999 | 0.999, 1.000 | 0.485 |
| 2T | Total effects of Land use mix diversity^1^ | Interaction with Distance to school | **0.89** | **0.81, 0.98** | **0.023** |
|  |  | 1-5 minutes | **1.66** | **1.25, 2.20** | **<0.001** |
|  |  | 6-10 minutes | **1.48** | **1.21, 1.81** | **<0.001** |
|  |  | 11-20 minutes | **1.32** | **1.15, 1.52** | **<0.001** |
|  |  | 21-30 minutes | **1.18** | **1.03, 1.35** | **0.016** |
|  |  | 31+ minutes | 1.05 | 0.87, 1.27 | 0.599 |
| 2D | Direct effects of Land use mix diversity^1^ | Interaction with Distance to school | **0.89** | **0.81, 0.98** | **0.023** |
|  |  | 1-5 minutes | **1.72** | **1.28, 2.32** | **<0.001** |
|  |  | 6-10 minutes | **1.53** | **1.23, 1.91** | **<0.001** |
|  |  | 11-20 minutes | **1.37** | **1.16, 1.61** | **<0.001** |
|  |  | 21-30 minutes | **1.22** | **1.03, 1.43** | **0.018** |
|  |  | 31+ minutes | 1.08 | 0.88, 1.34 | 0.469 |
| 3T | Total effects of Transit stop proximity | Interaction with Distance to school | 0.99 | 0.92, 1.07 | 0.841 |
| 3D | Direct effects of Transit stop proximity | Interaction with Distance to school | 0.99 | 0.92, 1.06 | 0.745 |
| 4T | Total effects of Recreational facilities^2^ | Interaction with Distance to school | 1.01 | 0.92, 1.11 | 0.825 |
| 4D | Direct effects of Recreational facilities^2^ | Interaction with Distance to school | 1.01 | 0.92, 1.11 | 0.825 |
| 5T | Total effects of Park proximity | Interaction with Distance to school | 0.95 | 0.89, 1.02 | 0.149 |
| 5D | Direct effects of Park proximity | Interaction with Distance to school | 0.95 | 0.89, 1.02 | 0.154 |
| 6T | Total effects of Accessibility and walking facilities | Interaction with Distance to school | **0.84** | **0.73, 0.96** | **0.013** |
|  |  | 1-5 minutes | **1.77** | **1.18, 2.66** | **0.006** |
|  |  | 6-10 minutes | **1.48** | **1.10, 1.98** | **0.009** |
|  |  | 11-20 minutes | **1.24** | **1.00, 1.52** | **0.047** |
|  |  | 21-30 minutes | 1.03 | 0.84, 1.27 | 0.751 |
|  |  | 31+ minutes | 0.86 | 0.65, 1.15 | 0.315 |
| 6D | Direct effects of Accessibility and walking facilities | Interaction with Distance to school | **0.84** | **0.73, 0.96** | **0.013** |
|  |  | 1-5 minutes | **1.77** | **1.18, 2.66** | **0.006** |
|  |  | 6-10 minutes | **1.48** | **1.10, 1.98** | **0.009** |
|  |  | 11-20 minutes | **1.24** | **1.00, 1.52** | **0.047** |
|  |  | 21-30 minutes | 1.03 | 0.84, 1.27 | 0.751 |
|  |  | 31+ minutes | 0.86 | 0.65, 1.15 | 0.315 |
| 7T | Total effects of Traffic safety | Interaction with Distance to school | 1.07 | 0.94, 1.20 | 0.306 |
| 7D | Direct effects of Traffic safety | Interaction with Distance to school | 1.07 | 0.94, 1.20 | 0.306 |
| 8T | Total effects of Pedestrians infrastructure | Interaction with Distance to school | 0.91 | 0.81, 1.03 | 0.154 |
| 8D | Direct effects of Pedestrians infrastructure | Interaction with Distance to school | 0.91 | 0.81, 1.03 | 0.154 |
| 9T | Total effects of Safety from crime | Interaction with Distance to school | 0.98 | 0.89, 1.07 | 0.589 |
| 9D | Direct effects of Safety from crime | Interaction with Distance to school | 0.98 | 0.89, 1.07 | 0.589 |
| 10T | Total effects of Aesthetics | Interaction with Distance to school | 1.03 | 0.93, 1.14 | 0.585 |
| 10D | Direct effects of Aesthetics | Interaction with Distance to school | 1.03 | 0.93, 1.14 | 0.585 |
| 11T | Total effects of Buffers between street and footpath | Interaction with Distance to school | 1.06 | 0.98, 1.14 | 0.134 |
| 11D | Direct effects of Buffers between street and footpath | Interaction with Distance to school | 1.05 | 0.98, 1.14 | 0.168 |
| 12T | Total effects of Parking difficult | Interaction with Distance to school | 0.98 | 0.91, 1.06 | 0.606 |
| 12D | Direct effects of Parking difficult | Interaction with Distance to school | 0.98 | 0.91, 1.06 | 0.606 |
| 13T | Total effects of Trees | Interaction with Distance to school | 0.94 | 0.87, 1.02 | 0.137 |
| 13D | Direct effects of Trees | Interaction with Distance to school | 0.95 | 0.87, 1.02 | 0.174 |

*Notes.* ^1^ excluding transit stops; ^2^ excluding parks; OR = odd ratio; CI = confidence interval; LPA = latent profile analyses; in bold: effects significant at *p*<0.05

# **Table S32.** Distance to school as a moderator of total and direct effects of perceived neighbourhood environment characteristics on adolescents’ walking from school vs. no active transport to/from school (reference category) (2 latent profiles) [complete case analyses]

| Model | Environmental effect estimated | Regression coefficient | ***OR*** | **95% CI** | ***p-value*** |
| --- | --- | --- | --- | --- | --- |
| 1T | Total effects of Residential density | Interaction with Distance to school | 1.000 | 0.999, 1.000 | 0.488 |
| 1D | Direct effects of Residential density | Interaction with Distance to school | 1.000 | 0.999, 1.000 | 0.430 |
| 2T | Total effects of Land use mix diversity^1^ | Interaction with Distance to school | 0.95 | 0.84, 1.07 | 0.420 |
| 2D | Direct effects of Land use mix diversity^1^ | Interaction with Distance to school | 0.94 | 0.83, 1.07 | 0.356 |
| 3T | Total effects of Transit stop proximity | Interaction with Distance to school | 0.96 | 0.87, 1.06 | 0.408 |
| 3D | Direct effects of Transit stop proximity | Interaction with Distance to school | 0.95 | 0.86, 1.05 | 0.292 |
| 4T | Total effects of Recreational facilities^2^ | Interaction with Distance to school | 0.94 | 0.84, 1.06 | 0.346 |
| 4D | Direct effects of Recreational facilities^2^ | Interaction with Distance to school | 0.94 | 0.84, 1.06 | 0.346 |
| 5T | Total effects of Park proximity | Interaction with Distance to school | 0.94 | 0.86, 1.02 | 0.138 |
| 5D | Direct effects of Park proximity | Interaction with Distance to school | 0.94 | 0.86, 1.02 | 0.144 |
| 6T | Total effects of Accessibility and walking facilities | Interaction with Distance to school | **0.80** | **0.66, 0.97** | **0.020** |
|  |  | 1-5 minutes | **2.19** | **1.20, 3.99** | **0.011** |
|  |  | 6-10 minutes | **1.74** | **1.13, 2.70** | **0.013** |
|  |  | 11-20 minutes | **1.39** | **1.03, 1.89** | **0.034** |
|  |  | 21-30 minutes | 1.11 | 0.86, 1.44 | 0.427 |
|  |  | 31+ minutes | 0.89 | 0.63, 1.24 | 0.476 |
| 6D | Direct effects of Accessibility and walking facilities | Interaction with Distance to school | **0.80** | **0.66, 0.97** | **0.020** |
|  |  | 1-5 minutes | **2.19** | **1.20, 3.99** | **0.011** |
|  |  | 6-10 minutes | **1.74** | **1.13, 2.70** | **0.013** |
|  |  | 11-20 minutes | **1.39** | **1.03, 1.89** | **0.034** |
|  |  | 21-30 minutes | 1.11 | 0.86, 1.44 | 0.427 |
|  |  | 31+ minutes | 0.89 | 0.63, 1.24 | 0.476 |
| 7T | Total effects of Traffic safety | Interaction with Distance to school | 0.93 | 0.79, 1.09 | 0.385 |
| 7D | Direct effects of Traffic safety | Interaction with Distance to school | 0.93 | 0.79, 1.09 | 0.385 |
| 8T | Total effects of Pedestrians infrastructure | Interaction with Distance to school | 0.88 | 0.75, 1.03 | 0.112 |
| 8D | Direct effects of Pedestrians infrastructure | Interaction with Distance to school | 0.88 | 0.75, 1.03 | 0.112 |
| 9T | Total effects of Safety from crime | Interaction with Distance to school | **0.87** | **0.78, 0.98** | **0.019** |
|  |  | 1-5 minutes | 1.32 | 0.93, 1.88 | 0.116 |
|  |  | 6-10 minutes | 1.15 | 0.90, 1.48 | 0.269 |
|  |  | 11-20 minutes | 1.00 | 0.84, 1.19 | 0.975 |
|  |  | 21-30 minutes | 0.87 | 0.75, 1.02 | 0.087 |
|  |  | 31+ minutes | **0.76** | **0.62, 0.94** | **0.011** |
| 9D | Direct effects of Safety from crime | Interaction with Distance to school | **0.87** | **0.78, 0.98** | **0.019** |
|  |  | 1-5 minutes | 1.32 | 0.93, 1.88 | 0.116 |
|  |  | 6-10 minutes | 1.15 | 0.90, 1.48 | 0.269 |
|  |  | 11-20 minutes | 1.00 | 0.84, 1.19 | 0.975 |
|  |  | 21-30 minutes | 0.87 | 0.75, 1.02 | 0.087 |
|  |  | 31+ minutes | **0.76** | **0.62, 0.94** | **0.011** |
| 10T | Total effects of Aesthetics | Interaction with Distance to school | 0.95 | 0.84, 1.07 | 0.406 |
| 10D | Direct effects of Aesthetics | Interaction with Distance to school | 0.95 | 0.84, 1.07 | 0.406 |
| 11T | Total effects of Buffers between street and footpath | Interaction with Distance to school | 0.99 | 0.91, 1.09 | 0.895 |
| 11D | Direct effects of Buffers between street and footpath | Interaction with Distance to school | 0.99 | 0.90, 1.09 | 0.835 |
| 12T | Total effects of Parking difficult | Interaction with Distance to school | 1.01 | 0.92, 1.12 | 0.774 |
| 12D | Direct effects of Parking difficult | Interaction with Distance to school | 1.01 | 0.92, 1.12 | 0.774 |
| 13T | Total effects of Trees | Interaction with Distance to school | 0.94 | 0.84, 1.05 | 0.246 |
| 13D | Direct effects of Trees | Interaction with Distance to school | 0.93 | 0.83, 1.04 | 0.228 |

*Notes.* ^1^ excluding transit stops; ^2^ excluding parks; OR = odd ratio; CI = confidence interval; in bold: effects significant at *p*<0.05.

.

# **Table S33.** Proximity to school as a moderator of total and direct effects of perceived neighbourhood environment characteristics on adolescents’ cycling to and from school vs. no active transport to/from school (reference category) (2 latent classes) [complete case analyses]

| Model | Environmental effect estimated | Regression coefficient | ***OR*** | **95% CI** | ***p-value*** |
| --- | --- | --- | --- | --- | --- |
| 1T | Total effects of Residential density | Interaction with Distance to school | 0.999 | 0.999, 1.000 | 0.283 |
| 1D | Direct effects of Residential density | Interaction with Distance to school | 0.999 | 0.999, 1.000 | 0.329 |
| 2T | Total effects of Land use mix diversity^1^ | Interaction with Distance to school | 0.89 | 0.75, 1.06 | 0.205 |
| 2D | Direct effects of Land use mix diversity^1^ | Interaction with Distance to school | 0.92 | 0.77, 1.10 | 0.382 |
| 3T | Total effects of Transit stop proximity | Interaction with Distance to school | 0.99 | 0.87, 1.14 | 0.908 |
| 3D | Direct effects of Transit stop proximity | Interaction with Distance to school | 1.00 | 0.87, 1.15 | 0.994 |
| 4T | Total effects of Recreational facilities^2^ | Interaction with Distance to school | 0.96 | 0.82, 1.13 | 0.622 |
| 4D | Direct effects of Recreational facilities^2^ | Interaction with Distance to school | 0.96 | 0.82, 1.13 | 0.622 |
| 5T | Total effects of Park proximity | Interaction with Distance to school | 0.93 | 0.82, 1.04 | 0.202 |
| 5D | Direct effects of Park proximity | Interaction with Distance to school | 0.94 | 0.83, 1.06 | 0.311 |
| 6T | Total effects of Accessibility and walking facilities | Interaction with Distance to school | 0.99 | 0.77, 1.28 | 0.931 |
| 6D | Direct effects of Accessibility and walking facilities | Interaction with Distance to school | 0.99 | 0.77, 1.28 | 0.931 |
| 7T | Total effects of Traffic safety | Interaction with Distance to school | 1.23 | 0.99, 1.53 | 0.066 |
| 7D | Direct effects of Traffic safety | Interaction with Distance to school | 1.23 | 0.99, 1.53 | 0.066 |
| 8T | Total effects of Pedestrians infrastructure | Interaction with Distance to school | 1.10 | 0.90, 1.36 | 0.355 |
| 8D | Direct effects of Pedestrians infrastructure | Interaction with Distance to school | 1.10 | 0.90, 1.36 | 0.355 |
| 9T | Total effects of Safety from crime | Interaction with Distance to school | 0.92 | 0.77, 1.09 | 0.308 |
| 9D | Direct effects of Safety from crime | Interaction with Distance to school | 0.92 | 0.77, 1.09 | 0.308 |
| 10T | Total effects of Aesthetics | Interaction with Distance to school | 1.03 | 0.87, 1.22 | 0.745 |
| 10D | Direct effects of Aesthetics | Interaction with Distance to school | 1.03 | 0.87, 1.22 | 0.745 |
| 11T | Total effects of Buffers between street and footpath | Interaction with Distance to school | 1.03 | 0.90, 1.18 | 0.699 |
| 11D | Direct effects of Buffers between street and footpath | Interaction with Distance to school | 1.06 | 0.92, 1.21 | 0.434 |
| 12T | Total effects of Parking difficult | Interaction with Distance to school | 0.92 | 0.80, 1.05 | 0.226 |
| 12D | Direct effects of Parking difficult | Interaction with Distance to school | 0.92 | 0.80, 1.05 | 0.226 |
| 13T | Total effects of Trees | Interaction with Distance to school | 1.06 | 0.93, 1.21 | 0.363 |
| 13D | Direct effects of Trees | Interaction with Distance to school | 1.07 | 0.93, 1.22 | 0.350 |

*Notes.* ^1^ excluding transit stops; ^2^ excluding parks; OR = odd ratio; CI = confidence interval; in bold: effects significant at *p*<0.05.

# **Table S34.** Distance to school as a moderator of total and direct effects of perceived neighbourhood environment characteristics on adolescents’ walking to and from school vs. walking from school (reference category) (2 latent profiles) [complete case analyses]

| Model | Environmental effect estimated | Regression coefficient | ***OR*** | **95% CI** | ***p-value*** |
| --- | --- | --- | --- | --- | --- |
| 1T | Total effects of Residential density | Interaction with Distance to school | 0.999 | 0.999, 1.000 | 0.716 |
| 1D | Direct effects of Residential density | Interaction with Distance to school | 0.999 | 0.999, 1.000 | 0.633 |
| 2T | Total effects of Land use mix diversity^1^ | Interaction with Distance to school | 0.97 | 0.85, 1.11 | 0.680 |
| 2D | Direct effects of Land use mix diversity^1^ | Interaction with Distance to school | 0.98 | 0.85, 1.12 | 0.754 |
| 3T | Total effects of Transit stop proximity | Interaction with Distance to school | 1.02 | 0.92, 1.14 | 0.652 |
| 3D | Direct effects of Transit stop proximity | Interaction with Distance to school | 1.01 | 0.91, 1.12 | 0.846 |
| 4T | Total effects of Recreational facilities^2^ | Interaction with Distance to school | 1.06 | 0.94, 1.20 | 0.333 |
| 4D | Direct effects of Recreational facilities^2^ | Interaction with Distance to school | 1.06 | 0.94, 1.20 | 0.333 |
| 5T | Total effects of Park proximity | Interaction with Distance to school | 1.05 | 0.96, 1.15 | 0.262 |
| 5D | Direct effects of Park proximity | Interaction with Distance to school | 1.06 | 0.96, 1.16 | 0.250 |
| 6T | Total effects of Accessibility and walking facilities | Interaction with Distance to school | 1.04 | 0.85, 1.26 | 0.726 |
| 6D | Direct effects of Accessibility and walking facilities | Interaction with Distance to school | 1.04 | 0.85, 1.26 | 0.726 |
| 7T | Total effects of Traffic safety | Interaction with Distance to school | **1.19** | **1.02, 1.40** | **0.031** |
|  |  | 1-5 minutes | 0.73 | 0.48, 1.10 | 0.130 |
|  |  | 6-10 minutes | 0.86 | 0.64, 1.16 | 0.331 |
|  |  | 11-20 minutes | 1.03 | 0.83, 1.29 | 0.788 |
|  |  | 21-30 minutes | 1.23 | 0.96, 1.58 | 0.105 |
|  |  | 31+ minutes | **1.46** | **1.03, 2.09** | **0.035** |
| 7D | Direct effects of Traffic safety | Interaction with Distance to school | **1.19** | **1.02, 1.40** | **0.031** |
|  |  | 1-5 minutes | 0.73 | 0.48, 1.10 | 0.130 |
|  |  | 6-10 minutes | 0.86 | 0.64, 1.16 | 0.331 |
|  |  | 11-20 minutes | 1.03 | 0.83, 1.29 | 0.788 |
|  |  | 21-30 minutes | 1.23 | 0.96, 1.58 | 0.105 |
|  |  | 31+ minutes | **1.46** | **1.03, 2.09** | **0.035** |
| 8T | Total effects of Pedestrians infrastructure | Interaction with Distance to school | 1.03 | 0.87, 1.21 | 0.767 |
| 8D | Direct effects of Pedestrians infrastructure | Interaction with Distance to school | 1.03 | 0.87, 1.21 | 0.767 |
| 9T | Total effects of Safety from crime | Interaction with Distance to school | **1.14** | **1.01, 1.28** | **0.036** |
|  |  | 1-5 minutes | 0.78 | 0.57, 1.06 | 0.111 |
|  |  | 6-10 minutes | 0.88 | 0.71, 1.10 | 0.266 |
|  |  | 11-20 minutes | 1.01 | 0.85, 1.18 | 0.943 |
|  |  | 21-30 minutes | 1.14 | 0.95, 1.38 | 0.158 |
|  |  | 31+ minutes | 1.30 | 1.00, 1.70 | 0.054 |
| 9D | Direct effects of Safety from crime | Interaction with Distance to school | **1.14** | **1.01, 1.28** | **0.036** |
|  |  | 1-5 minutes | 0.78 | 0.57, 1.06 | 0.111 |
|  |  | 6-10 minutes | 0.88 | 0.71, 1.10 | 0.266 |
|  |  | 11-20 minutes | 1.01 | 0.85, 1.18 | 0.943 |
|  |  | 21-30 minutes | 1.14 | 0.95, 1.38 | 0.158 |
|  |  | 31+ minutes | 1.30 | 1.00, 1.70 | 0.054 |
| 10T | Total effects of Aesthetics | Interaction with Distance to school | 1.14 | 1.00, 1.30 | 0.051 |
| 10D | Direct effects of Aesthetics | Interaction with Distance to school | 1.14 | 1.00, 1.30 | 0.051 |
| 11T | Total effects of Buffers between street and footpath | Interaction with Distance to school | **1.20** | **1.08, 1.32** | **<0.001** |
|  |  | 1-5 minutes | **0.71** | **0.55, 0.93** | **0.011** |
|  |  | 6-10 minutes | 0.85 | 0.71, 1.03 | 0.091 |
|  |  | 11-20 minutes | 1.02 | 0.88, 1.18 | 0.807 |
|  |  | 21-30 minutes | **1.22** | **1.03, 1.44** | **0.023** |
|  |  | 31+ minutes | **1.45** | **1.15, 1.85** | **0.002** |
| 11D | Direct effects of Buffers between street and footpath | Interaction with Distance to school | **1.20** | **1.08, 1.33** | **<0.001** |
|  |  | 1-5 minutes | **0.72** | **0.55, 0.94** | **0.015** |
|  |  | 6-10 minutes | 0.86 | 0.71, 1.04 | 0.127 |
|  |  | 11-20 minutes | 1.03 | 0.88, 1.20 | 0.702 |
|  |  | 21-30 minutes | **1.23** | **1.03, 1.47** | **0.021** |
|  |  | 31+ minutes | **1.48** | **1.15, 1.89** | **0.002** |
| 12T | Total effects of Parking difficult | Interaction with Distance to school | 0.95 | 0.85, 1.05 | 0.332 |
| 12D | Direct effects of Parking difficult | Interaction with Distance to school | 0.95 | 0.85, 1.05 | 0.332 |
| 13T | Total effects of Trees | Interaction with Distance to school | 1.09 | 0.97, 1.22 | 0.140 |
| 13D | Direct effects of Trees | Interaction with Distance to school | 1.10 | 0.98, 1.23 | 0.102 |

*Notes.* ^1^ excluding transit stops; ^2^ excluding parks; OR = odd ratio; CI = confidence interval; in bold: effects significant at *p*<0.05.

# **Table S35.** Adolescents’ sex as a moderator of total and direct effects of perceived neighbourhood environment characteristics on adolescents’ active transport to/from school [complete case analyses]

| Model | Environmental effect estimated | Regression coefficient | ***OR*** | **95% CI** | ***p-value*** |
| --- | --- | --- | --- | --- | --- |
| 1T | Total effects of Residential density | Interaction with sex | 1.00 | 0.99, 1.00 | 0.405 |
| 1D | Direct effects of Residential density | Interaction with sex | 1.00 | 0.99, 1.00 | 0.808 |
| 2T | Total effects of Land use mix diversity^1^ | Interaction with sex | 1.07 | 0.91, 1.27 | 0.406 |
| 2D | Direct effects of Land use mix diversity^1^ | Interaction with sex | 1.08 | 0.91, 1.28 | 0.398 |
| 3T | Total effects of Transit stop proximity | Interaction with sex | 1.02 | 0.90, 1.15 | 0.759 |
| 3D | Direct effects of Transit stop proximity | Interaction with sex | 1.02 | 0.90, 1.16 | 0.712 |
| 4T | Total effects of Recreational facilities^2^ | Interaction with sex | 1.11 | 0.94, 1.31 | 0.238 |
| 4D | Direct effects of Recreational facilities^2^ | Interaction with sex | 1.11 | 0.94, 1.31 | 0.238 |
| 5T | Total effects of Park proximity | Interaction with sex | 1.08 | 0.96, 1.21 | 0.195 |
| 5D | Direct effects of Park proximity | Interaction with sex | 1.08 | 0.96, 1.21 | 0.189 |
| 6T | Total effects of Accessibility and walking facilities | Interaction with sex | 1.21 | 0.94, 1.56 | 0.139 |
| 6D | Direct effects of Accessibility and walking facilities | Interaction with sex | 1.21 | 0.94, 1.56 | 0.139 |
| 7T | Total effects of Traffic safety | Interaction with sex | 1.09 | 0.88, 1.35 | 0.450 |
| 7D | Direct effects of Traffic safety | Interaction with sex | 1.09 | 0.88, 1.35 | 0.450 |
| 8T | Total effects of Pedestrians infrastructure | Interaction with sex | 1.16 | 0.94, 1.44 | 0.174 |
| 8D | Direct effects of Pedestrians infrastructure | Interaction with sex | 1.16 | 0.94, 1.44 | 0.174 |
| 9T | Total effects of Safety from crime | Interaction with sex | 1.03 | 0.88, 1.21 | 0.675 |
| 9D | Direct effects of Safety from crime | Interaction with sex | 1.03 | 0.88, 1.21 | 0.675 |
| 10T | Total effects of Aesthetics | Interaction with sex | 1.14 | 0.96, 1.36 | 0.136 |
| 10D | Direct effects of Aesthetics | Interaction with sex | 1.14 | 0.96, 1.36 | 0.136 |
| 11T | Total effects of Buffers between street and footpath | Interaction with sex | 1.07 | 0.94, 1.22 | 0.317 |
| 11D | Direct effects of Buffers between street and footpath | Interaction with sex | 1.06 | 0.93, 1.21 | 0.390 |
| 12T | Total effects of Parking difficult | Interaction with sex | 0.96 | 0.83, 1.09 | 0.506 |
| 12D | Direct effects of Parking difficult | Interaction with sex | 0.96 | 0.83, 1.09 | 0.506 |
| 13T | Total effects of Trees | Interaction with sex | 1.09 | 0.94, 1.25 | 0.260 |
| 13D | Direct effects of Trees | Interaction with sex | 1.08 | 0.93, 1.25 | 0.310 |
| 14T | Total effects of Distance to school | Interaction with sex | 0.88 | 0.76, 1.02 | 0.083 |
| 14D | Direct effects of Distance to school | Interaction with sex | 0.88 | 0.76, 1.02 | 0.083 |

*Notes.* ^1^ excluding transit stops; ^2^ excluding parks. OR = odd ratio; CI = confidence interval; in bold: effects significant at *p*<0.05. The reference category of the outcome variable is “not engaging in active transport to/from school”.

# **Table S36.** Adolescents’ sex as a moderator of total and direct effects of perceived neighbourhood environment characteristics on adolescents’ regular cycling to/from school (excluding Israel, Portugal and Czech Rep-Olomouc) [complete case analyses]

| Model | Environmental effect estimated | Regression coefficient | ***OR*** | **95% CI** | ***p-value*** |  |  |
| --- | --- | --- | --- | --- | --- | --- | --- |
| 1T | Total effects of Residential density | Interaction with sex | 1.00 | 0.99, 1.002 | 0.437 |  |  |
| 1D | Direct effects of Residential density | Interaction with sex | 1.00 | 0.99, 1.002 | 0.524 |  |  |
| 2T | Total effects of Land use mix diversity^1^ | Interaction with sex | **1.43** | **1.04, 1.98** | **0.029** |  |  |
|  |  | Males | 0.91 | 0.72, 1.15 | 0.450 |  |  |
|  |  | Females | **1.31** | **1.01, 1.69** | **0.039** |  |  |
| 2D | Direct effects of Land use mix diversity^1^ | Interaction with sex | **1.43** | **1.03, 1.98** | **0.035** |  |  |
|  |  | Males | 0.94 | 0.71, 1.24 | 0.660 |  |  |
|  |  | Females | 1.34 | 1.00, 1.80 | 0.052 |  |  |
| 3T | Total effects of Transit stop proximity | Interaction with sex | 1.11 | 0.84, 1.47 | 0.454 |  |  |
| 3D | Direct effects of Transit stop proximity | Interaction with sex | 1.10 | 0.83, 1.47 | 0.495 |  |  |
| 4T | Total effects of Recreational facilities^2^ | Interaction with sex | **1.68** | **1.25, 2.28** | **<0.001** |  |  |
|  |  | Males | **0.75** | **0.57, 0.99** | **0.040** |  |  |
|  |  | Females | 1.26 | 0.95, 1.67 | 0.111 |  |  |
| 4D | Direct effects of Recreational facilities^2^ | Interaction with sex | **1.68** | **1.25, 2.28** | **<0.001** |  |  |
|  |  | Males | **0.75** | **0.57, 0.99** | **0.040** |  |  |
|  |  | Females | 1.26 | 0.95, 1.67 | 0.111 |  |  |
| 5T | Total effects of Park proximity | Interaction with sex | **1.28** | **1.02, 1.59** | **0.029** |  |  |
|  |  | Males | **0.82** | **0.68, 0.98** | **0.032** |  |  |
|  |  | Females | 1.05 | 0.87, 1.25 | 0.630 |  |  |
| 5D | Direct effects of Park proximity | Interaction with sex | **1.27** | **1.02, 1.58** | **0.033** |  |  |
|  |  | Males | **0.81** | **0.67, 0.97** | **0.022** |  |  |
|  |  | Females | 1.02 | 0.85, 1.23 | 0.801 |  |  |
| 6T | Total effects of Accessibility and walking facilities | Interaction with sex | **2.02** | **1.26, 3.22** | **0.003** |  |  |
|  |  | Males | 0.70 | 0.49, 1.01 | 0.055 |  |  |
|  |  | Females | 1.41 | 0.96, 2.09 | 0.082 |  |  |
| 6D | Direct effects of Accessibility and walking facilities | Interaction with sex | **2.02** | **1.26, 3.22** | **0.003** |  |  |
|  |  | Males | 0.70 | 0.49, 1.01 | 0.055 |  |  |
|  |  | Females | 1.41 | 0.96, 2.09 | 0.082 |  |  |
| 7T | Total effects of Traffic safety | Interaction with sex | 1.07 | 0.72, 1.61 | 0.730 |  |  |
| 7D | Direct effects of Traffic safety | Interaction with sex | 1.07 | 0.72, 1.61 | 0.730 |  |  |
| 8T | Total effects of Pedestrians infrastructure | Interaction with sex | 1.09 | 0.72, 1.64 | 0.678 |  |  |
| 8D | Direct effects of Pedestrians infrastructure | Interaction with sex | 1.09 | 0.72, 1.64 | 0.678 |  |  |
| 9T | Total effects of Safety from crime | Interaction with sex | 1.19 | 0.86, 1.62 | 0.290 |  |  |
| 9D | Direct effects of Safety from crime | Interaction with sex | 1.19 | 0.86, 1.62 | 0.290 |  |  |
| 10T | Total effects of Aesthetics | Interaction with sex | 1.18 | 0.86, 1.62 | 0.292 |  |  |
| 10D | Direct effects of Aesthetics | Interaction with sex | 1.18 | 0.86, 1.62 | 0.292 |  |  |
| 11T | Total effects of Buffers between street and footpath | Interaction with sex | 1.01 | 0.79, 1.31 | 0.918 |  |  |
| 11D | Direct effects of Buffers between street and footpath | Interaction with sex | 1.00 | 0.78, 1.29 | 0.997 |  |  |
| 12T | Total effects of Parking difficult | Interaction with sex | 1.20 | 0.94, 1.54 | 0.143 |  |  |
| 12D | Direct effects of Parking difficult | Interaction with sex | 1.20 | 0.94, 1.54 | 0.143 |  |  |
| 13T | Total effects of Trees | Interaction with sex | 1.24 | 0.96, 1.59 | 0.096 |  |  |
| 13D | Direct effects of Trees | Interaction with sex | 1.23 | 0.96, 1.59 | 0.108 |  |  |
| 14T | Total effects of Distance to school | Interaction with sex | 0.91 | 0.74, 1.11 | 0.346 |  |  |
| 14D | Direct effects of Distance to school | Interaction with sex | 0.91 | 0.74, 1.11 | 0.346 |  |  |

*Notes.* ^1^ excluding transit stops; ^2^ excluding parks; OR = odd ratio; CI = confidence interval; in bold: effects significant at *p*<0.05. Regular cycling means cycling to/from school 5 to 10 times a week. The reference category of the outcome variable is “not engaging in regular cycling to/from school”.

# **Table S37.** Adolescents’ sex as a moderator of total and direct effects of perceived neighbourhood environment characteristics on adolescents’ regular walking to/from school [complete case analyses]

| Model | Environmental effect estimated | Regression coefficient | ***OR*** | **95% CI** | ***p-value*** |
| --- | --- | --- | --- | --- | --- |
| 1T | Total effects of Residential density | Interaction with sex | 1.00 | 0.99, 1.00 | 0.726 |
| 1D | Direct effects of Residential density | Interaction with sex | 1.00 | 0.99, 1.00 | 0.548 |
| 2T | Total effects of Land use mix diversity^1^ | Interaction with sex | 1.17 | 0.99, 1.39 | 0.069 |
| 2D | Direct effects of Land use mix diversity^1^ | Interaction with sex | 1.18 | 0.99, 1.40 | 0.062 |
| 3T | Total effects of Transit stop proximity | Interaction with sex | 1.05 | 0.93, 1.20 | 0.412 |
| 3D | Direct effects of Transit stop proximity | Interaction with sex | 1.06 | 0.94, 1.21 | 0.342 |
| 4T | Total effects of Recreational facilities^2^ | Interaction with sex | 1.02 | 0.87, 1.20 | 0.813 |
| 4D | Direct effects of Recreational facilities^2^ | Interaction with sex | 1.02 | 0.87, 1.20 | 0.813 |
| 5T | Total effects of Park proximity | Interaction with sex | 1.05 | 0.94, 1.18 | 0.402 |
| 5D | Direct effects of Park proximity | Interaction with sex | 1.05 | 0.94, 1.18 | 0.379 |
| 6T | Total effects of Accessibility and walking facilities | Interaction with sex | 1.06 | 0.82, 1.36 | 0.663 |
| 6D | Direct effects of Accessibility and walking facilities | Interaction with sex | 1.06 | 0.82, 1.36 | 0.663 |
| 7T | Total effects of Traffic safety | Interaction with sex | 0.99 | 0.80, 1.23 | 0.952 |
| 7D | Direct effects of Traffic safety | Interaction with sex | 0.99 | 0.80, 1.23 | 0.952 |
| 8T | Total effects of Pedestrians infrastructure | Interaction with sex | 1.00 | 0.81, 1.24 | 0.994 |
| 8D | Direct effects of Pedestrians infrastructure | Interaction with sex | 1.00 | 0.81, 1.24 | 0.994 |
| 9T | Total effects of Safety from crime | Interaction with sex | 1.04 | 0.89, 1.21 | 0.611 |
| 9D | Direct effects of Safety from crime | Interaction with sex | 1.04 | 0.89, 1.21 | 0.611 |
| 10T | Total effects of Aesthetics | Interaction with sex | 1.01 | 0.85, 1.20 | 0.931 |
| 10D | Direct effects of Aesthetics | Interaction with sex | 1.01 | 0.85, 1.20 | 0.931 |
| 11T | Total effects of Buffers between street and footpath | Interaction with sex | 0.96 | 0.84, 1.09 | 0.498 |
| 11D | Direct effects of Buffers between street and footpath | Interaction with sex | 0.95 | 0.84, 1.09 | 0.471 |
| 12T | Total effects of Parking difficult | Interaction with sex | 0.97 | 0.85, 1.11 | 0.650 |
| 12D | Direct effects of Parking difficult | Interaction with sex | 0.97 | 0.85, 1.11 | 0.650 |
| 13T | Total effects of Trees | Interaction with sex | 0.93 | 0.81, 1.07 | 0.331 |
| 13D | Direct effects of Trees | Interaction with sex | 0.94 | 0.81, 1.08 | 0.354 |
| 14T | Total effects of Distance to school | Interaction with sex | 0.91 | 0.80, 1.03 | 0.132 |
| 14D | Direct effects of Distance to school | Interaction with sex | 0.91 | 0.80, 1.03 | 0.132 |

*Notes.* ^1^ excluding transit stops; ^2^ excluding parks; OR = odd ratio; CI = confidence interval; in bold: effects significant at *p*<0.05. Regular walking means walking to/from school 5 to 10 times a week. The reference category of the outcome variable is “not engaging in regular walking to/from school”.

# **Table S38.** Adolescents’ sex as a moderator of total and direct effects of perceived neighbourhood environment characteristics on adolescents’ walking to and from school vs. no active transport to/from school (reference category) (2 latent profiles) [complete case analyses]

| Model | Environmental effect estimated | Regression coefficient | ***OR*** | **95% CI** | ***p-value*** |
| --- | --- | --- | --- | --- | --- |
| 1T | Total effects of Residential density | Interaction with sex | 1.000 | 0.999, 1.001 | 0.249 |
| 1D | Direct effects of Residential density | Interaction with sex | 1.000 | 0.999, 1.001 | 0.720 |
| 2T | Total effects of Land use mix diversity^1^ | Interaction with sex | 1.19 | 0.97, 1.45 | 0.094 |
| 2D | Direct effects of Land use mix diversity^1^ | Interaction with sex | 1.21 | 0.98, 1.48 | 0.074 |
| 3T | Total effects of Transit stop proximity | Interaction with sex | 0.98 | 0.85, 1.14 | 0.815 |
| 3D | Direct effects of Transit stop proximity | Interaction with sex | 0.99 | 0.85, 1.15 | 0.855 |
| 4T | Total effects of Recreational facilities^2^ | Interaction with sex | 1.06 | 0.88, 1.29 | 0.543 |
| 4D | Direct effects of Recreational facilities^2^ | Interaction with sex | 1.06 | 0.88, 1.29 | 0.543 |
| 5T | Total effects of Park proximity | Interaction with sex | 1.09 | 0.95, 1.24 | 0.207 |
| 5D | Direct effects of Park proximity | Interaction with sex | 1.09 | 0.95, 1.24 | 0.217 |
| 6T | Total effects of Accessibility and walking facilities | Interaction with sex | 1.06 | 0.79, 1.42 | 0.697 |
| 6D | Direct effects of Accessibility and walking facilities | Interaction with sex | 1.06 | 0.79, 1.42 | 0.697 |
| 7T | Total effects of Traffic safety | Interaction with sex | 1.01 | 0.79, 1.30 | 0.909 |
| 7D | Direct effects of Traffic safety | Interaction with sex | 1.01 | 0.79, 1.30 | 0.909 |
| 8T | Total effects of Pedestrians infrastructure | Interaction with sex | 0.99 | 0.77, 1.27 | 0.912 |
| 8D | Direct effects of Pedestrians infrastructure | Interaction with sex | 0.99 | 0.77, 1.27 | 0.912 |
| 9T | Total effects of Safety from crime | Interaction with sex | 0.95 | 0.79, 1.13 | 0.532 |
| 9D | Direct effects of Safety from crime | Interaction with sex | 0.95 | 0.79, 1.13 | 0.532 |
| 10T | Total effects of Aesthetics | Interaction with sex | 1.04 | 0.85, 1.27 | 0.727 |
| 10D | Direct effects of Aesthetics | Interaction with sex | 1.04 | 0.85, 1.27 | 0.727 |
| 11T | Total effects of Buffers between street and footpath | Interaction with sex | 1.01 | 0.87, 1.17 | 0.914 |
| 11D | Direct effects of Buffers between street and footpath | Interaction with sex | 0.99 | 0.85, 1.15 | 0.919 |
| 12T | Total effects of Parking difficult | Interaction with sex | 1.11 | 0.95, 1.30 | 0.185 |
| 12D | Direct effects of Parking difficult | Interaction with sex | 1.11 | 0.95, 1.30 | 0.185 |
| 13T | Total effects of Trees | Interaction with sex | 0.99 | 0.84, 1.16 | 0.896 |
| 13D | Direct effects of Trees | Interaction with sex | 0.97 | 0.82, 1.15 | 0.722 |
| 14T | Total effects of Distance to school | Interaction with sex | 0.90 | 0.77, 1.06 | 0.215 |
| 14D | Direct effects of Distance to school | Interaction with sex | 0.90 | 0.77, 1.06 | 0.215 |

*Notes.* ^1^ excluding transit stops; ^2^ excluding parks; OR = odd ratio; CI = confidence interval; in bold: effects significant at *p*<0.05.

# **Table S39.** Adolescents’ sex as a moderator of total and direct effects of perceived neighbourhood environment characteristics on adolescents’ walking from school vs. no active transport to/from school (reference category) (2 latent profiles) [complete case analyses]

| Model | Environmental effect estimated | Regression coefficient | ***OR*** | **95% CI** | ***p-value*** |
| --- | --- | --- | --- | --- | --- |
| 1T | Total effects of Residential density | Interaction with sex | 0.999 | 0.998, 1.00 | 0.191 |
| 1D | Direct effects of Residential density | Interaction with sex | 0.999 | 0.998, 1.00 | 0.089 |
| 2T | Total effects of Land use mix diversity^1^ | Interaction with sex | 0.92 | 0.70, 1.20 | 0.522 |
| 2D | Direct effects of Land use mix diversity^1^ | Interaction with sex | 0.92 | 0.70, 1.21 | 0.547 |
| 3T | Total effects of Transit stop proximity | Interaction with sex | 1.00 | 0.82, 1.23 | 0.964 |
| 3D | Direct effects of Transit stop proximity | Interaction with sex | 1.00 | 0.82, 1.23 | 0.995 |
| 4T | Total effects of Recreational facilities^2^ | Interaction with sex | 0.85 | 0.65, 1.11 | 0.239 |
| 4D | Direct effects of Recreational facilities^2^ | Interaction with sex | 0.85 | 0.65, 1.11 | 0.239 |
| 5T | Total effects of Park proximity | Interaction with sex | 0.87 | 0.72, 1.05 | 0.136 |
| 5D | Direct effects of Park proximity | Interaction with sex | 0.88 | 0.72, 1.06 | 0.168 |
| 6T | Total effects of Accessibility and walking facilities | Interaction with sex | 1.04 | 0.69, 1.55 | 0.867 |
| 6D | Direct effects of Accessibility and walking facilities | Interaction with sex | 1.04 | 0.69, 1.55 | 0.867 |
| 7T | Total effects of Traffic safety | Interaction with sex | 1.28 | 0.90, 1.81 | 0.166 |
| 7D | Direct effects of Traffic safety | Interaction with sex | 1.28 | 0.90, 1.81 | 0.166 |
| 8T | Total effects of Pedestrians infrastructure | Interaction with sex | 1.09 | 0.77, 1.54 | 0.618 |
| 8D | Direct effects of Pedestrians infrastructure | Interaction with sex | 1.09 | 0.77, 1.54 | 0.618 |
| 9T | Total effects of Safety from crime | Interaction with sex | 1.02 | 0.80, 1.31 | 0.857 |
| 9D | Direct effects of Safety from crime | Interaction with sex | 1.02 | 0.80, 1.31 | 0.857 |
| 10T | Total effects of Aesthetics | Interaction with sex | 1.00 | 0.75, 1.32 | 0.993 |
| 10D | Direct effects of Aesthetics | Interaction with sex | 1.00 | 0.75, 1.32 | 0.993 |
| 11T | Total effects of Buffers between street and footpath | Interaction with sex | 0.88 | 0.71, 1.08 | 0.206 |
| 11D | Direct effects of Buffers between street and footpath | Interaction with sex | 0.88 | 0.71, 1.08 | 0.210 |
| 12T | Total effects of Parking difficult | Interaction with sex | **0.78** | **0.63, 0.97** | **0.023** |
|  |  | Male | 1.18 | 1.00, 1.39 | 0.057 |
|  |  | Female | 0.91 | 0.78, 1.07 | 0.258 |
| 12D | Direct effects of Parking difficult | Interaction with sex | **0.78** | **0.63, 0.97** | **0.023** |
|  |  | Male | 1.18 | 1.00, 1.39 | 0.057 |
|  |  | Female | 0.91 | 0.78, 1.07 | 0.258 |
| 13T | Total effects of Trees | Interaction with sex | 0.88 | 0.69, 1.11 | 0.284 |
| 13D | Direct effects of Trees | Interaction with sex | 0.87 | 0.68, 1.11 | 0.262 |
| 14T | Total effects of Distance to school | Interaction with sex | 0.95 | 0.77, 1.17 | 0.640 |
| 14D | Direct effects of Distance to school | Interaction with sex | 0.95 | 0.77, 1.17 | 0.640 |

*Notes.* ^1^ excluding transit stops; ^2^ excluding parks; OR = odd ratio; CI = confidence interval; in bold: effects significant at *p*<0.05.

# **Table S40.** Adolescents’ sex as a moderator of total and direct effects of perceived neighbourhood environment characteristics on adolescents’ cycling to and from school vs. no active transport to/from school (reference category) (2 latent classes) [complete case analyses]

| Model | Environmental effect estimated | Regression coefficient | ***OR*** | **95% CI** | ***p-value*** |
| --- | --- | --- | --- | --- | --- |
| 1T | Total effects of Residential density | Interaction with sex | **1.002** | **1.000, 1.004** | **0.031** |
|  |  | Male | **0.998** | **0.996, 0.999** | **0.048** |
|  |  | Female | 1.000 | 0.999, 1.002 | 0.620 |
| 1D | Direct effects of Residential density | Interaction with sex | 1.002 | 1.00, 1.004 | 0.052 |
|  |  | Male | 0.998 | 0.997, 1.000 | 0.115 |
|  |  | Female | 1.001 | 0.999, 1.002 | 0.559 |
| 2T | Total effects of Land use mix diversity^1^ | Interaction with sex | **1.61** | **1.08, 2.41** | **0.019** |
|  |  | Male | 1.01 | 0.78, 1.33 | 0.915 |
|  |  | Female | **1.64** | **1.19, 2.26** | **0.003** |
| 2D | Direct effects of Land use mix diversity^1^ | Interaction with sex | **1.68** | **1.11, 2.54** | **0.014** |
|  |  | Male | 1.02 | 0.73, 1.41 | 0.922 |
|  |  | Female | **1.71** | **1.17, 2.48** | **0.005** |
| 3T | Total effects of Transit stop proximity | Interaction with sex | 1.05 | 0.77, 1.45 | 0.743 |
| 3D | Direct effects of Transit stop proximity | Interaction with sex | 1.05 | 0.76, 1.46 | 0.761 |
| 4T | Total effects of Recreational facilities^2^ | Interaction with sex | **1.72** | **1.18, 2.50** | **0.005** |
|  |  | Male | **0.71** | **0.51, 1.00** | **0.049** |
|  |  | Female | 1.22 | 0.86, 1.73 | 0.257 |
| 4D | Direct effects of Recreational facilities^2^ | Interaction with sex | **1.72** | **1.18, 2.50** | **0.005** |
|  |  | Male | **0.71** | **0.51, 1.00** | **0.049** |
|  |  | Female | 1.22 | 0.86, 1.73 | 0.257 |
| 5T | Total effects of Park proximity | Interaction with sex | **1.33** | **1.02, 1.74** | **0.034** |
|  |  | Male | **0.78** | **0.63, 0.97** | **0.027** |
|  |  | Female | 1.04 | 0.84, 1.29 | 0.711 |
| 5D | Direct effects of Park proximity | Interaction with sex | **1.31** | **1.00, 1.72** | **0.049** |
|  |  | Male | **0.77** | **0.62, 0.96** | **0.023** |
|  |  | Female | 1.01 | 0.81, 1.26 | 0.913 |
| 6T | Total effects of Accessibility and walking facilities | Interaction with sex | 1.70 | 0.97, 2.98 | 0.066 |
| 6D | Direct effects of Accessibility and walking facilities | Interaction with sex | 1.70 | 0.97, 2.98 | 0.066 |
| 7T | Total effects of Traffic safety | Interaction with sex | 1.07 | 0.66, 1.74 | 0.785 |
| 7D | Direct effects of Traffic safety | Interaction with sex | 1.07 | 0.66, 1.74 | 0.785 |
| 8T | Total effects of Pedestrians infrastructure | Interaction with sex | 1.05 | 0.64, 1.70 | 0.854 |
| 8D | Direct effects of Pedestrians infrastructure | Interaction with sex | 1.05 | 0.64, 1.70 | 0.854 |
| 9T | Total effects of Safety from crime | Interaction with sex | 1.13 | 0.78, 1.63 | 0.529 |
| 9D | Direct effects of Safety from crime | Interaction with sex | 1.13 | 0.78, 1.63 | 0.529 |
| 10T | Total effects of Aesthetics | Interaction with sex | 1.01 | 0.69, 1.48 | 0.963 |
| 10D | Direct effects of Aesthetics | Interaction with sex | 1.01 | 0.69, 1.48 | 0.963 |
| 11T | Total effects of Buffers between street and footpath | Interaction with sex | 0.88 | 0.65, 1.21 | 0.441 |
| 11D | Direct effects of Buffers between street and footpath | Interaction with sex | 0.88 | 0.64, 1.20 | 0.413 |
| 12T | Total effects of Parking difficult | Interaction with sex | 1.24 | 0.92, 1.68 | 0.162 |
| 12D | Direct effects of Parking difficult | Interaction with sex | 1.24 | 0.92, 1.68 | 0.162 |
| 13T | Total effects of Trees | Interaction with sex | 1.18 | 0.87, 1.59 | 0.278 |
| 13D | Direct effects of Trees | Interaction with sex | 1.18 | 0.87, 1.60 | 0.292 |
| 14T | Total effects of Distance to school | Interaction with sex | 0.99 | 0.73, 1.33 | 0.936 |
| 14D | Direct effects of Distance to school | Interaction with sex | 0.99 | 0.73, 1.33 | 0.936 |

*Notes.* ^1^ excluding transit stops; ^2^ excluding parks; OR = odd ratio; CI = confidence interval; in bold: effects significant at *p*<0.05

# **Table S41.** Adolescents’ sex as a moderator of total and direct effects of perceived neighbourhood environment characteristics on adolescents’ walking to and from school vs. walking from school (reference category). (2 latent profiles)

| Model | Environmental effect estimated | Regression coefficient | ***OR*** | **95% CI** | ***p-value*** |
| --- | --- | --- | --- | --- | --- |
| 1T | Total effects of Residential density | Interaction with sex | 1.001 | 0.999, 1.002 | 0.061 |
| 1D | Direct effects of Residential density | Interaction with sex | 1.001 | 0.999, 1.002 | 0.144 |
| 2T | Total effects of Land use mix diversity^1^ | Interaction with sex | 1.21 | 0.89, 1.64 | 0.231 |
| 2D | Direct effects of Land use mix diversity^1^ | Interaction with sex | 1.23 | 0.90, 1.69 | 0.196 |
| 3T | Total effects of Transit stop proximity | Interaction with sex | 0.99 | 0.79, 1.25 | 0.942 |
| 3D | Direct effects of Transit stop proximity | Interaction with sex | 1.00 | 0.79, 1.26 | 0.998 |
| 4T | Total effects of Recreational facilities^2^ | Interaction with sex | 1.20 | 0.90, 1.62 | 0.213 |
| 4D | Direct effects of Recreational facilities^2^ | Interaction with sex | 1.20 | 0.90, 1.62 | 0.213 |
| 5T | Total effects of Park proximity | Interaction with sex | 1.20 | 0.98, 1.48 | 0.080 |
| 5D | Direct effects of Park proximity | Interaction with sex | 1.20 | 0.97, 1.47 | 0.093 |
| 6T | Total effects of Accessibility and walking facilities | Interaction with sex | 1.10 | 0.70, 1.73 | 0.680 |
| 6D | Direct effects of Accessibility and walking facilities | Interaction with sex | 1.10 | 0.70, 1.73 | 0.680 |
| 7T | Total effects of Traffic safety | Interaction with sex | 0.85 | 0.58, 1.24 | 0.401 |
| 7D | Direct effects of Traffic safety | Interaction with sex | 0.85 | 0.58, 1.24 | 0.401 |
| 8T | Total effects of Pedestrians infrastructure | Interaction with sex | 0.88 | 0.60, 1.31 | 0.543 |
| 8D | Direct effects of Pedestrians infrastructure | Interaction with sex | 0.88 | 0.60, 1.31 | 0.543 |
| 9T | Total effects of Safety from crime | Interaction with sex | 0.93 | 0.71, 1.21 | 0.572 |
| 9D | Direct effects of Safety from crime | Interaction with sex | 0.93 | 0.71, 1.21 | 0.572 |
| 10T | Total effects of Aesthetics | Interaction with sex | 1.17 | 0.86, 1.59 | 0.317 |
| 10D | Direct effects of Aesthetics | Interaction with sex | 1.17 | 0.86, 1.59 | 0.317 |
| 11T | Total effects of Buffers between street and footpath | Interaction with sex | 1.17 | 0.93, 1.47 | 0.191 |
| 11D | Direct effects of Buffers between street and footpath | Interaction with sex | 1.16 | 0.92, 1.46 | 0.214 |
| 12T | Total effects of Parking difficult | Interaction with sex | **1.47** | **1.15, 1.88** | **0.002** |
|  |  | Male | 0.85 | 0.71, 1.03 | 0.091 |
|  |  | Female | **1.26** | **1.05, 1.50** | **0.011** |
| 12D | Direct effects of Parking difficult | Interaction with sex | **1.47** | **1.15, 1.88** | **0.002** |
|  |  | Male | 0.85 | 0.71, 1.03 | 0.091 |
|  |  | Female | **1.26** | **1.05, 1.50** | **0.011** |
| 13T | Total effects of Trees | Interaction with sex | 1.21 | 0.93, 1.57 | 0.153 |
| 13D | Direct effects of Trees | Interaction with sex | 1.23 | 0.94, 1.60 | 0.130 |
| 14T | Total effects of Distance to school | Interaction with sex | 0.90 | 0.73, 1.12 | 0.364 |
| 14D | Direct effects of Distance to school | Interaction with sex | 0.90 | 0.73, 1.12 | 0.364 |

*Notes.* ^1^ excluding transit stops; ^2^ excluding parks; OR = odd ratio; CI = confidence interval; in bold: effects significant at *p*<0.05.

# **Table S42.** Difference in Akaike Information Criterion (AIC) values between models with and without city as a moderator of total/direct effects of perceived environment characteristics on adolescents’ active transport to/from school

| Model | Environmental effect estimated | ***AIC difference (interaction effects - main effects)*** |
| --- | --- | --- |
| 1T | Total effects of Residential density | 72.9 |
| 1D | Direct effects of Residential density | 79.3 |
| 2T | Total effects of Land use mix diversity^1^ | 106.2 |
| 2D | Direct effects of Land use mix diversity^1^ | 85.2 |
| 3T | Total effects of Transit stop proximity | 65.5 |
| 3D | Direct effects of Transit stop proximity | 61.9 |
| 4T | Total effects of Recreational facilities^2^ | 128.5 |
| 4D | Direct effects of Recreational facilities^2^ | 128.5 |
| 5T | Total effects of Park proximity | N/A* |
| 5D | Direct effects of Park proximity | N/A* |
| 6T | Total effects of Accessibility and walking facilities | 56.7 |
| 6D | Direct effects of Accessibility and walking facilities | 56.7 |
| 7T | Total effects of Traffic safety | 62.1 |
| 7D | Direct effects of Traffic safety | 62.1 |
| 8T | Total effects of Pedestrians infrastructure | 57.5 |
| 8D | Direct effects of Pedestrians infrastructure | 57.5 |
| 9T | Total effects of Safety from crime | 190.0 |
| 9D | Direct effects of Safety from crime | 190.0 |
| 10T | Total effects of Aesthetics | 116.6 |
| 10D | Direct effects of Aesthetics | 116.6 |
| 11T | Total effects of Buffers between street and footpath | 108.7 |
| 11D | Direct effects of Buffers between street and footpath | 106.4 |
| 12T | Total effects of Parking difficult | 1091 |
| 12D | Direct effects of Parking difficult | 1091 |
| 13T | Total effects of Trees | 68.4 |
| 13D | Direct effects of Trees | 70.1 |
| 14T | Total effects of Distance to school | 826.5 |
| 14D | Direct effects of Distance to school | 826.5 |

*Notes.* ^1^ excluding transit stops; ^2^ excluding parks; AIC=Akaike Information Criterion. *There is no variation in response of Nigeria, all the responses are 1. The reference category of the outcome variable is “not engaging in active transport to/from school”.

# **Table S43.** Difference in Akaike Information Criterion (AIC) values between models with and without city as a moderator of total/direct effects of perceived environment characteristics on adolescents’ regular cycling to/from school (excluding Israel, Portugal and Czech Rep-Olomouc)

| Model | Environmental effect estimated | ***AIC difference (interaction effects - main effects)*** |
| --- | --- | --- |
| 1T | Total effects of Residential density | 420.7 |
| 1D | Direct effects of Residential density | 446.0 |
| 2T | Total effects of Land use mix diversity^1^ | 683.1 |
| 2D | Direct effects of Land use mix diversity^1^ | 701.8 |
| 3T | Total effects of Transit stop proximity | 2023.2 |
| 3D | Direct effects of Transit stop proximity | 1976.0 |
| 4T | Total effects of Recreational facilities^2^ | 162.0 |
| 4D | Direct effects of Recreational facilities^2^ | 162.0 |
| 5T | Total effects of Park proximity | N/A* |
| 5D | Direct effects of Park proximity | N/A* |
| 6T | Total effects of Accessibility and walking facilities | 372.3 |
| 6D | Direct effects of Accessibility and walking facilities | 372.3 |
| 7T | Total effects of Traffic safety | 219.1 |
| 7D | Direct effects of Traffic safety | 219.1 |
| 8T | Total effects of Pedestrians infrastructure | 365.0 |
| 8D | Direct effects of Pedestrians infrastructure | 365.0 |
| 9T | Total effects of Safety from crime | 298.8 |
| 9D | Direct effects of Safety from crime | 298.8 |
| 10T | Total effects of Aesthetics | 530.8 |
| 10D | Direct effects of Aesthetics | 530.8 |
| 11T | Total effects of Buffers between street and footpath | 598.8 |
| 11D | Direct effects of Buffers between street and footpath | 585.0 |
| 12T | Total effects of Parking difficult | 148.3 |
| 12D | Direct effects of Parking difficult | 148.3 |
| 13T | Total effects of Trees | 4148.7 |
| 13D | Direct effects of Trees | 4087.3 |
| 14T | Total effects of Distance to school | 220.1 |
| 14D | Direct effects of Distance to school | 220.1 |

*Notes.*  ^1^ excluding transit stops; ^2^ excluding parks; AIC=Akaike Information Criterion. *There is no variation in responses of Nigeria, all responses are 1. Regular cycling means cycling to/from school 5 to 10 times a week. The reference category of the outcome variable is “not engaging in regular cycling to/from school”.

# **Table S44.** Difference in Akaike Information Criterion (AIC) values between models with and without city as a moderator of total/direct effects of perceived neighbourhood environment characteristics on adolescents’ regular walking to/from school

| Model | Environmental effect estimated | ***AIC difference (interaction effects - main effects)*** |
| --- | --- | --- |
| 1T | Total effects of Residential density | 66.7 |
| 1D | Direct effects of Residential density | 169.9 |
| 2T | Total effects of Land use mix diversity^1^ | 57.2 |
| 2D | **Direct effects of Land use mix diversity^1^** | **-47.8** |
| 3T | Total effects of Transit stop proximity | 80.2 |
| 3D | Direct effects of Transit stop proximity | 144.3 |
| 4T | Total effects of Recreational facilities^2^ | 126.7 |
| 4D | Direct effects of Recreational facilities^2^ | 126.7 |
| 5T | Total effects of Park proximity | N/A* |
| 5D | Direct effects of Park proximity | N/A* |
| 6T | Total effects of Accessibility and walking facilities | 57.0 |
| 6D | Direct effects of Accessibility and walking facilities | 57.0 |
| 7T | Total effects of Traffic safety | 54.2 |
| 7D | Direct effects of Traffic safety | 54.2 |
| 8T | Total effects of Pedestrians infrastructure | 60.2 |
| 8D | Direct effects of Pedestrians infrastructure | 60.2 |
| 9T | Total effects of Safety from crime | 66.0 |
| 9D | Direct effects of Safety from crime | 66.0 |
| 10T | Total effects of Aesthetics | 66.4 |
| 10D | Direct effects of Aesthetics | 66.4 |
| 11T | Total effects of Buffers between street and footpath | 51.9 |
| 11D | Direct effects of Buffers between street and footpath | 50.8 |
| 12T | **Total effects of Parking difficult** | **-49.1** |
| 12D | **Direct effects of Parking difficult** | **-49.1** |
| 13T | Total effects of Trees | 46.4 |
| 13D | Direct effects of Trees | 49.2 |
| 14T | Total effects of Distance to school | 649.5 |
| 14D | Direct effects of Distance to school | 649.5 |

*Notes.*  ^1^ excluding transit stops; ^2^ excluding parks; AIC=Akaike Information Criterion. *There is no variation in responses of Nigeria, all responses are 1. Regular walking means walking to/from school 5 to 10 times a week. The reference category of the outcome variable is “not engaging in regular walking to/from school”.

# **Table S45.** City as a moderator of the direct effects of perceived neighbourhood environment characteristics on adolescents’ regular walking to/from school

|  | **Land use mix - diversity** | | | **Parking difficult** | | |
| --- | --- | --- | --- | --- | --- | --- |
| City (country) | ***OR*** | **95% CI** | ***p-value*** | ***OR*** | **95% CI** | ***p-value*** |
| *High-income countries* |  |  |  |  |  |  |
| Melbourne (AUS) | **1.62** | **1.08, 2.43** | **0.020** | 1.40 | 0.99, 1.99 | 0.056 |
| Ghent (BEL) | 1.36 | 0.84, 2.21 | 0.213 | 1.37 | 0.94, 1.98 | 0.102 |
| Hradec Králové (CZE) | 1.65 | 0.79, 3.44 | 0.186 | 1.93 | 0.78, 4.77 | 0.155 |
| Olomouc (CZE) | **4.35** | **1.34, 14.14** | **0.014** | 1.06 | 0.44, 2.53 | 0.904 |
| Odense (DNK) | **1.89** | **1.14, 3.11** | **0.013** | 0.86 | 0.50, 1.47 | 0.585 |
| Hong Kong (HKG, China) | **1.39** | **1.14, 1.70** | **0.001** | 0.96 | 0.83, 1.12 | 0.648 |
| Haifa (ISR) | 1.34 | 0.90, 2.00 | 0.148 | 1.20 | 0.86, 1.67 | 0.282 |
| Various cities (PRT) | **2.11** | **1.16, 3.82** | **0.014** | **2.00** | **1.04, 3.85** | **0.038** |
| Valencia (ESP) | **2.36** | **1.39, 4.02** | **0.002** | 1.08 | 0.81, 1.43 | 0.613 |
| Baltimore (USA) | **1.41** | **1.00,2.00** | **0.052** | 1.27 | 0.92, 1.75 | 0.152 |
| Seattle (USA) | **1.46** | **1.05, 2.05** | **0.025** | 0.99 | 0.72, 1.36 | 0.944 |
| *Low-middle-income countries* |  |  |  |  |  |  |
| Dhaka (BGD) | 1.42 | 0.58, 3.52 | 0.444 | 0.87 | 0.42, 1.80 | 0.712 |
| Curitiba (BRA) | **1.47** | **1.05, 2.06** | **0.026** | 1.04 | 0.87, 1.23 | 0.695 |
| Chennai (IND) | 1.27 | 0.81, 1.99 | 0.304 | 1.01 | 0.81, 1.26 | 0.916 |
| Kuala Lumpur (MYS) | 1.28 | 0.89, 1.84 | 0.175 | 1.02 | 0.73, 1.43 | 0.889 |
| Gombe (NGA) | 1.31 | 0.85, 2.02 | 0.226 | 0.93 | 0.72, 1.20 | 0.566 |

*Notes.* OR = odd ratio; CI = confidence interval; in bold: effects significant at *p*<0.05. Regular walking means walking to/from school 5 to 10 times a week. The reference category of the outcome variable is “not engaging in regular walking to/from school”.

# **Table S46.** Difference in Akaike Information Criterion (AIC) values between models with and without city as a moderator of total/direct effects of perceived neighbourhood environment characteristics on adolescents’ walking to and from school vs. no active transport to/from school (reference category) (2 latent profiles)

| Model | Environmental effect estimated | ***AIC difference (interaction effects - main effects)*** |
| --- | --- | --- |
| 1T | Total effects of Residential density | 65.5 |
| 1D | Direct effects of Residential density | 802.6 |
| 2T | Total effects of Land use mix diversity^1^ | 76.1 |
| 2D | Direct effects of Land use mix diversity^1^ | **-2034.8** |
| 3T | Total effects of Transit stop proximity | 69.8 |
| 3D | Direct effects of Transit stop proximity | 1191.2 |
| 4T | Total effects of Recreational facilities^2^ | 572.8 |
| 4D | Direct effects of Recreational facilities^2^ | 572.8 |
| 5T | Total effects of Park proximity | N/A* |
| 5D | Direct effects of Park proximity | N/A* |
| 6T | Total effects of Accessibility and walking facilities | 68.7 |
| 6D | Direct effects of Accessibility and walking facilities | 68.7 |
| 7T | Total effects of Traffic safety | 66.3 |
| 7D | Direct effects of Traffic safety | 66.3 |
| 8T | Total effects of Pedestrians infrastructure | 71.9 |
| 8D | Direct effects of Pedestrians infrastructure | 71.9 |
| 9T | Total effects of Safety from crime | 92.2 |
| 9D | Direct effects of Safety from crime | 92.2 |
| 10T | Total effects of Aesthetics | 84.5 |
| 10D | Direct effects of Aesthetics | 84.5 |
| 11T | Total effects of Buffers between street and footpath | 67.5 |
| 11D | Direct effects of Buffers between street and footpath | 68.3 |
| 12T | Total effects of Parking difficult | **-1005.2** |
| 12D | Direct effects of Parking difficult | **-1005.2** |
| 13T | Total effects of Trees | 57.4 |
| 13D | Direct effects of Trees | 60.9 |
| 14T | Total effects of Distance to school | 637.9 |
| 14D | Direct effects of Distance to school | 637.9 |

*Notes.* ^1^ excluding transit stops; ^2^ excluding parks *There is no variation in responses of Nigeria, all responses are 1.

# **Table S47.** City as a moderator of direct effects of perceived neighbourhood environment characteristics on adolescents’ walking to and from school vs. no active transport to/from school (reference category) (2 latent profiles)

|  | **Land use mix - diversity** | | | **Parking difficult** | | |
| --- | --- | --- | --- | --- | --- | --- |
| City (country) | ***OR*** | **95% CI** | ***p-value*** | ***OR*** | **95% CI** | ***p-value*** |
| *High-income countries* |  |  |  |  |  |  |
| Melbourne (AUS) | **2.39** | **1.20, 4.76** | **0.013** | 0.90 | 0.51, 1.61 | 0.728 |
| Ghent (BEL) | 1.72 | 0.87, 3.39 | 0.119 | 1.06 | 0.63, 1.77 | 0.834 |
| Hradec Králové (CZE) | 1.72 | 0.76, 3.90 | 0.195 | 2.20 | 0.80, 6.05 | 0.128 |
| Olomouc (CZE) | **4.43** | **1.33, 14.76** | **0.015** | 1.10 | 0.44, 2.74 | 0.834 |
| Odense (DNK) | **2.70** | **1.20, 6.07** | **0.016** | 1.00 | 0.38, 2.62 | 0.996 |
| Hong Kong (HKG, China) | **1.46** | **1.17, 1.81** | **0.001** | 0.98 | 0.83, 1.15 | 0.767 |
| Haifa (ISR) | 1.47 | 0.92, 2.33 | 0.104 | **1.54** | **1.02, 2.32** | **0.041** |
| Various cities (PRT) | **2.28** | **1.20, 4.34** | **0.012** | **2.64** | **1.22, 5.71** | **0.014** |
| Valencia (ESP) | **3.14** | **1.70, 5.81** | **<0.001** | 1.11 | 0.80, 1.54 | 0.531 |
| Baltimore (USA) | 1.47 | 0.97, 2.25 | 0.071 | **1.55** | **1.04, 2.31** | **0.030** |
| Seattle (USA) | **1.73** | **1.15, 2.59** | **0.008** | 0.87 | 0.59, 1.29 | 0.483 |
| *Low-middle-income countries* |  |  |  |  |  |  |
| Dhaka (BGD) | 0.66 | 0.20, 2.24 | 0.509 | 0.66 | 0.26, 1.67 | 0.381 |
| Curitiba (BRA) | **1.73** | **1.18, 2.53** | **0.005** | 1.00 | 0.83, 1.21 | 0.973 |
| Chennai (IND) | 1.66 | 0.96, 2.89 | 0.071 | 1.06 | 0.81, 1.39 | 0.669 |
| Kuala Lumpur (MYS) | **1.66** | **1.07, 2.57** | **0.023** | 0.85 | 0.56, 1.30 | 0.455 |
| Gombe (NGA) | 1.16 | 0.73, 1.85 | 0.537 | 0.96 | 0.73, 1.27 | 0.793 |

*Notes.* OR = odd ratio; CI = confidence interval; in bold: effects significant at *p*<0.05.

# **Table S48.** Difference in Akaike Information Criterion (AIC) values between models with and without city as a moderator of total/direct effects of perceived environment characteristics on adolescents’ walking from school vs. no active transport to/from school (reference category) (2 latent classes)

| Model | Environmental effect estimated | ***AIC difference (interaction effects - main effects)*** |
| --- | --- | --- |
| 1T | Total effects of Residential density | 69.8 |
| 1D | Direct effects of Residential density | 69.0 |
| 2T | Total effects of Land use mix diversity^1^ | 76.8 |
| 2D | Direct effects of Land use mix diversity^1^ | 69.7 |
| 3T | Total effects of Transit stop proximity | 213.6 |
| 3D | Direct effects of Transit stop proximity | 221.5 |
| 4T | Total effects of Recreational facilities^2^ | 73.3 |
| 4D | Direct effects of Recreational facilities^2^ | 73.3 |
| 5T | Total effects of Park proximity | N/A* |
| 5D | Direct effects of Park proximity | N/A* |
| 6T | Total effects of Accessibility and walking facilities | 87.3 |
| 6D | Direct effects of Accessibility and walking facilities | 87.3 |
| 7T | Total effects of Traffic safety | 100.4 |
| 7D | Direct effects of Traffic safety | 100.4 |
| 8T | Total effects of Pedestrians infrastructure | 148.0 |
| 8D | Direct effects of Pedestrians infrastructure | 148.0 |
| 9T | Total effects of Safety from crime | 243.8 |
| 9D | Direct effects of Safety from crime | 243.8 |
| 10T | Total effects of Aesthetics | 64.0 |
| 10D | Direct effects of Aesthetics | 64.0 |
| 11T | Total effects of Buffers between street and footpath | 329.1 |
| 11D | Direct effects of Buffers between street and footpath | 316.1 |
| 12T | Total effects of Parking difficult | 345.7 |
| 12D | Direct effects of Parking difficult | 345.7 |
| 13T | Total effects of Trees | 54.5 |
| 13D | Direct effects of Trees | 52.7 |
| 14T | Total effects of Distance to school | 104.4 |
| 14D | Direct effects of Distance to school | 104.4 |

*Notes.* ^1^ excluding transit stops; ^2^ excluding parks; *There is no variation in responses of Nigeria, all responses are 1.

# **Table S49.** Difference in Akaike Information Criterion (AIC) values between models with and without city as a moderator of total/direct effects of perceived environment characteristics on adolescents’ cycling to/from school vs. no active transport to/from school (reference category) (2 latent classes)

| Model | Environmental effect estimated | ***AIC difference (interaction effects - main effects)*** |
| --- | --- | --- |
| 1T | Total effects of Residential density | 306.9 |
| 1D | Direct effects of Residential density | 242.8 |
| 2T | Total effects of Land use mix diversity^1^ | 945.4 |
| 2D | Direct effects of Land use mix diversity^1^ | 400.2 |
| 3T | Total effects of Transit stop proximity | 1452.5 |
| 3D | Direct effects of Transit stop proximity | 1277.8 |
| 4T | Total effects of Recreational facilities^2^ | 217.7 |
| 4D | Direct effects of Recreational facilities^2^ | 217.7 |
| 5T | Total effects of Park proximity | N/A* |
| 5D | Direct effects of Park proximity | N/A* |
| 6T | Total effects of Accessibility and walking facilities | 249.3 |
| 6D | Direct effects of Accessibility and walking facilities | 249.3 |
| 7T | Total effects of Traffic safety | 181.0 |
| 7D | Direct effects of Traffic safety | 181.0 |
| 8T | Total effects of Pedestrians infrastructure | 169.8 |
| 8D | Direct effects of Pedestrians infrastructure | 169.8 |
| 9T | Total effects of Safety from crime | 720.7 |
| 9D | Direct effects of Safety from crime | 720.7 |
| 10T | Total effects of Aesthetics | 450.6 |
| 10D | Direct effects of Aesthetics | 450.6 |
| 11T | Total effects of Buffers between street and footpath | 379.7 |
| 11D | Direct effects of Buffers between street and footpath | 373.2 |
| 12T | Total effects of Parking difficult | 138.1 |
| 12D | Direct effects of Parking difficult | 138.1 |
| 13T | Total effects of Trees | 3194.3 |
| 13D | Direct effects of Trees | 3522.3 |
| 14T | Total effects of Distance to school | 344.8 |
| 14D | Direct effects of Distance to school | 344.8 |

*Notes.* ^1^ excluding transit stops; ^2^ excluding parks; *There is no variation in responses of Nigeria, all responses are 1.

# **Table S50.** Difference in Akaike Information Criterion (AIC) values between models with and without city as a moderator of total/direct effects of perceived neighbourhood environment characteristics on adolescents’ walking to and from school vs. walking from school (reference category) (2 latent profiles)

| Model | Environmental effect estimated | ***AIC difference (interaction effects - main effects)*** |
| --- | --- | --- |
| 1T | Total effects of Residential density | 101.9 |
| 1D | Direct effects of Residential density | 112.7 |
| 2T | Total effects of Land use mix diversity^1^ | 109.5 |
| 2D | Direct effects of Land use mix diversity^1^ | 90.3 |
| 3T | Total effects of Transit stop proximity | 492.5 |
| 3D | Direct effects of Transit stop proximity | 502.5 |
| 4T | Total effects of Recreational facilities^2^ | 77.3 |
| 4D | Direct effects of Recreational facilities^2^ | 77.3 |
| 5T | Total effects of Park proximity | N/A* |
| 5D | Direct effects of Park proximity | N/A* |
| 6T | Total effects of Accessibility and walking facilities | 862.1 |
| 6D | Direct effects of Accessibility and walking facilities | 862.1 |
| 7T | Total effects of Traffic safety | 110.8 |
| 7D | Direct effects of Traffic safety | 110.8 |
| 8T | Total effects of Pedestrians infrastructure | 193.2 |
| 8D | Direct effects of Pedestrians infrastructure | 193.2 |
| 9T | Total effects of Safety from crime | 323.4 |
| 9D | Direct effects of Safety from crime | 323.4 |
| 10T | Total effects of Aesthetics | 57.3 |
| 10D | Direct effects of Aesthetics | 57.3 |
| 11T | Total effects of Buffers between street and footpath | 354.2 |
| 11D | Direct effects of Buffers between street and footpath | 359.5 |
| 12T | Total effects of Parking difficult | 642.5 |
| 12D | Direct effects of Parking difficult | 642.5 |
| 13T | Total effects of Trees | 132.1 |
| 13D | Direct effects of Trees | 141.2 |
| 14T | Total effects of Distance to school | 488.7 |
| 14D | Direct effects of Distance to school | 488.7 |

*Notes.* ^1^ excluding transit stops; ^2^ excluding parks; *There is no variation in responses of Nigeria, all responses are 1.
